# *BET1* variants establish impaired vesicular transport as a cause for muscular dystrophy with epilepsy

**DOI:** 10.15252/emmm.202013787

**Published:** 2021-11-15

**Authors:** Sandra Donkervoort, Niklas Krause, Mykola Dergai, Pomi Yun, Judith Koliwer, Svetlana Gorokhova, Janelle Geist Hauserman, Beryl B Cummings, Ying Hu, Rosemarie Smith, Prech Uapinyoying, Vijay S Ganesh, Partha S Ghosh, Kristin G Monaghan, Seby L Edassery, Pia E Ferle, Sarah Silverstein, Katherine R Chao, Molly Snyder, Sara Ellingwood, Diana Bharucha‐Goebel, Susan T Iannaccone, Matteo Dal Peraro, A Reghan Foley, Jeffrey N Savas, Véronique Bolduc, Dirk Fasshauer, Carsten G Bönnemann, Michael Schwake

**Affiliations:** ^1^ Neuromuscular and Neurogenetic Disorders of Childhood Section National Institute of Neurological Disorders and Stroke National Institutes of Health Bethesda MD USA; ^2^ Biochemistry III/Faculty of Chemistry Bielefeld University Bielefeld Germany; ^3^ Department of Fundamental Neurosciences University of Lausanne Lausanne Switzerland; ^4^ Service de Génétique Médicale Hôpital de la Timone, APHM Marseille France; ^5^ INSERM, U1251‐MMG Aix‐Marseille Université Marseille France; ^6^ Center for Mendelian Genomics Program in Medical and Population Genetics Broad Institute of MIT and Harvard Cambridge MA USA; ^7^ Maine Medical Center Portland ME USA; ^8^ Research for Genetic Medicine Children's National Medical Center Washington DC USA; ^9^ Department of Neurology Brigham & Women's Hospital Harvard Medical School Boston MA USA; ^10^ Department of Neurology Boston Children's Hospital Boston MA USA; ^11^ GeneDx Gaithersburg MD USA; ^12^ Department of Neurology Feinberg School of Medicine Northwestern University Chicago IL USA; ^13^ Rutgers New Jersey School of Medicine Newark NJ USA; ^14^ Undiagnosed Diseases Program National Human Genome Research Institute National Institute of Health Bethesda MD USA; ^15^ Department of Neurology Children's Health Dallas TX USA; ^16^ Division of Neurology Children’s National Medical Center Washington DC USA; ^17^ Division of Pediatric Neurology Departments of Pediatrics, Neurology and Neurotherapeutics University of Texas Southwestern Medical Center Dallas TX USA; ^18^ Institute of Bioengineering School of Life Sciences École Polytechnique Fédérale de Lausanne (EPFL) Lausanne Switzerland

**Keywords:** BET1, epilepsy, GOSR2, muscular dystrophy, SNARE, Genetics, Gene Therapy & Genetic Disease, Musculoskeletal System, Neuroscience

## Abstract

BET1 is required, together with its SNARE complex partners GOSR2, SEC22b, and Syntaxin‐5 for fusion of endoplasmic reticulum‐derived vesicles with the ER‐Golgi intermediate compartment (ERGIC) and the *cis*‐Golgi. Here, we report three individuals, from two families, with severe congenital muscular dystrophy (CMD) and biallelic variants in *BET1* (P1 p.(Asp68His)/p.(Ala45Valfs*2); P2 and P3 homozygous p.(Ile51Ser)). Due to aberrant splicing and frameshifting, the variants in P1 result in low BET1 protein levels and impaired ER‐to‐Golgi transport. Since *in silico* modeling suggested that p.(Ile51Ser) interferes with binding to interaction partners other than SNARE complex subunits, we set off and identified novel BET1 interaction partners with low affinity for p.(Ile51Ser) BET1 protein compared to wild‐type, among them ERGIC‐53. The BET1/ERGIC‐53 interaction was validated by endogenous co‐immunoprecipitation with both proteins colocalizing to the ERGIC compartment. Mislocalization of ERGIC‐53 was observed in P1 and P2’s derived fibroblasts; while in the p.(Ile51Ser) P2 fibroblasts specifically, mutant BET1 was also mislocalized along with ERGIC‐53. Thus, we establish *BET1* as a novel CMD/epilepsy gene and confirm the emerging role of ER/Golgi SNAREs in CMD.

## Graphical abstract

This study describes three individuals with a progressive early‐onset congenital muscular dystrophy, and additional epilepsy in one, caused by biallelic variants in the *BET1* gene. BET1, along with its SNARE complex partners, is essential for ER‐to‐Golgi trafficking.

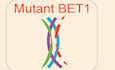

**The paper explained**

**Problem**

Congenital muscular dystrophies (CMDs) encompass a clinically and genetically heterogeneous group of degenerative disorders characterized by early‐onset weakness with dystrophic findings on muscle histology but that can also present with central nervous system (CNS) dysfunction in specific genetic subtypes. At this time, approximately 50% of patients with CMD remain without a confirmed genetic diagnosis. Recently, it has been suggested that genes responsible for the membrane trafficking machinery in cells, the ER‐to‐Golgi SNARE complex, may be a potential cause for CMD; however, this has not yet been confirmed.

**Results**

We used exome sequencing to identify recessively inherited variants in *BET1* in three patients with unknown neuromuscular disease. BET1 is part of the ER‐to‐Golgi SNARE complex for which a disease association has not yet been recognized. We identified a missense variant (c.202G>C; p.(Asp68His)) in compound heterozygosity with a truncating variant (c.134delC; p.(Ala45Valfs*2)) in one individual and a missense variant (c.152T>G; p.(Ile51Ser)) in homozygosity in siblings. All individuals were found to have a severe, early‐onset muscular dystrophy with epilepsy in one. We showed that the (c.202G>C; p.(Asp68His)) missense acts as a complex splice variant causing a reduction of BET1 protein in patient cells with impaired vesicular traffic. Analysis of cells from the second patient with the p.(Ile51Ser) missense variant identified normal levels of BET1 protein. We subsequently studied this variant further and identified ERGIC‐53 as a novel interaction partner of BET1. Moreover, we found that the p.(Ile51Ser) BET1 missense resulted in impaired interaction with ERGIC‐53.

**Impact**

This study reports the first affected individuals with variants in the *BET1* gene. We provide evidence to show that these variants are disease causing. Identification and characterization of these variants is important for providing accurate recurrence risk, prognosis, and medical management for patients and their families. Additionally, variants in *BET1* can now be screened in patients with unknown neuromuscular disease and/or epilepsy. Furthermore, insights from this study help in understanding the underlying mechanism of the membrane trafficking machinery both in healthy cells and in the setting of disease.

## Introduction

Congenital muscular dystrophies (CMDs) encompass a clinically and genetically heterogeneous group of degenerative disorders, predominantly of muscle, characterized by early‐onset weakness with dystrophic findings on muscle histology but that can also present with central nervous system (CNS) dysfunction in specific genetic subtypes (Bonnemann *et al*, 2014; Schorling *et al*, 2017). Although heterogeneous, the CMDs can be classified by the cellular localization and function of the affected proteins. For example, pathogenic variants in extracellular matrix proteins have been identified (e.g., collagen VI (MIM:120220, 120240, 120250) and laminin α2 (MIM:156225), the extracellular matrix and cytoskeleton anchor dystrophin (MIM: 300376 and 310200), the cell surface dystrophin‐associated glycoprotein dystroglycan (DAG1, MIM: 616538 and 613818), glycosyltransferases (POMT1 (MIM: 607423), POMT2 (MIM: 607439)) and those of the nuclear envelope and cytoskeleton (Lamin A/C (MIM:150330)) (Schorling *et al*, 2017; Dowling *et al*, 2021). Very recently, variants affecting components of membrane trafficking machinery (TRAPPC11 (MIM:614138) and GOSR2 (MIM:604027)) have been added to the list of inherited CMDs (Larson *et al*, 2018; Henige *et al*, 2021; Stemmerik *et al*, 2021).

GOSR2 and its complex partner BET1 belong to a group of SNARE (soluble N‐ethylmaleimide‐sensitive factor attachment protein receptor) proteins that are essential for docking and fusion of vesicle‐mediated membrane trafficking. These proteins form complexes consisting of four SNARE domains, derived from at least three SNAREs. SNAREs can be subcategorized into Q and R‐SNAREs depending on the presence of a glutamine (Q) or arginine (R) in the zero ionic (0) layer of the SNARE core complex. Generally, three Q(a,b,c)‐SNAREs and one R‐SNARE assemble into a functional complex (Fasshauer *et al*, 1998). Trafficking between the ER network, through the ER‐Golgi intermediate compartment (ERGIC), and the Golgi apparatus (Hay *et al*, 1998) relies on a conserved SNARE complex consisting of Q_b_‐SNARE GOSR2 that forms a complex with Q_a_‐SNARE Syntaxin‐5, Q_c_‐SNARE BET1, and the R‐SNARE SEC22b (Hay *et al*, 1997; Lowe *et al*, 1997). Biallelic variants in *GOSR2* are a known cause for neurodevelopmental disorders clinically manifesting with epilepsy, scoliosis, and congenital myasthenic syndromes with rare cases of concomitant muscular dystrophy reported (Corbett *et al*, 2011). However, the role of impaired SNARE‐mediated vesicle trafficking in causing disease and CMD specifically, is not well characterized (Stamberger *et al*, 2016; Volker *et al*, 2017; Rodriguez Cruz *et al*, 2018).

Here, we report three affected individuals of two independent families with biallelic variants in *BET1* encoding BET1 (MIM: 605456), which has not yet been associated with disease in humans. We present comprehensive data that biallelic variants in *BET1* cause a severe progressive CMD with CNS involvement and provide further insight into the pathomechanism of SNARE‐mediated inherited disorders of trafficking. We thus confirm the emerging role of ER‐to‐Golgi Q‐SNARE‐mediated transport in muscle homeostasis and disease and establish *BET1* as a novel gene for CMD/epilepsy.

## Results

### Case reports

**Figure 1:**
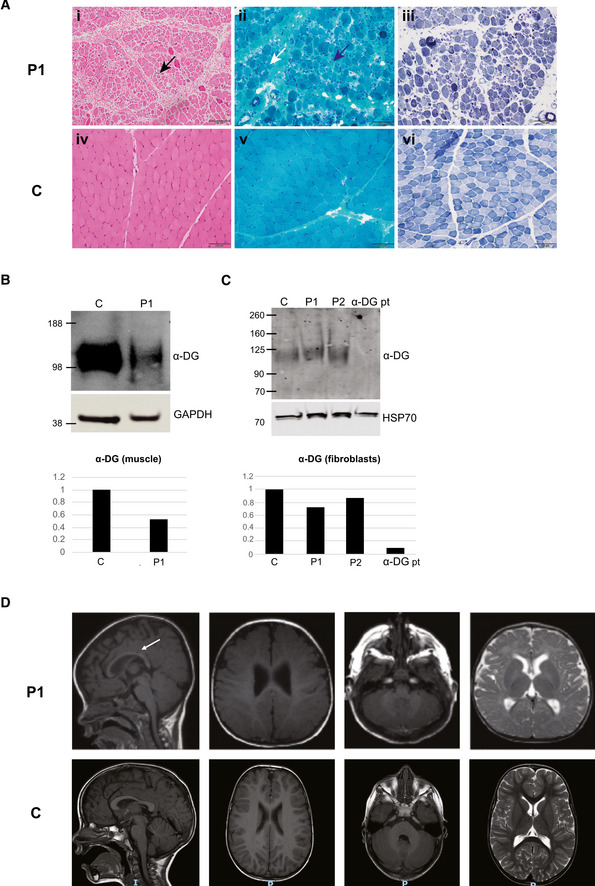
Clinical characteristics of P1 Left quadriceps muscle biopsy performed at age 10 months (i–iii) demonstrating a dystrophic process with variation in fiber size (black arrow), increased connective tissue (blue arrow), and rare internalized nuclei (white arrow) on hematoxylin and eosin (H&E) staining (i) and Gömöri trichrome staining (ii) and type 1 fiber atrophy on nicotinamide adenine dinucleotide (NADH) staining (iii). Muscle biopsy in an unaffected (control) individual (iv–vi). Scale bar: 100 μm.Western blot data (*n* = 1) with quantification demonstrated reduced α‐Dystroglycan (α‐DG) in P1’s muscle compared to control.Western blot data with quantification demonstrated reduced α‐Dystroglycan in fibroblasts from P1 and P2 compared to control (c), although not to the extent of the reduction observed in fibroblasts from a patient with a confirmed α‐dystroglycanopathy (α‐DG pt) due to biallelic pathogenic variants in *LARGE* (*n* = 1).Brain MRI findings in P1 at 28 months of age (top) revealing normal appearing cortex, cerebellum, and pons on T1‐weighted images and T2‐weighted image. There is evidence of mild thinning of the corpus callosum (arrow). Brain MRI in an unaffected (control) individual at 30 months of age (bottom) demonstrating normal‐sized corpus callosum. Source data are available online for this figure.

Individual 1 (P1) is a 5‐year‐old male born to non‐consanguineous parents. The pregnancy was notable for reduced fetal movements. He was born at 35 weeks gestation by spontaneous vaginal delivery and had feeding difficulties as a newborn that required the placement of a gastrostomy tube. At three months of age, he was noted to have poor head control and hypotonia. He eventually attained the ability to roll and sit unsupported at 12 months of age but did not achieve the ability to walk. Nighttime non‐invasive ventilation was initiated following the recognition of respiratory insufficiency during infancy. Examination at the age of 23 months revealed a high‐arched palate and restricted extraocular movements, particularly on upgaze. He had significant head lag with evidence of severe axial and appendicular weakness. He had scoliosis and contractures of the fingers, hips, knees, and ankles. His speech was delayed and was notable for poor articulation. Ophthalmological examination revealed high myopia with small cataracts bilaterally. Serum creatine kinase (CK) levels were markedly and consistently elevated (ranging 3,600–6,800 U/l). A muscle biopsy performed at age 10 months demonstrated findings consistent with a dystrophic process (Figure 1A). Western blot performed on P1’s muscle biopsy demonstrated reduced glycosylated α‐dystroglycan expression, which was not obvious in patient‐derived fibroblasts (Fig 1B and C). Muscle ultrasound at age 15 months was consistent with a primary myopathic disorder. Echocardiogram evaluations were normal. Brain MRI at the age of seven months was normal in appearance; however, a repeat brain MRI at 28 months of age demonstrated a mild increase in T2‐weighted white matter signal (Fig 1D). At four years of age, he required ventilatory support via a tracheostomy due to the progression of respiratory insufficiency. He demonstrated progressive, severe muscle weakness, and by approximately 3.5 years of age, he was no longer able to roll over or sit without support. By four years of age, antigravity movements were restricted to elbow flexion, ankle dorsiflexion, and plantarflexion. He developed progressive epilepsy at four years of age which was refractory despite multiple antiepileptic medications and a ketogenic diet. He subsequently lost the ability to babble and was noted to become increasingly lethargic. His symptoms continued to progress, and he passed away at five years of age.

We subsequently identified a second family through GeneMatcher with two affected siblings (P2, P3) (Sobreira *et al*, 2015). Individual 2 (P2) is a female born at term to non‐consanguineous parents who had previously lost a son (P3) with an undiagnosed CMD. Pregnancy was complicated by gestational diabetes. Birth was complicated by shoulder dystocia resulting in right‐sided brachial plexus palsy. She was noted to have low tone and tachypnea after birth raising concern that she had a similar condition as her deceased brother. She was admitted at age 11 days for hypotonia, stridor, and poor feeding, which required gastrostomy tube (G‐tube) placement. On examination at age six weeks, she had pronounced axial hypotonia but spontaneous antigravity movements of the extremities. Deep tendon reflexes were decreased in the legs. No dysmorphic features were noted. Upon re‐examination at eight weeks of age, she had decreased spontaneous movements and appeared more lethargic overall. CK was consistently elevated (ranging 436–2,361 U/l). A brain MRI performed at two weeks of age did not show structural abnormalities. EMG and an echocardiogram at age one month were normal. She was diagnosed with mild tracheomalacia and mild bronchomalacia, and by four months of age, she developed respiratory failure. She needed increasing levels of ventilatory support and passed away at four months of age.

Her deceased older brother (P3) had similar symptoms. He was born at term and was noted to be hypotonic. Initially, there were no concerns for breathing or feeding; however, he was re‐admitted at age four days due to persistent low tone. He was noted to have some mild dysmorphic features including a thin upper lip, preauricular ear tag, and single palmar crease. Deep tendon reflexes were intact. At age two months, there were concerns over failure to thrive. Given the concerns noted from fatigue during feeding, weak suck, and aspiration with choking, a G‐tube was placed. By four months of age, respiratory insufficiency and poor visual tracking were noted. Brain MRI at age four days did not reveal structural abnormalities. CK was consistently elevated (ranging 921–4,000s U/l). Bronchoscopy revealed severe glossoptosis, bronchomalacia, and oral secretions at age four months. Muscle biopsies showed non‐specific myopathic changes including moderate variation in fiber size and occasional atrophic fibers. He passed away at seven months following a respiratory event.

### Biallelic *BET1* variants in patients with CMD

**Figure EV1:**
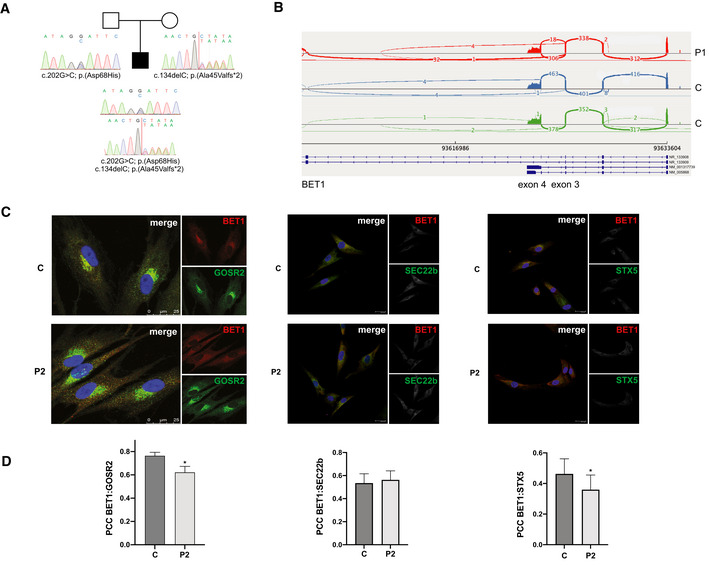
Genetic studies in P1 Pedigree of P1 with corresponding *BET1* Sanger chromatogram depicting the paternally inherited c.202G>C; p.(Asp68His) missense variant and a maternally inherited c.134delC; p.(Ala45Valfs*2) frameshift variant (NM_005868), consistent with recessive inheritance.Sashimi plots comparing fibroblast RNA sequencing reads in P1 (red) and two control muscle samples (blue and green) at the exons 3–5 of BET1.Immunofluorescence images of BET1 (red) and GOSR2 (green) in fibroblasts from P1, P2, and control. In P1, BET1 immunoreactivity is reduced. Scale bar: 25 μm.In the overlay, BET1 colocalization with GOSR2 and Syntaxin‐5 is reduced in P2 compared to controls while BET1 colocalization with SEC22b is unchanged. BET1 colocalization was determined using Pearson’s correlation coefficient (PCC) on regions of interest (ROIs) drawn around cells (GOSR2 P2: *n* = 56; C: *n* = 86, *P* = 0.0025) (Syntaxin‐5 P2: *n* = 27; C: *n* = 32 *P* = 0.0006) (SEC22b P2: *n* = 27; C: *n* = 30, *P* = 0.2093). (*P ≤ 0.05 Mann–Whitney *U*‐test). Data represented are mean ± SEM.

**Figure 2:**
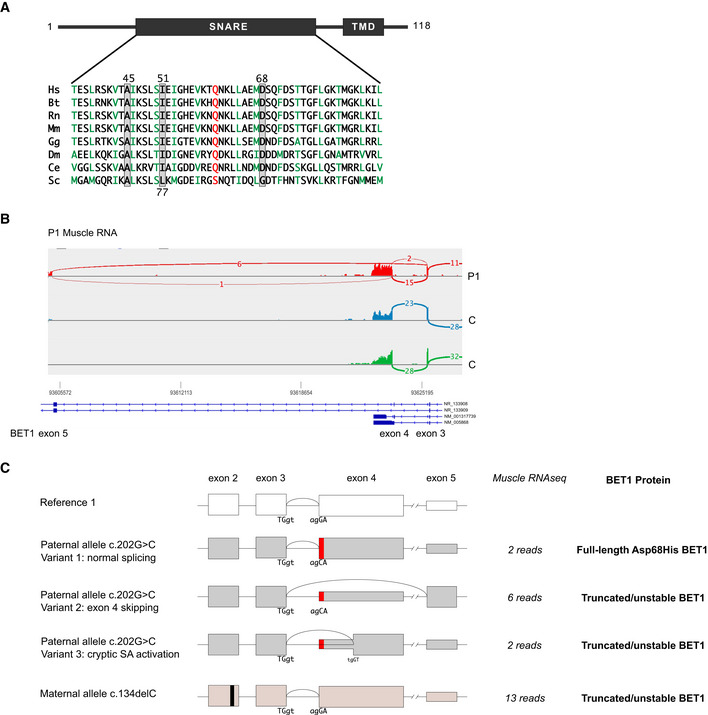
RNA sequencing analysis of the *BET1* c.202G>C; p.(Asp68His) missense variant Schematic depiction of BET1 indicating the position and conservation of the variants identified in this study. TMD = transmembrane domain.Sashimi plots comparing muscle RNA sequencing reads in P1 (red) and two control muscle samples (blue and green) at the exons 3–5 of BET1.Schematics of normal BET1 exon 3–4 splicing (top) and the various aberrant splice products associated with the paternally inherited p.(Asp68His) variant in P1. Splice variant one utilizes normal splicing generating a full‐length BET1 protein with the Asp68His substitution. Splice variant two skips exon 4 to non‐coding transcript (NR_133908) exon 5, resulting in a shift of the reading frame and a premature termination codon after 18 amino acids. Splice variant three utilizes a cryptic splice acceptor within exon 4, deleting the first 34 nucleotides of exon 4, and producing again a frameshift and a premature termination. The maternally inherited frameshift variant c.134delC introduces a premature stop codon in exon 2 and is predicted to induce nonsense‐mediated decay (NMD).

Next‐generation sequencing for genes known to cause muscular dystrophies, including α‐dystroglycanopathies, was negative in P1. Research‐based trio exome sequencing (ES) was then pursued to elucidate his genetic diagnosis. P1 was found to have compound heterozygous variants in *BET1* (GenBank: NM_005868.6; OMIM 605456): a maternally inherited frameshift (c.134delC; p.(Ala45Valfs*2)) and a paternally inherited missense variant (c.202G>C; p.(Asp68His)) (Fig EV1A and Table 1). The frameshift variant c.134delC introduces a premature stop codon after the 45^th^ amino acid residue in the second exon of a four‐exon transcript and is predicted to induce nonsense mediated decay (NMD). This variant has the popmax filtering allele frequency of 0.013% (highest population allele frequency of 0.018%, corresponding to 24/128800 alleles in the European non‐Finish population in gnomAD v.2.1.1, no homozygous individuals; Whiffin *et al*, 2017; Karczewski *et al*, 2021). The p.(Asp68His) missense is not present in the gnomADv2.1.1 database, is predicted to be damaging per various in silico prediction tools (MutationTaster: disease causing; CADD Prediction V1.4: 28.8), and affects a conserved aspartate residue (Fig 2A; Schwarz *et al*, 2014; Rentzsch *et al*, 2019). This missense variant is located at the first nucleotide of exon 4 (NM_005868.6 transcript) and is thus also predicted to cause a loss of the acceptor site (SpliceAI score of 0.42; Jaganathan *et al*, 2019). SpliceAI also predicts creation of a novel acceptor site 34 nucleotides (nt) into exon 4, though the score of 0.07 is below the threshold typically used to predict the impact on splicing.

**Table 1:** Patient’s clinical presentation and *BET1* variants identified.

	Family 1		Family 2		
	P1		P2		P3
Sex	Male		Female		Male
Age at time of death	5 years		4 months		7 months
Pathogenic variants					
*BET1* ^a^	c.202G>C; p. Asp68His Paternal (hg19) chr7:93623697C>G (hg38) chr7:93994385C>G	c.134delC p. Ala45ValfsTer2 Maternal (hg19) chr7:93628492delG (hg38) chr7:93999180delG	c.152T>G; p. Ile51Ser Homozygous (hg19) chr7:93625626A>C (hg38) chr7:93996314A>C		
GnomAD AF^b^	0 alleles	29/28,198 alleles	5/259550 alleles		
CADD^c^	27	34	32		
Clinical presentation					
Symptom Onset	Congenital: reduced fetal movements, hypotonia at birth		Congenital: hypotonia and tachypnea at birth	Congenital: hypotonia at birth	
Neuromuscular	Severe and progressive weakness		Severe and progressive weakness, right‐sided brachial plexus palsy	Severe and progressive weakness	
Respiratory	Respiratory insufficiency requiring nighttime non‐invasive ventilation (infancy) followed by ventilation via tracheostomy (4 years)		Respiratory failure (4 months)	Respiratory insufficiency (4 months)	
Gastrointestinal	Feeding difficulties requiring gastrostomy tube		Feeding difficulties requiring gastrostomy tube	Feeding difficulties requiring gastrostomy tube	
Ophthalmologic	Restricted extraocular movements, high myopia, bilateral cataracts		–	Poor tracking	
Neurologic	Delayed speech with poor articulation, progressive, and refractory epilepsy (4 years)		–	–	
CK (U/l)	3,667–6,807		436–2,361	921–4,000s	
Muscle Biopsy	Dystrophic. Reduced glycosylation of α‐dystroglycan (10 months)		NP	Myopathic	
Electromyography	Myopathic (9 months)		Normal (3 weeks)	NP	
Brain MRI	Normal (7 months), mild increase in T2‐ weighted white matter signal (28 months)		Normal (2 weeks)	Normal (4 days)	
Bronchoscopy	NP		Mild tracheomalacia and mild bronchomalacia (4 months)	Severe glossoptosis, bronchomalacia, and oral secretions (4 months)	
Echocardiogram	Normal		Normal	NP	

NP, not performed.

^a^ Transcript ID: NM_005868.6.

^b^ Genome aggregate database allele frequency.

^c^ Combined Annotation Dependent Depletion.

Trio ES in P2 revealed one homozygous missense variant (c.152T>G; p.(Ile51Ser)) in *BET1* (NM_005868.6). The p.(Ile51Ser) variant is rare, predicted to be damaging (MutationTaster: disease causing; CADD Prediction: 32), and affects a highly conserved residue (Fig 2A). This variant has been reported five times in the heterozygous state in the gnomAD database with the popmax filtering allele frequency of 0.00127% (highest population frequency of 0.004%, corresponding to 5/122910 alleles in the European non‐Finish population in gnomAD v.2.1.1, no homozygous individuals). Exome sequencing was initially unrevealing in P3, but upon reanalysis after the birth of his affected sister, the biallelic *BET1* p.(Ile51Ser) variant was identified in him as well.

### *BET1* c.202G>C variant acts as a complex hypomorph splice variant

To evaluate the effect of the biallelic *BET1* variants identified, we pursued RNA sequencing (RNA‐seq) in P1’s muscle biopsy. Based on the coverage of exons 1–4, the expression level of the BET1 locus in muscle tissue from P1 was comparable to that from control samples (Fig 2B), suggesting that the overall BET1 transcripts in P1 do not undergo NMD. Interestingly, two abnormal splicing patterns were observed affecting the junction between exons 3 and 4 in P1 (Fig 2B). In one aberrant transcript, there was a complete skipping of exon 4 (NM_005868.6), which corresponded to the exon splicing pattern of a non‐coding transcript (NR_133909.2). The second abnormal splicing event at this site consisted of skipping of the first 34nt of exon 4 due to unmasking of a cryptic splice acceptor site. This exact splice acceptor gain, secondary to the c.202G>C variant, was predicted by SpliceAI although scoring below the threshold. The same two abnormal splicing patterns were also observed in the RNAseq data from patient’s cultured fibroblasts (Fig EV1B). None of these splicing events were observed in RNAseq data from control muscle biopsies. Detailed analysis of the sequencing reads corresponding to these abnormal transcripts revealed that they were derived from the paternal allele carrying the c.202G>C variant, as absence of the maternal c.134delC variant was confirmed in 14 reads. This was consistent in both muscle and fibroblast RNAseq data. Finally, we observed normally spliced reads with the c.202G>C variant resulting in a full‐length Asp68His BET1 protein. Thus, the c.202G>C variant led to a partial abolishment of the natural splice acceptor site, producing three different splice forms: exon 4 skipping, 34nt deletion due to the activation of a downstream cryptic splice acceptor site, and a normally spliced transcript (Fig 2C).

To determine whether there was an allele imbalance of BET1 expression in muscle tissue from P1, we counted the number of maternally and paternally derived muscle RNAseq reads covering each of the two BET1 variants (c.134delC in exon 2 and c.202G>C in exon 4). No conclusion could be drawn from the exon 2 site since only nine reads were observed at that position, all from the paternal allele without the c.134delC variant. The exon 4 site was covered with 23 reads, corresponding to 13 maternally derived reads (normally spliced reads without the c.202G>C variant) and 10 paternally derived reads (two normally spliced variants with c.202G>C variant, reads skipping exon 4 and two abnormally spliced reads missing first 34nt of the exon 4). Thus, there seem to be no allelic imbalance in the expression of BET1 in the muscle tissue from P1. Both aberrantly spliced transcripts and the transcript with the frameshift variant are expected to produce a truncated and likely unstable protein. Thus, the only full‐length protein product expected to be present in P1’s muscle would derive from the few normally spliced paternal alleles carrying the (c.202G>C; p.(Asp68His)) variant. Based on the RNAseq data (2 out of 23 reads), we expect this mutated Asp68His BET1 protein to be present at approximately 9% of normal protein level.

### BET1 expression levels and localizations in P1 and P2 fibroblasts

**Figure 3:**
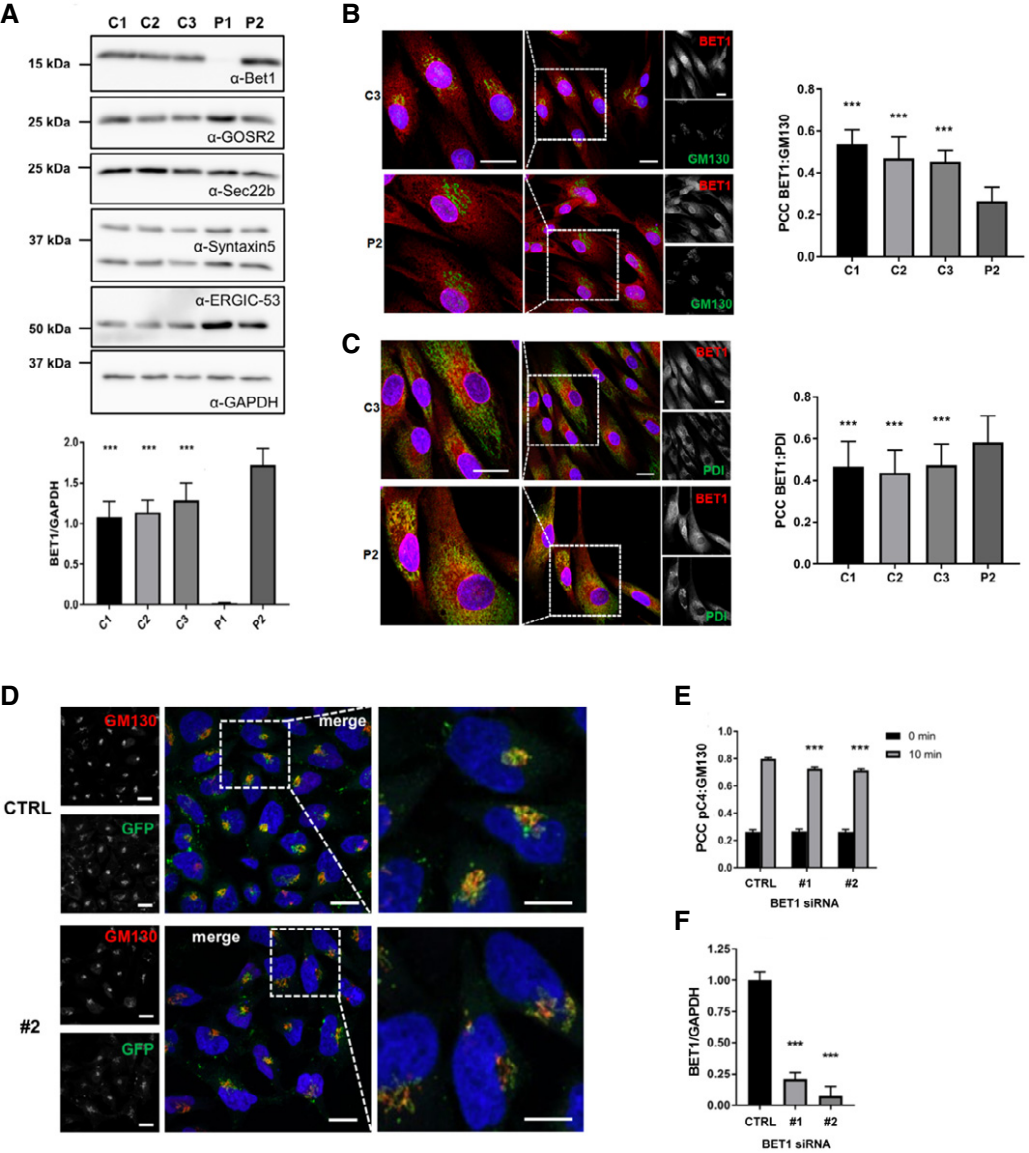
Analysis of BET1 expression and localization in patient fibroblasts and ER‐to‐Golgi transport in *BET1* knockdown HeLa cells Immunoblot of patient fibroblast cell lines (P1 and P2) and three independent controls (C1‐3). Immunoblots were probed with the indicated primary antibodies. Quantification of BET1 protein levels normalized to GAPDH. Data represented are mean ± SD of four independent biological experiments. Significant differences of P1 BET1‐levels were checked compared to each control (***P < 0.001, one‐way‐ANOVA followed by Tukey’s *post hoc* test).Subcellular localization of BET1 in control and patient fibroblasts by indirect immunofluorescent confocal microscopy. Representative immunofluorescence images of BET1 (red) and *cis‐*Golgi marker GM130 (green). Scale bar: 20 µm. Colocalization of BET1 with GM130 was determined using Pearson’s correlation coefficient (PCC) measured per cell (right panel; C1: *n* = 63, C2: *n* = 50, C3: *n* = 49 and P2: *n* = 33) (***P ≤ 0.001, one‐way ANOVA was followed by Tukey’s *post hoc* test). Means are shown ± SEM.Representative immunofluorescence images of BET1 (red) and ER marker PDI (green). Scale bar: 20 µm. Right panel shows PCCs for BET1 versus PDI (C1: *n* = 61, C2: *n* = 42, C3: *n* = 59 and P2: *n* = 32 of two biological replicates). (***P ≤ 0.001, one‐way ANOVA was followed by Tukey’s *post hoc* test). Means are shown ± SEM.HeLa cells stably expressing the pC4‐reporter were transfected with control siRNA (ctrl) or two independent siRNAs (#1 and #2) directed against *BET1* (siRNA #1 is shown in figure EV 2B). 2d post‐transfection the pC4‐secretion assay was performed for 0 and 10 min post‐induction of secretion. Representative images are shown (upper panel ctrl and lower panel #2 siRNA, respectively. Scale bar: 20 µm. Right panels represent magnifications of dashed areas.Quantification of colocalization using the Pearson's correlation coefficient (PCC) of the pC4‐construct (GFP) and the *cis*‐Golgi marker GM130 (0 min: ctrl *n* = 63, #1 *n* = 56, #2 *n* = 64; 10 min: ctrl *n* = 104, #1 *n* = 126, #2 *n* = 110). Shown are means ± SEM. Colocalization is significantly reduced by *BET1* knockdown (siRNA #1 and #2) compared to control transfected cells. Significant differences of PCC (pC4:GM130) after 10 min induction with solubilizer were analyzed with a one‐way‐ANOVA followed by Dunnett's *post hoc* test. ***P ≤ 0.001. 0 min: ctrl 0.265 ± 0.016, #1 0.268 ± 0.017, #2 0.264 ± 0.018; 10 min: ctrl 0.798 ± 0.009, #1 0.726 ± 0.011, #2 0.715 ± 0.011.Quantification of knockdown efficiency of *BET1* in HeLa‐pC4 cells by immunoblots. Data represented are mean ± SD of three biological replicates. ***P < 0.001 compared to ctrl cells, one‐way‐ANOVA followed by Dunnett’s *post hoc* test). Source data are available online for this figure.

**Figure EV2:**
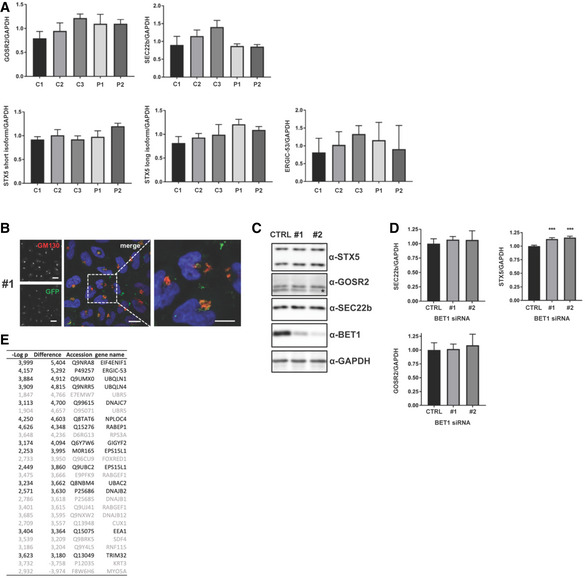
Normal SNARE complex partners in BET1 fibroblasts with reduced colocalization at the *cis*‐Golgi in transfected cells Quantification of protein levels of BET1 SNARE complex partners and the novel interaction partner ERGIC‐53 in fibroblasts. Values are normalized to GAPDH. No significant differences between the control and patient fibroblasts were detected. Data represented are mean ± SD of four biological replicates. Significant differences were analyzed by one‐way ANOVA followed by Tukey's *post hoc* test.Immunofluorescence images of *BET1* knockdown HeLa pC4 cells transfected with BET1 siRNA #1 10 min after induction of ER‐to‐Golgi transport with solubilizer. Scale bar: 20 µm.HeLa pC4 cells were transfected with control siRNA (ctrl) or siRNA against *BET1* (#1 and #2) and protein levels of BET1 and ER‐to‐Golgi complex SNAREs SEC22b, Syntaxin‐5, and GOSR2 were analyzed by immunoblot. *residual SEC22b signal after immunoblot stripping.Quantification of EV2C. Data represented are mean ± SD of three biological replicates. Significant differences to ctrl cells were analyzed by one‐way ANOVA followed by Dunnett’s *post hoc* test. ***P ≤ 0.001.Table showing a spectral count‐based summary of three independent AP‐MS experiments with significant hits shown in Fig 5A. Hits are sorted by the difference of BET1‐WT and BET1‐Ile51Ser. Hits which do not fulfill the criteria to have a mock‐transfected spectral count of two or less are colored in grey.

We proceeded to analyze the impact of the identified variants on BET1 protein expression and localization in P1 and P2’s fibroblasts. Overall BET1 levels in P2 were comparable to controls; while for P1, we found a strong reduction in expression (Fig 3A), which was consistent with the RNA findings for these two individuals. Levels of SNARE‐complex partners GOSR2, SEC22b, and Syntaxin‐5 were normal in patient’s cells compared to controls (Figs 3A and EV2A).

We subsequently analyzed the subcellular localization of BET1 in P2’s fibroblasts in which it is detectable, and found a decreased colocalization of BET1 with *cis‐*Golgi marker GM130 and an increased colocalization with ER marker PDI (Fig 3B and C). This is in line with the reduced colocalization of BET1 with GOSR2 and Syntaxin‐5, while colocalization of BET1 with SEC22b was normal (Fig EV1C). This suggests that localization of mutant Ile51Ser BET1 is shifted from the Golgi to the ER in P2 fibroblasts. We could not further analyze P1 fibroblasts for BET1 localization by immunofluorescence due to the very faint BET1 signal caused by the hypomorphic nature of the disease alleles in P1.

### Knockdown of *BET1* impairs ER‐to‐Golgi trafficking in cells

Given our findings that BET1 transcript and expression levels are down in P1‐derived fibroblasts, we aimed to demonstrate the impact of reduced BET1 expression on ER‐to‐Golgi trafficking. Therefore, we modified the iDimerize Reverse Dimerization System, which has been used to identify the role of SNAREs in constitutive secretion in mammalian cells (Gordon *et al*, 2010). Briefly, this assay utilizes an eGFP‐tagged reporter construct (S_1_‐eGFP‐FM_4_‐FCS‐hGH) containing multiple mutant FKBP domains that form ER‐retained aggregates. These aggregates can be solubilized by the FKBP ligand AP21998 (a rapamycin analog) leading to efficient and rapid trafficking of the reporter along the secretory pathway. To analyze the effect of *BET1* knockdown on ER‐to‐Golgi transport, we used HeLa‐M (C1) cells expressing the reporter (Gordon *et al*, 2010) and transiently transfected them with two independent siRNAs (transfection of scrambled siRNA served as controls). Immunoblotting of cellular lysates confirmed efficient knockdown of BET1 with no change in expression levels of SEC22b and GOSR2 and a marginal but significant increase of total Syntaxin‐5 levels (Figs 3F and EV2C and D). Next, we added solubilizer to control and siRNA‐treated HeLa cells for 10 min and monitored the colocalization of the reporter by GFP fluorescence and the Golgi marker GM130 by Pearson correlation coefficient (PCC). Whereas we could not detect any colocalization of the reporter with GM130 under basal conditions (0 min), we found significantly less colocalization of the reporter with GM130 in HeLa cells treated with siRNA against BET1 after 10 min of addition of the solubilizer (Figs 3D and E, and EV2B; scrambled siRNA treated cells served again as controls). These findings indicate that *BET1* knockdown impacts ER‐to‐Golgi transport.

### Missense *BET1* variants impact conserved domains of the SNARE complex

**Figure 4:**
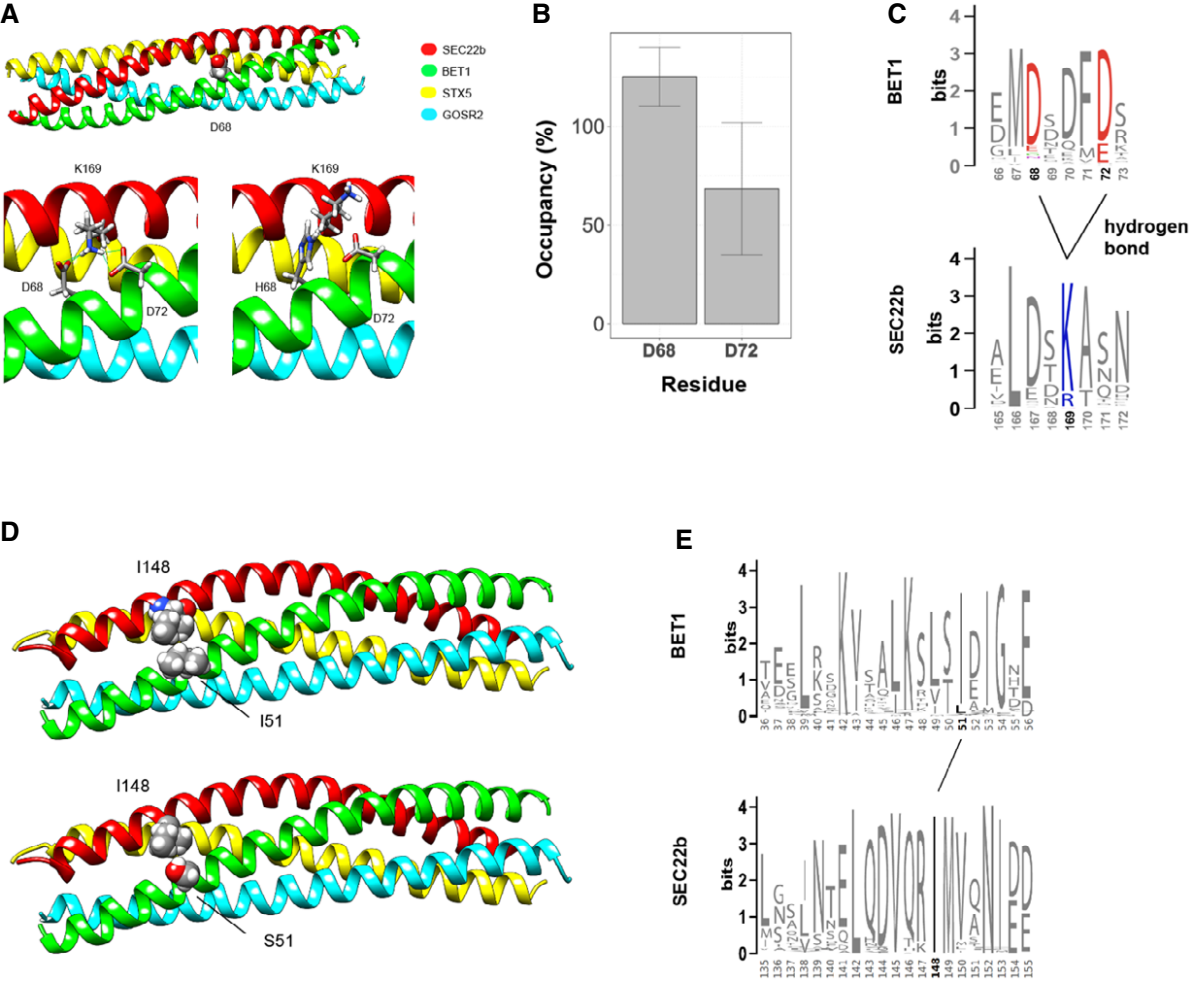
*In silico* characterization of the SNARE complexes bearing the Asp68His and the Ile51Ser variant Representation of the SNARE complex with Asp68 highlighted (top). BET1^Asp68^ is involved in a hydrogen bond with SEC22b^Lys169^, with the most probable conformation of His68 in the mutant variant (bottom).Stability of hydrogen bonds between BET1^Asp68^ and BET1^Asp72^ respectively was estimated using molecular dynamics simulation in three replicas. Parameter occupancy could be more than 100% if several stable hydrogen bonds could be established by residue. Bars represent average fraction of the simulated time the residue is involved in a hydrogen bond(s). Data represented are mean ± SD.Sequence conservation of the corresponding regions of BET1 and SEC22b animal proteins. The relevant residues involved in hydrogen bond network are highlighted by color, hydrogen bonds are shown.Representation of the SNARE complex with Ile51 (upper) and Ser51 (lower) residues highlighted.Sequence conservation of the corresponding regions of BET1 and SEC22b and the relevant residues involved in the hydrophobic patch formed by Ile51 BET and SEC22b^Ile148^.

Since the identified *BET1* missense variants are located within the conserved SNARE domain (Fig 2A), we wanted to better understand their effect on the SNARE complex and modeled the human ER‐to‐Golgi SNARE complex using available structures as templates (Volker *et al*, 2017). BET1 p.(Asp68His) was included in these analyses, as in addition to its hypomorph expression, the RNA sequencing data predicted that all residual full‐length BET1 protein that is generated contains Asp68His. In this modeling, we found the side chain of BET1 Asp68 pointing toward the interior of the complex (Fig 4A), which could indicate that this residue participates in interactions crucial for the stability and function of the SNARE complex. We explored this hypothesis using molecular dynamic (MD) simulations of the ER‐to‐Golgi SNARE complex consisting of wild‐type versions of the SNARE domains of Syntaxin‐5 (Q_a_‐SNARE), GOSR2 (Q_b_‐SNARE), BET1 (Q_c_‐SNARE), and SEC22b (R‐SNARE). MD simulations revealed that Asp68 is involved in a highly stable hydrogen bond with Lys169 of SEC22b of the neighboring helix of the four‐helix bundle complex (Fig 4A–C) and that this bond is further stabilized by another stable hydrogen bond between Lys169 of SEC22b and Asp72 of BET1 (Fig 4C). This creates a hydrogen bond network that appears to be highly conserved across animal orthologs; however, its exact function is yet to be determined (Fig 4C). The change of Asp68 to His68, which side chain contains an imidazole ring that limits its rotational freedom, is predicted to impact Lys169 of SEC22b and disrupt the hydrogen bond network (Fig 4A). Therefore, it is conceivable that the p.(Asp68His) variant might interfere with the function of the SNARE complex.

Modeling for the BET1 Ile51 residue, on the other hand, indicates that the side chain of this residue is localized at the outer surface of the SNARE complex in close proximity to a highly conserved hydrophobic residue (Ile148) in SEC22b (Fig 4D and E). These two residues are likely to form a hydrophobic patch on the outer surface of the SNARE complex, which might interfere with interaction partners of BET1 rather than participating in SNARE complex stability and function.

### Ile51Ser BET1 affects interaction with novel partner ERGIC‐53 but not with SNARE‐complex members

**Figure 5:**
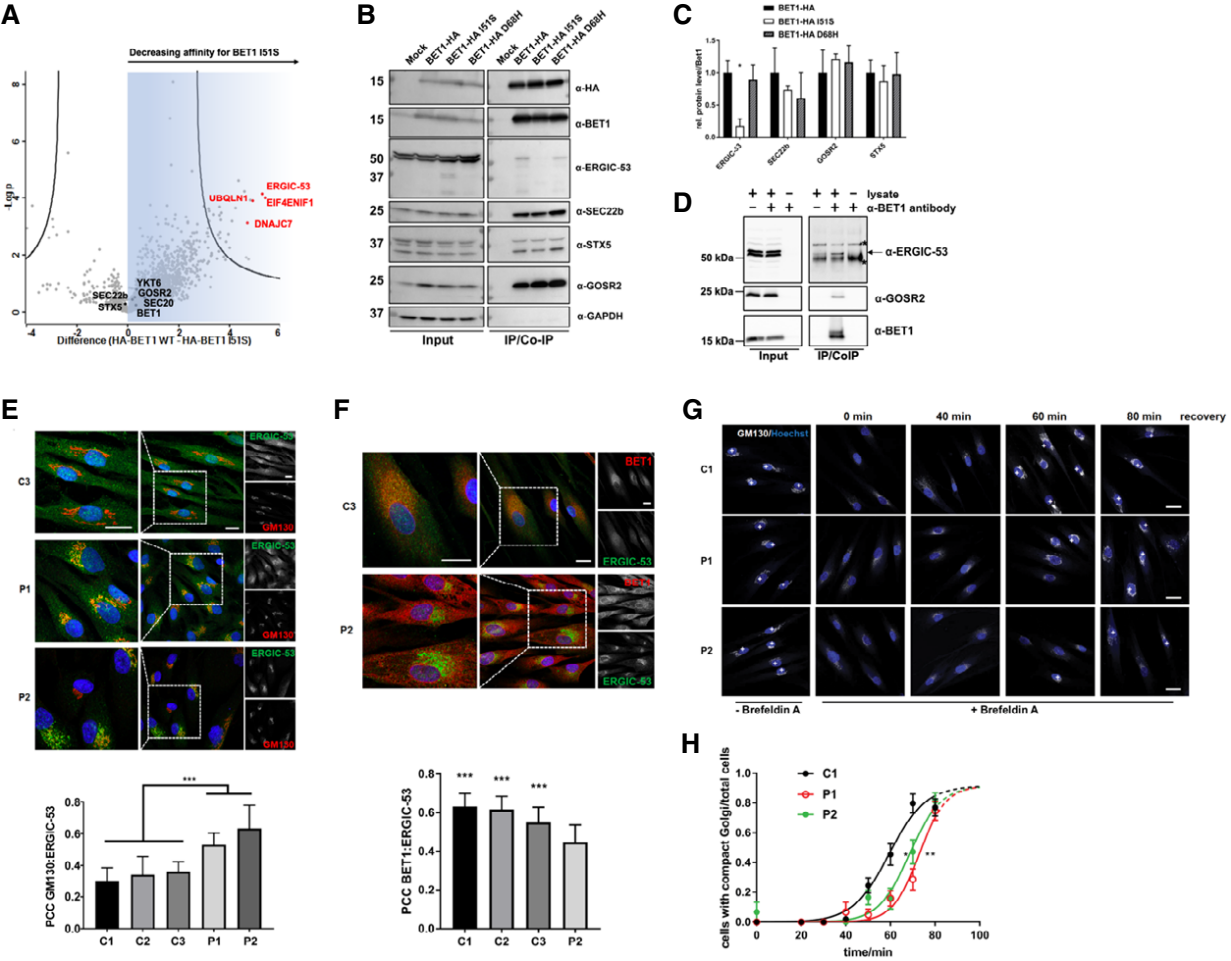
ERGIC‐53 is a novel interaction partner of BET1 with impaired interaction of Ile51Ser BET1 and ERGIC‐53 Volcano plot of AP‐MS data using HA‐BET1 or Ile51Ser.Co‐IPs using HA‐tagged constructs expressed in HEK293 cells. As baits HA‐tagged proteins were immunoprecipitated with anti‐HA beads. IP samples and cell lysates were subjected to immunoblotting with α‐HA, α‐ERGIC‐53, α‐BET1, α‐SEC22b, α‐GOSR2, and α‐GAPDH antibodies, respectively.Quantification of three independent replicates of Co‐IP experiments. Protein levels of the Co‐IP samples were normalized to BET1‐HA‐levels. Data represented are mean ± SD of three biological replicates. Significant differences were checked by one‐way ANOVA followed with Tukey's post hoc test. *P ≤ 0.05.Endogenous Co‐IP of BET1 and ERGIC‐53. Immunoblots of HEK293T cell lysates (+lysate) or lysis buffer (−lysate) incubated with (+) or without (−) BET1 antibody followed by precipitation with protein A/G beads. ERGIC‐53 signal was only present in samples with lysate and antibody, whereas * denotes unspecific signals from beads used for precipitation. As a positive control, GOSR2 antibody was used.Subcellular localization of ERGIC‐53 in fibroblasts by indirect immunofluorescent confocal microscopy. Representative immunofluorescence images of ERGIC‐53 (green) and GM130 (red) in fibroblasts from P1, P2, and a control cell line. Scale bar: 20 µm. PCC (right panel) for GM130 versus ERGIC‐53 (C1: *n* = 74, C2: *n* = 95, C3: *n* = 59, P1: *n* = 72 and P2: *n* = 76). (***P ≤ 0.001, one‐way ANOVA was followed by Tukey’s *post hoc* test). Shown are means ± SEM. Immunofluorescence images shown are one example of a total of two biological replicates.Colocalization of ERGIC‐53 and BET1 in control and P2‐derived fibroblasts. Representative immunofluorescence images of ERGIC‐53 (green) and BET1 (red) in fibroblasts from P2 and a control cell line. Scale bar: 20 µm. PCC (right panel) for BET1 versus ERGIC‐53 (C1: *n* = 72, C2: *n* = 46, C3: *n* = 56 and P2: *n* = 37). (***P ≤ 0.001, one‐way ANOVA was followed by Tukey’s *post hoc* test). Data represented are mean ± SEM of two independent experiments.Immunofluorescence images of control fibroblasts (C1) and patient fibroblasts (P1 and P2) treated with Brefeldin A for 40 min and Golgi recovery for indicated time points. Golgi reconstitution was analyzed by immunofluorescence microscopy of the *cis*‐Golgi marker GM130. Cells displaying an intact Golgi are marked with a plus (+). Scale bar: 20 µm.The number of cells with an intact Golgi was divided by the number of total cells per image and plotted against time (three biological replicates). Dots are means ± SD. For quantification, a sigmoidal fit was performed with the software GraphPad Prism 7. Dashed lines indicate extrapolated plateaus. LogIC50 ± 95% confidence interval: C1 = 59.38 ± 2.31 min, P1 = 72.89 ± 1.65 min, P2 = 68.57 ± 1.929 min. Significant differences were assumed when 95% confidence interval do not overlap. Source data are available online for this figure.

To identify interacting partners that could be impacted by the p.(Ile51Ser) missense, we performed affinity purification coupled to mass spectrometry (AP‐MS) in HEK293T cells transiently transfected with HA‐tagged wild‐type BET1 or BET1 Ile51Ser. Among the most abundant interactors was the SNARE complex member GOSR2 in addition to other SNAREs (e.g., YKT6 and SEC20 also involved in membrane trafficking between the ER, ERGIC, and the Golgi) (Figs 5A and EV2E, and Table EV1). Importantly, interaction of these SNAREs was not reduced in Ile51Ser BET1 (Fig 5A and Table EV1). Among proteins that did not co‐precipitate with the Ile51Ser mutant, we found the eukaryotic translation initiation factor 4E transporter (EIF4ENIF1), Ubiquilin 1 (UBQLN1), the chaperone DNAJC7, and ERGIC marker protein ERGIC‐53.

Next, we wanted to corroborate the AP‐MS results and focused on one of the top candidates, ERGIC‐53 since this protein resides in the same subcellular compartment as BET1 where it serves as a mannose‐specific membrane lectin as a cargo receptor transporting glycoproteins from the ER to ERGIC (Schindler *et al*, 1993). Co‐immunoprecipitations confirmed that the BET1 Ile51Ser variant was unable to precipitate ERGIC‐53, whereas BET1 Asp68His pulled down ERGIC‐53 as efficiently as wild‐type BET1 (Fig 5B and C). Importantly, the BET1 and ERGIC‐53 interaction was also confirmed by endogenous co‐immunoprecipitations (Fig 5D). We also verified that the ER‐to‐Golgi SNARE complex partners, GOSR2 and Syntaxin‐5, were co‐precipitated by both BET1 wild‐type and the two mutated BET1 proteins in the same order of magnitude. Therefore, the interaction with SNARE complex partners is not directly affected by the missense variants (Fig 5B and C). Interestingly, only SEC22b was less co‐precipitated—albeit not significantly—by the two variants, which might be in line with the MD simulations that Asp68 is involved in forming a hydrogen bond network with SEC22b (Fig 4A–C). To further characterize our findings that BET1 and ERGIC‐53 are novel interaction partners and that the p.(Ile51Ser) variant abolishes this interaction, we re‐analyzed the patient fibroblast cell lines by immunoblotting and immunofluorescence. We found in P1 and P2 fibroblasts that the overall ERGIC‐53 levels were unchanged compared to control fibroblasts (Figs 3A and EV2A); however, there was increased colocalization of ERGIC‐53 with the *cis*‐Golgi marker GM130 compared to control (Fig 5E). In line with these findings, we also demonstrated significantly reduced colocalization of ERGIC‐53 and mutated BET1 in fibroblasts from P2 in which mutant BET1 protein can be directly assessed (Fig 5F). These results suggest that altered localization of ERGIC‐53 is a characteristic trait in fibroblasts of patients with BET1‐related disease, which appears to be independent of the underlying pathogenic *BET1* variant.

### The effect of BET1 variants on yeast growth

**Figure EV3:**
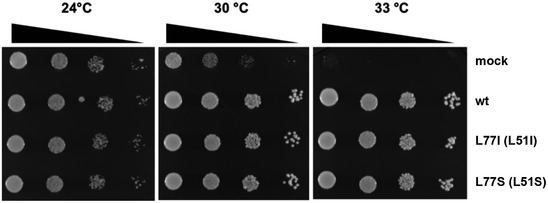
The effect of BET1 variants on yeast growth BET1 Leu77Ile (Leu51Ile) and Leu77Ser (Leu51Ser) show similar growths compared to BET1 wild type. Temperature‐sensitive strain of *S. cerevisiae* was transformed with wild‐type BET1, the two different variants, and a mock plasmid. At 24°C, growth was detected for all variants and wild type, including the negative control. Only the negative control showed an impairment of growth at restrictive temperatures (30°C and 33°C).

Given our findings that ER‐to‐Golgi SNARE complex member interaction is not impaired in the two BET1 variants, we wanted to further test their pathogenic impact in yeast. This model is well suited as all members of the ER‐to‐Golgi SNARE complex are evolutionary‐conserved and a temperature‐sensitive bet1 mutant strain is available (Boehm *et al*, 1994), which grows at permissive but not restrictive temperatures (Fig EV3). The bet1 strain failed to thrive at restrictive temperatures and could be rescued by introduction of wild type scbet1 and also by sc*bet1* carrying the orthologous (p. Leu77Ser) mutation. Since *scbet1* has a leucine instead of an isoleucine at the corresponding position 77, we also included sc*bet1* Leu77Ile in this analysis to demonstrate that these residues can be substituted without affecting the ability of *scbet1* to rescue growth at restrictive temperatures (Fig EV3). We excluded the BET1 Asp68His variant from this analysis because of a lack of evolutionary conservation of the corresponding residue at this position (glycine in yeast and aspartate in human) (Fig 2A). In summary, these data strongly suggest that the BET1 p.(Ile51Ser) residue does not interfere with SNARE complex assembly nor function in yeast.

### Golgi‐reconstitution is slowed in patient fibroblasts

To analyze the impact of the BET1 variants on Golgi structure/function in more detail, we performed a Golgi reconstitution assay by using the fungal metabolite brefeldin A (BFA). BFA inhibits the ADP‐ribosylation factor ARF1 and causes the reversible Golgi break down into vesiculated Golgi membranes (Chardin & McCormick, 1999). To monitor *cis*‐Golgi morphology, we checked the localization of the Golgi marker protein GM130 by immunofluorescence. Incubation of control and patient fibroblasts with BFA resulted in a dispersed GM130 signal (Figure 5G, 0 min recovery), whereas the GM130 signal in untreated cells localized to perinuclear regions, which was reminiscent of a morphological intact Golgi apparatus. In control cells 60 min after BFA removal, GM130 localization was in roughly half of cells comparable to untreated cells. In both patient cell lines, the GM130 signal was at this time point more dispersed and less focused in the perinuclear regions (Figure 5G and H), suggesting that Golgi reconstitution was significantly slowed in fibroblasts from patients with BET1‐related disease.

## Discussion

We report detailed clinical data on three individuals of two independent families with biallelic variants in *BET1,* clinically manifesting as a severe and progressive early‐onset dystrophy with epilepsy in one patient. This newly identified disease gene, *BET1*, can be added to the list of inherited disorders of trafficking (IDT) and CMDs. This rapidly expanding clinically heterogenous group of disorders manifests with varying degrees of hypopigmentation, disturbed cell‐mediated immune system, and neurological abnormalities (Gissen & Maher, 2007; De Matteis & Luini, 2011). Congenital muscular dystrophies are emerging as a rare clinical manifestation of IDT and have only recently been reported in individuals with progressive muscle weakness and seizures, whom were found to have recessive variants in *TRAPPC11* and in rare patients with *GOSR2* variants (Larson *et al*, 2018; Henige *et al*, 2021; Stemmerik *et al*, 2021). Pathogenic variants in *GOSR2* were first described to cause progressive myoclonus epilepsy and ataxia with rare concomitant manifestation of CMD (Corbett *et al*, 2011; Boisse Lomax *et al*, 2013). It is noteworthy though that a subset of individuals with *GOSR2* pathogenic variants have been noted to have a mildly elevated serum CK (median 734 U/l) in the setting of normal muscle histology and no reported muscle involvement clinically (Boisse Lomax *et al*, 2013). The rare individuals with pathogenic variants in *TRAPPC11* and *GOSR2* who manifested with a muscular dystrophy with overt muscle weakness and more highly elevated CK, similar to our individual (P1) with BET1‐deficiency, importantly also showed abnormal immunofluorescence and Western blotting indicative of hypoglycosylation of α‐dystroglycan (α‐DG) using an antibody against the specific glycoepitope of α‐DG (Larson *et al*, 2018; Stemmerik *et al*, 2021). Hypoglycosylation of α‐DG is a known disease‐causing mechanism for a genetically heterogeneous CMD subgroup known as dystroglycanopathies that clinically manifests as a muscular dystrophy with variable central nervous system involvement (Godfrey *et al*, 2011; Nickolls & Bonnemann, 2018). Dystroglycan is a dystrophin‐associated cell surface glycoprotein and is essential for providing transmembrane linkage between the extracellular matrix and cytoskeleton. A specialized O‐mannosyl‐linked glycosylation of dystroglycan resulting in “matriglycan” is an essential post‐translational modification needed to allow for binding of extracellular matrix proteins (Barresi & Campbell, 2006). The α‐DG clinical severity spectrum includes CMD with profound structural brain malformations (Walker–Warburg syndrome, muscle–eye–brain (MEB) disease, and Fukuyama congenital muscular dystrophy) to muscular dystrophy with normal appearing brain imaging but significant cognitive impairment and further extends to adult‐onset limb girdle muscular dystrophy without CNS involvement (Godfrey *et al*, 2011). Our affected individuals’ clinical presentations were consistent with an early onset progressive muscular dystrophy, while in P1 the refractory epilepsy which developed was unusually progressive and severe for even an α–dystroglycanopathy with significant brain involvement. It is notable that brain imaging did not demonstrate structural brain malformations, as often seen in individuals with dystroglycanopathy with epilepsy. It is possible that several types of glycosylation, including N‐ and O‐linked, might be impaired in BET1‐related disease as they all require proper ER‐to‐Golgi transport. Given that many cellular functions rely on vesicle transport, we suspect that in addition to impaired glycosylation of α‐dystroglycan there may be additional disease mechanisms at play in BET1‐related disease. Therefore, dysregulation of α‐dystroglycan transport to the plasma membrane is certainly a major consequence of BET1 absence and/or dysfunction and a likely contributor to the CMD. However, the full extent of muscular dystrophy and brain involvement in individuals with *BET1* variants may be related to additional, cell‐specific alterations in protein trafficking that remain to be elucidated.

We propose that the *BET1* variants identified here impact Q‐SNARE‐mediated vesicle transport through reduction in overall BET1 levels (p.(Ala45Valfs*2) and c.202G>C), or specifically impaired interactions with binding partner such as the transmembrane cargo receptor ERGIC‐53 (p.(Ile51Ser)). Interestingly, mutant BET1 shows normal interaction with SNARE complex partners (Fig 5A) and does not impact SNARE complex function in temperature‐sensitive yeast experiments (Fig EV3). In contrast, we demonstrate impaired interaction between mutant BET1 and ERGIC‐53, making ERGIC‐53 an emerging player in SNARE‐mediated transport with a potential new role in muscle homeostasis and disease. Furthermore, BET1 appears to be important for normal ERGIC‐53 localization and likely function. These results indicate a role of BET1 beyond SNARE complex formation although further studies in BET1 pathomechanism and the role of ERGIC‐53 in CMDs are needed as more patients and disease‐causing variants are found in the future.

The shift in localization of mutant BET1 away from the *cis*‐Golgi and toward the ER in patients’ cells (Figs 3B and C, and EV1C) suggests altered ER‐to‐Golgi trafficking as a potential pathomechanism for this new disease. The detrimental effect on vesicular trafficking was subsequently confirmed through downregulation of *BET1* by siRNAs resulting in impaired ER‐to‐Golgi transport (Fig 3D). This assay mimics the BET1 deficiency noted in Patient 1, while the precise effect of the homozygous missense p.(Ile51Ser) BET1 variant is less well modeled in this system. Overall, our functional analysis suggests that in this case impaired binding of BET1 to novel interaction partners may result in similar loss or diminished function. In this study, we were able to identify novel interaction partners for BET1 and showed for one of our top hits, ERGIC‐53, a specifically impaired interaction with the p.(Ile51Ser) variant form of BET1. The additional partners we identified are of interest as well, but further studies are needed to elucidate their role in vesicular trafficking. For example, Ubiquilin 1 (UBQLN1) expression is associated with neuronal loss seen in Alzheimer’s disease, which is of interest as cognitive decline was observed in at least one patient with BET1‐related disease (Stieren *et al*, 2011). An additional notable partner is DNAJC7, a chaperone and regulator of protein homeostasis that has recently been identified as a genetic cause for amyotrophic lateral sclerosis (Farhan *et al*, 2019).

In summary, we report three individuals from two families with biallelic variants in the Qc‐SNARE gene *BET1* presenting with a multisystem neurodegenerative disease that manifests with congenital onset and severely progressive muscular dystrophy, as well as the development of cataracts and refractory epilepsy in one. Highly elevated CK levels, dystrophic muscle biopsy findings with evidence of hypoglycosylation of α‐dystroglycan, cataracts, and involvement of the central nervous system are reminiscent of the CMD subtype of α–dystroglycanopathy. We found reduced and impaired localization of mutant BET1, and we identified ERGIC‐53 as a novel BET1 interaction partner that is specifically impaired in its interaction by the presence of the p.(Ile51Ser) BET1 variant. Furthermore, we support the pathogenicity of the *BET1* variants by demonstrating impaired vesicle transport between ER and Golgi. Taken together, we establish *BET1* as a novel CMD/epilepsy gene and confirm the emerging role of ER‐to‐Golgi Q‐SNARE gene variants in CMD with epilepsy. The data from individuals with BET1‐related disease further confirm that impaired SNARE‐mediated Golgi trafficking of α‐dystroglycan may contribute to this severe phenotype and highlights its relationship to α‐DG‐related CMD with CNS involvement (i.e., IDT‐related dystroglycanopathies). Abnormal processing of other, yet to be identified, proteins may well contribute to the phenotype as well. Considering *BET1* variants in individuals with epilepsy and/or CMD of unknown genetic origin may identify more individuals and thus provide further insight into the clinical manifestations and pathomechanism of BET1‐related disease. We suspect that the complete clinical spectrum of *BET1* variants will be more fully characterized with the identification of additional affected individuals, and similarly to *GOSR2*, the phenotype may be a spectrum, ranging from a predominant epilepsy syndrome at one end of the spectrum to an α‐DG‐related CMD with refractory epilepsy at the other, more severe end of the spectrum.

## Materials and Methods

### Recruitment and sample collection

Individuals were identified through their local neurology and/or genetics clinic. Informed consent for study procedures and photographs was obtained by a qualified investigator (protocol 12‐N‐0095 approved by the National Institute of Neurological Disorders and Stroke, National Institutes of Health). Experiments conformed to the principles set out in the WMA Declaration of Helsinki and the Department of Health and Human Services Belmont Report. Medical history was obtained, and clinical evaluation, biopsies, and MRI imaging were performed as part of the standard neurologic evaluation. DNA, muscle, and skin biopsy samples were obtained according to standard procedures.

### Exome, genome, and RNA sequencing

Trio exome sequencing for P1 was performed through the NIH Intramural Sequencing Center using the NimblegenSeqCap EZ Exome + UTR Library and Illumina HiSeq 2500 sequencing instruments. Variants were analyzed with Varsifter and *seqr* and searched for in dbSNP, the National Heart, Lung and Blood Institute Exome Variant Server and the Genome Aggregation database (GnomAD; Teer *et al*, 2012; Karczewski *et al*, 2020). Trio PCR‐free genome sequencing was pursued at Broad and included the following: sample identification QC (fingerprinting), sample preparation utilizing custom Broad indices (IDT) and Kapa Biosciences HyperPrep library construction kit, sequencing (Illumina 2 × 150 bp reads), read demultiplexing, aggregation, and alignment (BWA‐MEM). Tru‐Seq Non‐Strand Specific RNA Sequencing (High Coverage (50 M pairs)) on RNA extracted from muscle and fibroblasts was pursued at Broad using a non‐strand specific Illumina TruSeq Protocol and sequence coverage to 50 M Paired reads or 50 M Total reads (± 5%). The *BET1* variants were confirmed by Sanger sequencing (Genewiz) in P1 and his parents. Quartet ES for P2 and P3 was performed as previously described (Retterer *et al*, 2016). To verify the presence of the c.134delC and c.202G>C *BET1* variants, a 373‐bp product was amplified using the forward primer 5′‐ CTC CTG GCAACT ATG GGA AC−3′ and the reverse primer 5′‐ TGCAAGCCATTAAGTTGGAA−3′. Amplification was performed with 35 cycles using denaturation at 95°C for 30 s, the annealing temperature at 58°C for 30 s and elongation at 72°C for 1 min.

### Antibodies and plasmids

The following antibodies were used: anti‐BET1 (SC‐136390, Santa Cruz), anti‐ERGIC‐53 (13364‐1‐AP, Proteintech) or (EPR6979, Abcam), anti‐GOSR2 (170003, Synaptic Systems), anti‐HA (12CA5, Roche), anti‐GAPDH (CB1001, Millipore), SEC22b (A304‐601A, Bethyl Laboratories), anti‐Syntaxin‐5 (SC‐365124, Santa Cruz), anti‐GM130(Cell Signaling; mouse), α‐GM130 (Abcam), anti‐α‐Dystroglycan (IIH6C4, Millipore 05‐593) and α‐PDI (Sigma Aldrich), and α‐mouse and α‐rabbit (Cy2 and Cy3 from Dianonva). Mammalian expression plasmid was generated by gene synthesis (GeneArt^®^, Regensburg, Germany) of the cDNA of the transcript variant 1 of BET1 (NM_005868) and cloning into the expression vector pFrog‐HA, resulting in an N‐terminal HA‐tagged version of BET1. For the yeast expression plasmid, cDNA of BET1 was amplified via PCR and cloned into pRS316. Variants were introduced by site‐directed mutagenesis using mutagenesis kits (New England Biolabs, Frankfurt am Main, Germany and Agilent, Waldbronn, Germany). All constructs were verified by sequencing.

### SDS–PAGE and immunoblotting

#### Skeletal muscle biopsy

Biopsies from normal control and P1 were homogenized using lysis buffer containing 4% SDS, 125 mM Tris–HCl (pH 8.8), 40% Glycerol, 500 µM PMSF, and 100 mM DTT. The lysates were sonicated on ice followed by centrifugation. Total protein from the supernatants was electrophoresed on NuPAGE gradient gels (Invitrogen, USA) and transferred to PVDF membrane. After blocking with 5% non‐fat milk in TBST for 30 min, the immunoblot membrane for anti‐α‐Dystroglycan (IIH6C4, Millipore 05‐593) was incubated overnight at 4°C, subsequently incubated with IRDye^@^ 680RD Goat anti‐mouse‐IgM secondary antibody (1:5,000, 926‐68180, LI‐COR) and imaged on the Odyssey CLx Imaging System (LI‐COR).

#### Fibroblasts

Fibroblasts were grown in DMEM and 10% FBS supplemented with Penicillin/Streptomycin until they reached confluency. Proteins were extracted from normal control and patients’ fibroblasts using RIPA buffer with PhosStop (Sigma‐Aldrich, USA) and cOmplete mini protease inhibitor cocktail (Sigma‐Aldrich, USA). Insoluble material was removed by centrifugation at 10,000 *g* for 10 min. The 550 µg of solubilized supernatant was added to 50 μl of wheat germ agglutinin (WGA) (Vector Labs, USA) bring up total volume to 500 μl using RIPA buffer with PhosStop and protease inhibitor. The samples were rotated overnight at 4°C. The pelleted WGA beads were washed three times with 500 μl of RIPA buffer. After the final wash, 25 μl of 4× NuPAGE LDS buffer (Thermo Fisher, USA) was added to the beads. The samples were heated to 95°C for 5 min before loading into 3–8% Tris‐Acetate gel (Invitrogen, USA), and protein was subsequently transferred to PVDF membrane. After blocking in PBS blocking buffer (LI‐COR Bioscience, USA), the immunoblot membrane for anti‐α‐Dystroglycan (IIH6C4, Millipore, USA) was incubated overnight at 4°C and subsequently incubated with IRDye^@^ 680RD Goat anti‐mouse‐IgM secondary antibody (LI‐COR Bioscience, USA). All antibodies were diluted in the same buffer that was used for blocking. The membrane was washed in PBST and scanned using an Odyssey CLx Imaging System (LI‐COR Bioscience, USA). HSP70 (BD, USA) was used as loading control. Densitometry was done on Odyssey CLx Imaging System (LI‐COR Bioscience, USA).

Alternatively, fibroblasts were washed in PBS, cells were harvested and lysed in EBC buffer (50 mM Tris–HCl, 120 mM NaCl, 0.5% NP‐40, and 5 mM EDTA) containing complete protease inhibitor cocktail (Roche) by using sonication. Remaining cellular debris was removed by centrifugation, and protein concentrations were determined by BCA (Thermo Fisher or DS‐assay (Bio‐Rad, Germany)). Equal amounts of proteins were resuspended in SDS–PAGE sample buffer and subjected to SDS–PAGE. Proteins were transferred to nitrocellulose membrane and processed as described above.

### BET1 immunocytochemistry

Skin fibroblasts were seeded (1 × 10^3^ cells per well) in 8‐chamber tissue culture slides for 20 h. Cells were fixed in pre‐cold 100% methanol for 5 min at room temperature and incubated with primary antibody mouse monoclonal IgG2a against BET1 (1:300, SC‐136390, Santa Cruz) and GOSR2 (membrin,1:100, 170003, Synaptic Systems), Rabbit polyclonal IgG SEC22b (1:50, A304‐601A, Bethyl Laboratories), mouse monoclonal IgG1 anti‐Syntaxin‐5 (1:50, SC‐365124, Santa Cruz) in 5% BSA in PBS for 1.5 h at room temperature. After washing, cells were incubated with Alexa Fluor 568 goat anti‐mouse IgG2a (1:500, A21134, Thermo Fisher Scientific) and Alexa Fluor 488 goat anti‐mouse IgG1 (1:500, A21121, Thermo Fisher Scientific)‐conjugated secondary antibodies at room temperature for 1 h. The nuclei were counterstained by 4’,6‐diamidino‐2‐phenylindole. Images were acquired on a TCS SP5 II system (Leica Microsystems, Buffalo Grove, IL) with the 40× objective.

Alternatively, fibroblasts were seeded on coverslips the day before fixation, which was done with MeOH (chilled to −20°C) for 10 min at RT followed by three washing steps with PBS. Cells were blocked with 1% goat serum in PBS for 1 h at RT and subsequently incubated with primary antibodies diluted in 1% goat serum in PBS (α‐BET1 (Santa Cruz; 1:300), α‐ERGIC‐53 (Abcam; 1:100), α‐GM130 (Abcam; 1:400), α‐GM130 (Cell Signaling; 1:400), and α‐PDI (Sigma Aldrich; 1:400) and incubated at 4°C overnight. Incubation with secondary antibody (α‐mouse and α‐rabbit Cy2 and Cy3 from Dianonva) was performed in 1% goat serum in PBS for 1 h at RT and nuclear staining with Hoechst solution for 10 min at RT. Coverslips were mounted with Mowiol. Images were acquired on a LSM700 (Carl Zeiss, Oberkochen, Germany) with the 40X objective.

### siRNA knockdown

HeLa‐M (C1) cells were seeded on coverslips in a 24‐well plate the day prior siRNA transfection. Cells were transfected with 5 pmol siRNA against *BET1* #1 (5`‐ATGGGAACTATGGCTATGCTA), #2 (5`‐CACAACTGGATTTCTAGGTAA), or negative control (Qiagen, Düsseldorf, Germany) with Lipofectamin RNAiMax from Thermo Fisher (Schwerte, Germany) according to manufacture protocol. Two days post‐transfection cells were analyzed by confocal microscopy or by immunoblot.

### ER‐to‐Golgi transport assay

Two days post‐transfection, secretion of pC4 reporter construct was induced with incubation of 3.3 µM rapamycin (cayman chemicals, Michigan, USA) for 0 or 10 min. Cells were washed twice with ice‐cold PBS and fixed in 4% PFA in PBS. After permeabilization with 0.1% Triton X‐100 in PBS for 3 min, cells were blocked with 1% goat serum for 1h at RT. Primary antibody (anti‐GM130, mouse, cell signaling, 1:400 in 1% goat serum) was incubated over night at 4°C. Secondary antibody incubation with anti‐mouse Cy3 (Dianova) was performed in a 1:400 dilution in 1% goat serum for 1h at RT. Nuclear staining was performed with Hoechst 33342 (Hoechst, Frankfurt/Main, Germany) followed by mounting with Mowiol. Confocal microscopy images were taken with a LSM700 (Carl Zeiss, Oberkochen, Germany).

### Modeling and molecular dynamic simulations of the SNARE complexes

The wild‐type Golgi SNARE complex was modeled using Modeller v.9 (Sali & Blundell, 1993) based on the set of crystal structures of different SNARE complexes (PDB ID codes 2NPS, 2GL2, 1SFC, 3B5N, 4WY4). The complex was prepared for simulations using the Leap module of AmberTools (Schafmeister C. E. A. F., 1995). Simulations were run with the NAMD engine (Phillips *et al*, 2005) using the AMBER99SBildn force field (Hornak *et al*, 2006) and TIP3P parameters for water (Jorgensen *et al*, 1983). Each simulation box was minimized, equilibrated by Cα‐restrained heating in ten steps of 30K up to 300K for a total of 1 ns and further equilibrated by unrestrained heating. Subsequently, the production simulations were carried out at 300K and 1 atm, controlled with a Nosé–Hoover Langevin piston. After equilibration, three replica models were simulated for 50 ns. Trajectories were analyzed with VMD software modules and Tcl scripts. For hydrogen bond contact measurements, a cutoff distance of 3.6 Å between heavy atoms and an angle cutoff of 30° were used. Mutant variants were generated by replacing corresponding residues in one of simulated wt models. Models were minimized with Rosetta algorithm with subsequent analysis using Chimera (Pettersen *et al*, 2004).

### Mass spectrometry‐based proteomic analysis

The precipitated protein pellets were solubilized in 100 µl of 8 M urea for 30 min, 100 µl of 0.2% ProteaseMAX (Promega) was added, and the mixture was incubated for an additional 2 h. The protein extracts were reduced and alkylated as described previously (Chen *et al*, 2008), followed by the addition of 300 µl of 50 mM ammonium bicarbonate, 5 µl of 1%ProteaseMAX, and 20 µg of sequence‐grade trypsin (Promega). Samples were digested overnight in a 37°C thermomixer (Eppendorf).

For Orbitrap Fusion Tribrid MS analysis, the tryptic peptides were purified with Pierce C18 spin columns and fractionated with increasing ACN concentration (15%, 20%, 30%, 40%, 60%, and 70%). Three micrograms of each fraction were auto‐sampler loaded with a Thermo Fisher EASY nLC 1000 UPLC pump onto a vented Acclaim Pepmap 100, 75 µm × 2 cm, nanoViper trap column coupled to a nanoViper analytical column (Thermo Fisher 164570, 3 µm, 100 Å, C18, 0.075 mm, 500 mm) with stainless steel emitter tip assembled on the Nanospray Flex Ion Source with spray voltage of 2000 V. Buffer A contained 94.785% H_2_O with 5% ACN and 0.125% FA, and buffer B contained 99.875% ACN with 0.125% FA. The chromatographic run was for 4 h in total with the following profile: 0–7% for 7 min, 10% for 6 min, 25% for 160 min, 33% for 40 min, 50% for 7 min, 95% for 5 min, and 95% again for 15 min. Additional MS parameters include the following: ion transfer tube temp = 300°C, Easy‐IC internal mass calibration, default charge state = 2, and cycle time = 3 s. Detector type set to Orbitrap, with 60 K resolution, with wide quad isolation, mass range = normal, scan range = 300–1500 m/*z*, max injection time = 50 ms, AGC target = 200,000, microscans = 1, S‐lens RF level = 60, without source fragmentation, and datatype = positive and centroid. MIPS was set as on, included charge states = 2–6 (reject unassigned). Dynamic exclusion enabled with *n* = 1 for 30 and 45 s exclusion duration at 10 ppm for high and low. Precursor selection decision = most intense, top 20, isolation window = 1.6, scan range = auto normal, first mass = 110, collision energy 30%, CID, Detector type = ion trap, Orbitrap resolution = 30 K, IT scan rate = rapid, max injection time = 75 ms, AGC target = 10,000, Q = 0.25, inject ions for all available parallelizable time.

### Tandem mass spectra analysis

Peptide spectral files from pooled samples or from biological replicates were combined for database searching. Spectrum raw files were extracted into MS1 and MS2 files using in‐house program RawXtractor or RawConcerter (http://fields.scripps.edu/downloads.php; He *et al*, 2015), and the tandem mass spectra were searched against UniProt mouse protein database (downloaded on UniProt_Human_proteome_cont_03‐25‐2014) and matched to sequences using the ProLuCID/SEQUEST algorithm (ProLuCID version 3.1; Eng *et al*, 1994; Xu *et al*, 2006).

The search space included all fully and half‐tryptic peptide candidates that fell within the mass tolerance window with no miscleavage constraint, assembled and filtered with DTASelect2 (version 2.1.3; Tabb *et al*, 2002; Cociorva *et al*, 2007) through Integrated Proteomics Pipeline (IP2 version 3, Integrated Proteomics Applications, http://www.integratedproteomics.com). To estimate peptide probabilities and false‐discovery rates (FDR) accurately, we used a target/decoy database containing the reversed sequences of all the proteins appended to the target database (Peng *et al*, 2003). Each protein identified was required to have a minimum of one peptide of minimal length of six amino acid residues; however, this peptide had to be an excellent match with a FDR < 0.001 and at least one excellent peptide match. After the peptide/spectrum matches were filtered, we estimated that the protein FDRs were ≤ 1% for each sample analysis.

### Volcano plot

Graphic illustration of the differences in AP‐MS data of HA‐BET1 WT vs. HA‐BET1 Ile51Ser expressing HEK cells was done with the software Perseus from the Max Planck Institute of Biochemistry (Martinsried, Germany) according to their instructions (Tyanova *et al*, 2016). First, the spectral counts of three independent AP‐MS experiments were categorical annotated by rows. Next, data sets were transformed to log_2_(x) and subsequent filtered for 3 valid values in at least one group. Missing values were imputated from normal distribution assuming a width of 0.3 and a down shift of 1.8. For volcano plot −log *P*‐values were plotted against the difference of HA‐BET1 WT and HA‐BET1 Ile51Ser. Significant cutoff was chosen for changes greater than 2‐fold and *P*‐values equal/lower than 0.01.

### Co‐immunoprecipitations

For HA‐immunoprecipitation, cells were transfected with cDNA of HA‐BET1 wild‐type and variants or GFP as control. After one day of expression, cells were harvested and subsequent lysed in EBC with complete by sonification. Cell debris was removed by centrifugation and supernatant subjected to immunoprecipitation. A fraction of the supernatant was kept as input sample. HA‐sepharose beads (Pierce™, USA) blocked with 5% BSA in EBC buffer were incubated with residual supernatant for 2 h at 4°C. Beads were collected by centrifugation at 800 *g* and 4°C and washed three times with 500 µl EBC buffer. Precipitates were eluted with Laemmli buffer (Bio‐Rad) containing β‐mercaptoethanol at 55°C for 30 min. For endogenous immunoprecipitation of BET1, HEK293 cells were harvested and subsequent lysed in EBC + complete by sonification. After centrifugation for removing cell debris, lysate was incubated with anti‐Bet1 antibody (Santa Cruz, USA) over night at 4°C. As controls served lysis buffer without cells and cell lysate without antibody. 50 µl lysate was treated with Laemmli buffer as input sample. Magnetic protein A/G beads (Pierce™, USA) were blocked with 5% BSA in EBC over night at 4°C and subsequent incubated with the IP‐sample at 4°C. After 3× washing with EBC buffer, co‐IP samples were eluted with reducing Laemmli buffer.

### Brefeldin A Golgi reconstitution assay

Fibroblasts were seeded on coverslips the day prior the assay. The next day, cells were incubated with media containing 1 µg/ml Brefeldin A (Sigma‐Aldrich, Steinheim, Germany) for 40 min at 37°C to disrupt Golgi structures. Cells were washed 3× with PBS and cultured for indicated time points in fresh media lacking Brefeldin A. Cells were washed with PBS and fixed with 4% PFA. Immunofluorescence was performed with anti GM130 antibody (Cell Signaling; mouse 1:400). Golgi reconstitution was analyzed by confocal fluorescence microscopy. Three independent experiments were performed with at least six images per time point and cell line. For quantification, the number of cells having an intact Golgi was dived by the number of total cells per image. Therefore, images were blinded and randomized.

### Survival assay of temperature‐sensitive yeast strain

The temperature‐sensitive yeast strain *bet1 ts* BSH‐7C (Boehm *et al*, 1994) cannot thrive at 30°C or higher temperatures, unless a functional copy of *bet1* is transformed and expressed. The *bet1* ts strains containing pRS316 plasmids expressing bet1, bet1 p. Leu77Ile or bet1Leu77Ser, were grown for 12 h in standard minimal medium with appropriate supplements (SD‐URA). Equal ODs with serial dilutions (1:10) were plated on SD‐URA and incubated at 24°C, 30°C, or 33°C for 48 h. Only yeast strains expressing a functional copy of bet1 can rescue the ts phenotype of the *bet1* ts strain at 30°C and 33°C.

### Statistical analyses

Statistical analyses were performed with GraphPad Prism7. Protein levels in immunoblot analysis were normalized to GAPDH of three independent replicates.

## Author contributions

SD, NK, MD, DF MS, and CGB conceptualized and designed the study. SD, NK, MD, JK, PY, BBC, YH, SG, JGH, VSG, PEF, SS, RS, PU, PSG, KGM, SE, STI, KRC, MS, SLE, DB‐G, STI, MDP, ARF, SV, JGH, SS, VSG, JNS, and VB acquired and analyzed the data. SD, MS, and CGB drafted the manuscript. MS and CGB supervised the study. All authors critically revised the manuscript.

## Conflict of interest

KGM is an employee of GeneDx, Inc. The authors declare that they have no conflict of interest.

## For more information

1. dbGaP, https://www.ncbi.nlm.nih.gov/gap
2. gnomAD Browser, https://gnomad.broadinstitute.org
3. Geno_2_MP, NHGRI/NHLBI University of Washington‐Center for Mendelian Genomics (UW‐CMG), Seattle, WA, http://geno2mp.gs.washington.edu [September 2021]
4. ClinVar, http://www.ncbi.nlm.nih.gov/clinvar/
5. Ensembl, http://www.ensembl.org/index.html
6. Genome Aggregation Database (gnomAD), http://gnomad.broadinstitute.org/
7. GTEx Portal, https://www.gtexportal.org/home/
8. Human Splicing Finder, http://www.umd.be/HSF3/index.html
9. MutationTaster, http://www.mutationtaster.org
10. OMIM, http://www.omim.org
11. NHLBI Exome Sequencing Project (ESP) Exome Variant Server, http://evs.gs.washington.edu/EVS/
12. RefSeq, http://www.ncbi.nlm.nih.gov/RefSeq
13. SpliceAI, https://spliceailookup.broadinstitute.org
14. UniProt, http://www.uniprot.org/
15. Integrated Proteomics Pipeline, http://www.integratedproteomics.com
16. RawConcerter, http://fields.scripps.edu/downloads.php

## Supporting information



Expanded View Figures PDF

Table EV1

Source Data for Figure 1

Source Data for Figure 3

Source Data for Figure 5

## Data Availability

All data generated in this study are available upon request from the authors. All requests will be reviewed by the respective institution to verify if the request is subject to any intellectual property or confidentiality obligations. Phenotype data and candidate gene/variant information are shared through *Seqr* with the Matchmaker Exchange system (https://www.matchmakerexchange.org/). RNA‐Seq data: dbGaPphs001272.v1.p1 (https://www.ncbi.nlm.nih.gov/projects/gap/cgi‐bin/study.cgi?study_id=phs001272.v1.p1). WES data (P1): AnVIL, AnVIL_CMG_Broad_Muscle_Bonnemann_WES. 9 (https://anvil.terra.bio/#workspaces/anvil‐datastorage/AnVIL_CMG_Broad_Muscle_Bonnemann_WES). *BET1*c.202G>C variant: ClinVar 619194 (https://www.ncbi.nlm.nih.gov/clinvar/variation/619194/). *BET1* c.134delC variant: ClinVar 619186 (https://www.ncbi.nlm.nih.gov/clinvar/variation/619186/). *BET1*c.152T>G variant: ClinVar 977650 (https://www.ncbi.nlm.nih.gov/clinvar/variation/977650/).
