# Supplementary material for: BET1 variants establish impaired vesicular transport as a cause for muscular dystrophy with epilepsy
Source: EMBO Mol Med. 2021 Nov 15;13(12):e13787. doi: 10.15252/emmm.202013787 (PMC8649873; doi:10.15252/emmm.202013787)
Supplement: Supplementary file 1 — Expanded View Figures PDF [file EMMM-13-e13787-s003.pdf]

Expanded View Figures

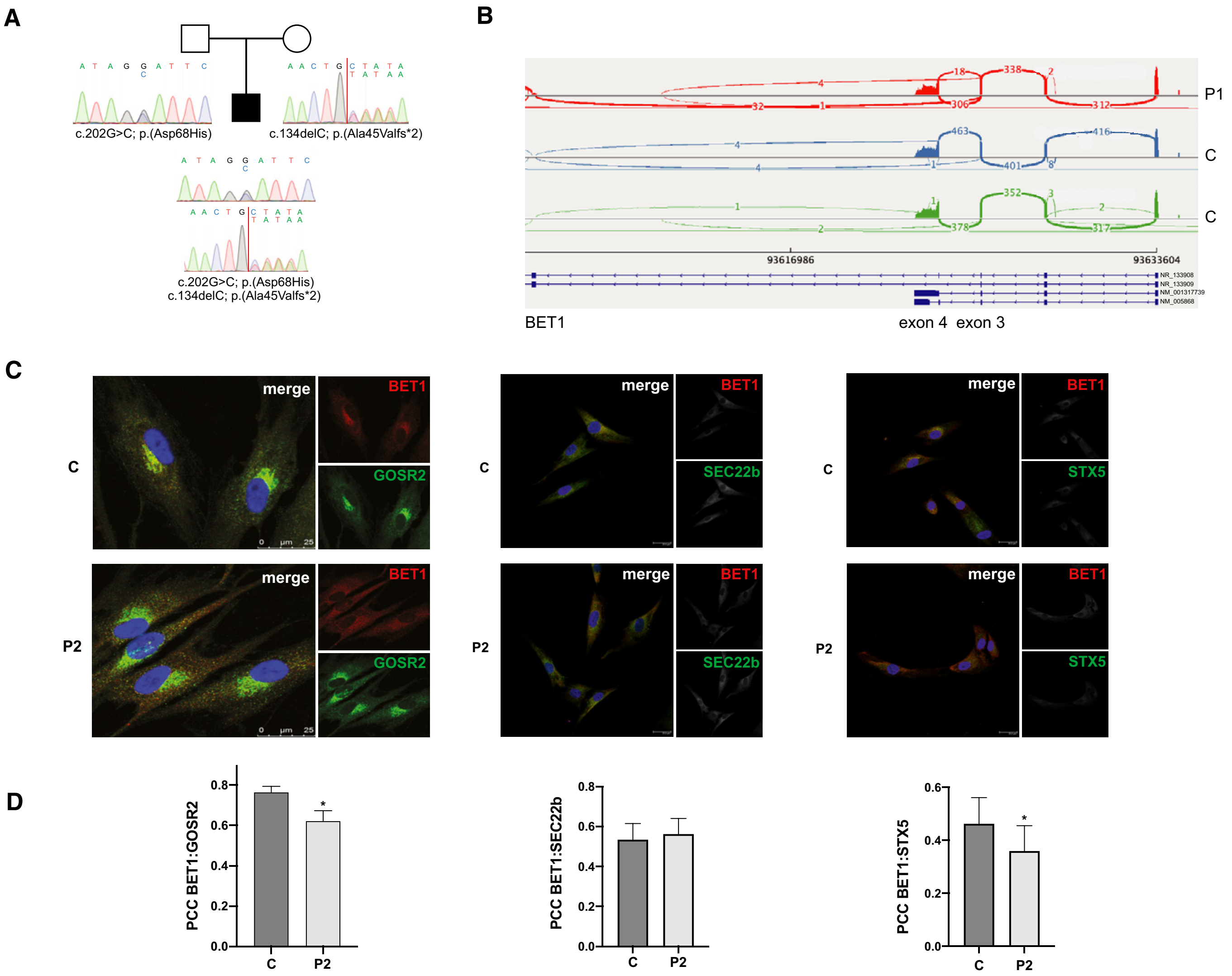

Figure EV1. Genetic studies in P1.

- A Pedigree of P1 with corresponding *BET1* Sanger chromatogram depicting the paternally inherited c.202G>C; p.(Asp68His) missense variant and a maternally inherited c.134delC; p.(Ala45Valfs*2) frameshift variant (NM_005868), consistent with recessive inheritance.
- B Sashimi plots comparing fibroblast RNA sequencing reads in P1 (red) and two control muscle samples (blue and green) at the exons 3–5 of BET1.
- C Immunofluorescence images of BET1 (red) and GOSR2 (green) in fibroblasts from P1, P2, and control. In P1, BET1 immunoreactivity is reduced. Scale bar: 25 μm.
- D In the overlay, BET1 colocalization with GOSR2 and Syntaxin-5 is reduced in P2 compared to controls while BET1 colocalization with SEC22b is unchanged. BET1 colocalization was determined using Pearson's correlation coefficient (PCC) on regions of interest (ROIs) drawn around cells (GOSR2 P2: n = 56; C: n = 86, P = 0.0025) (Syntaxin-5 P2: n = 27; C: n = 32 P = 0.0006) (SEC22b P2: n = 27; C: n = 30, P = 0.2093). (*$P \leq 0.05$ Mann–Whitney U-test). Data represented are mean ± SEM.

 © 2021 The Authors. Published under the terms of the CC BY 4.0 license

A

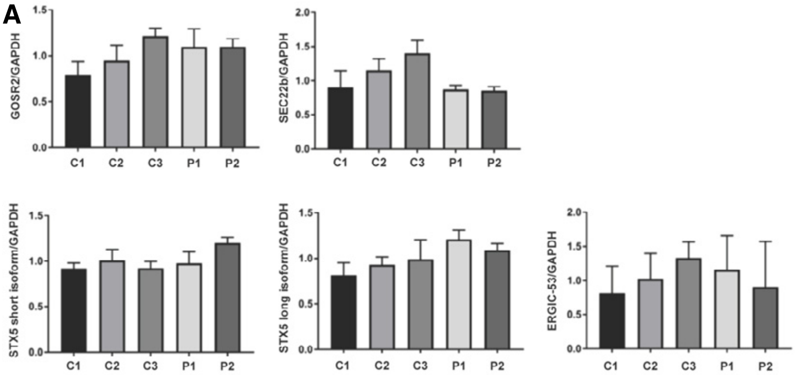

B

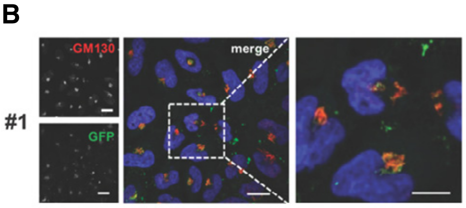

C D

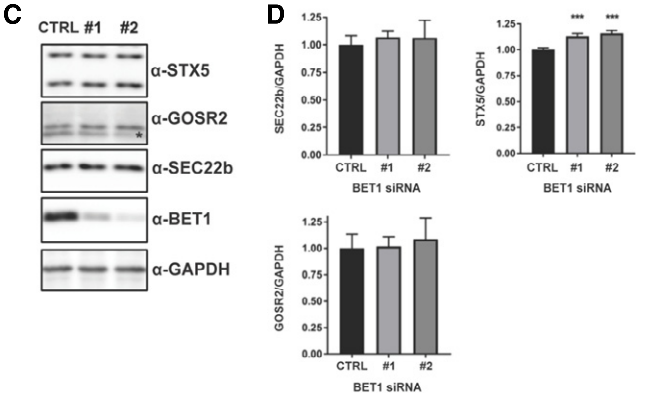

E

-Log p	Difference	Accession	gene name
3,999	5,404	Q9NRA8	EIF4ENIF1
4,157	5,292	P49257	ERGIC-53
3,884	4,912	Q9UMX0	UBQLN1
3,909	4,815	Q9NRR5	UBQLN4
1,847	4,766	E7EMW7	UBR5
3,113	4,700	Q99615	DNAJC7
1,904	4,657	O95071	UBR5
4,250	4,603	Q8TAT6	NPLOC4
4,626	4,348	Q15276	RABEP1
3,648	4,236	D6RG13	RPS3A
3,174	4,094	Q6Y7W6	GIGYF2
2,253	3,995	M0R165	EPS15L1
2,733	3,950	Q96CU9	FOXRED1
2,449	3,860	Q9UBC2	EPS15L1
3,475	3,666	E9PFK9	RABGEF1
3,234	3,662	Q8NBM4	UBAC2
2,571	3,630	P25686	DNAJB2
2,786	3,618	P25685	DNAJB1
3,401	3,615	Q9UJ41	RABGEF1
3,685	3,595	Q9NXW2	DNAJB12
2,709	3,557	Q13948	CUX1
3,404	3,364	Q15075	EEA1
3,539	3,209	Q9BRK5	SDF4
3,186	3,204	Q9Y4L5	RNF115
3,623	3,180	Q13049	TRIM32
3,732	-3,758	P12035	KRT3
2,932	-3,974	F8W6H6	MYO5A

Figure EV2. Normal SNARE complex partners in BET1 fibroblasts with reduced colocalization at the *cis*-Golgi in transfected cells.

A Quantification of protein levels of BET1 SNARE complex partners and the novel interaction partner ERGIC-53 in fibroblasts. Values are normalized to GAPDH. No significant differences between the control and patient fibroblasts were detected. Data represented are mean ± SD of four biological replicates. Significant differences were analyzed by one-way ANOVA followed by Tukey's *post hoc* test.

B Immunofluorescence images of *BET1* knockdown HeLa pC4 cells transfected with BET1 siRNA #1 10 min after induction of ER-to-Golgi transport with solubilizer. Scale bar: 20 μm.

C HeLa pC4 cells were transfected with control siRNA (ctrl) or siRNA against *BET1* (#1 and #2) and protein levels of BET1 and ER-to-Golgi complex SNAREs SEC22b, Syntaxin-5, and GOSR2 were analyzed by immunoblot. *residual SEC22b signal after immunoblot stripping.

D Quantification of EV2C. Data represented are mean ± SD of three biological replicates. Significant differences to ctrl cells were analyzed by one-way ANOVA followed by Dunnett's *post hoc* test. ***$P \leq 0.001$.

E Table showing a spectral count-based summary of three independent AP-MS experiments with significant hits shown in Fig 5A. Hits are sorted by the difference of BET1-WT and BET1-Ile51Ser. Hits which do not fulfill the criteria to have a mock-transfected spectral count of two or less are colored in grey.

© 2021 The Authors. Published under the terms of the CC BY 4.0 license

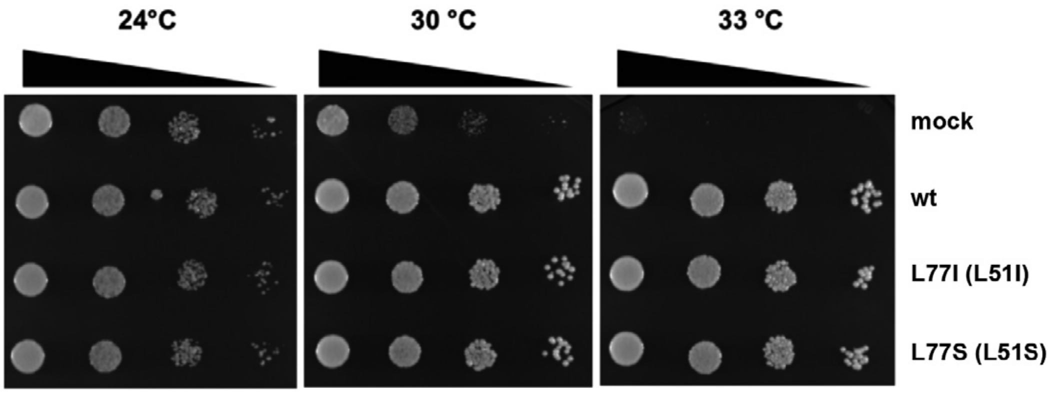

Figure EV3. The effect of BET1 variants on yeast growth.

BET1 Leu77Ile (Leu51Ile) and Leu77Ser (Leu51Ser) show similar growths compared to BET1 wild type. Temperature-sensitive strain of *S. cerevisiae* was transformed with wild-type BET1, the two different variants, and a mock plasmid. At 24°C, growth was detected for all variants and wild type, including the negative control. Only the negative control showed an impairment of growth at restrictive temperatures (30°C and 33°C).

 © 2021 The Authors. Published under the terms of the CC BY 4.0 license
