# Supplementary figures and images for: BET1 variants establish impaired vesicular transport as a cause for muscular dystrophy with epilepsy
Source: EMBO Mol Med. 2021 Nov 15;13(12):e13787. doi: 10.15252/emmm.202013787 (PMC8649873; doi:10.15252/emmm.202013787)

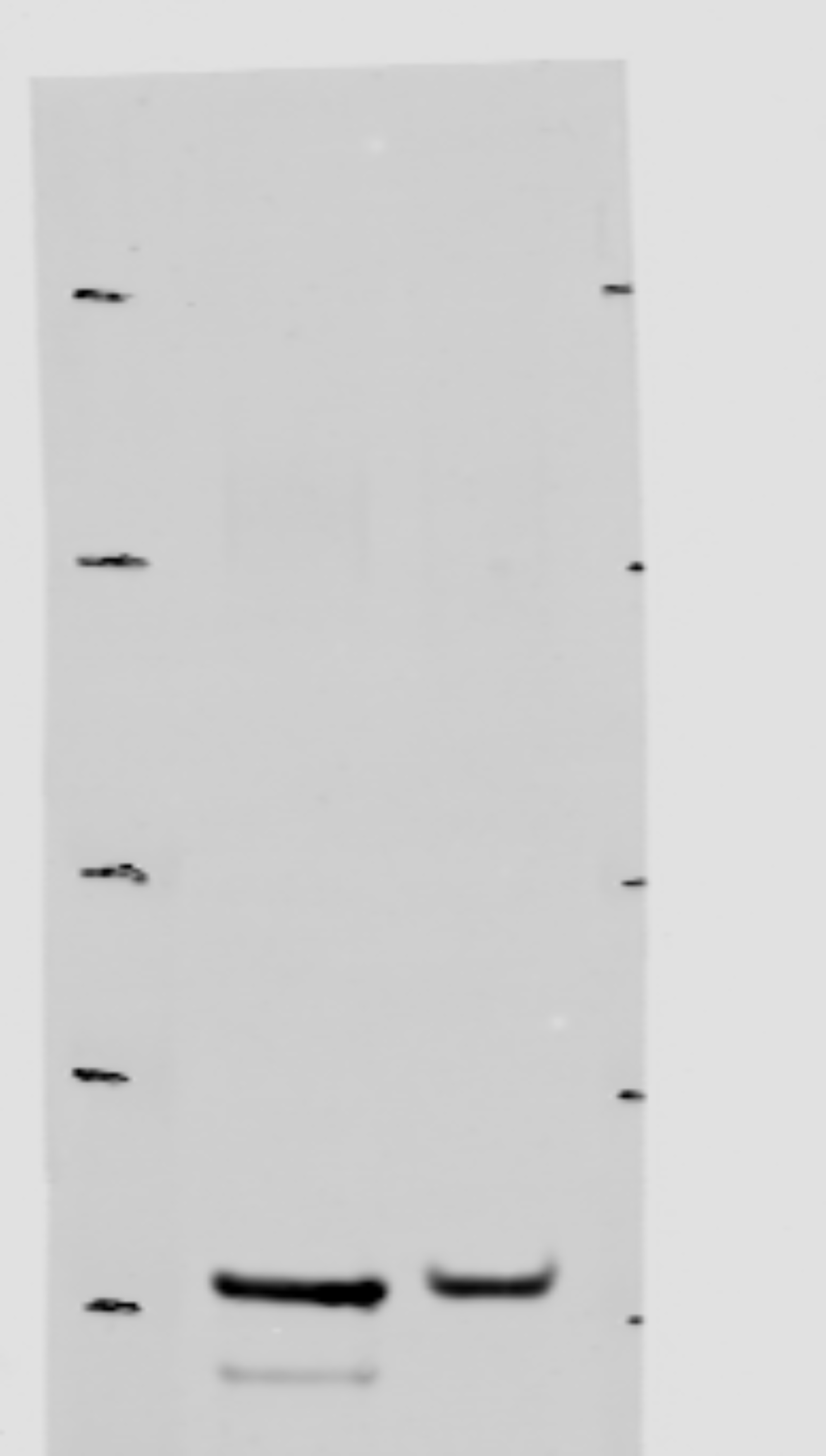

Supplement: Supplementary file 3 — Source Data for Figure 1 [file EMMM-13-e13787-s001.zip › EMM-2020-13787-V2_SourceDataForFigure1B/P1 GAPDH BW 8-22-18.tif]

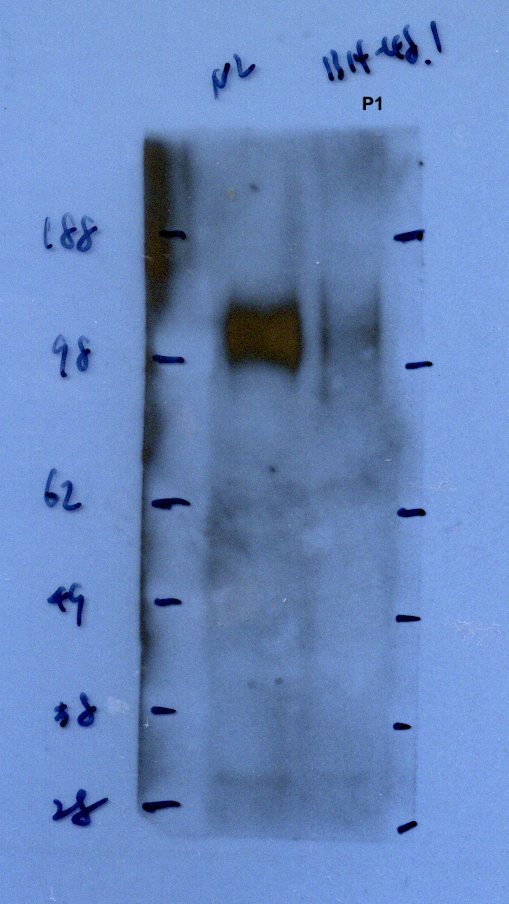

Supplement: Supplementary file 3 — Source Data for Figure 1 [file EMMM-13-e13787-s001.zip › EMM-2020-13787-V2_SourceDataForFigure1B/P1 a-DG 8-23.jpg]

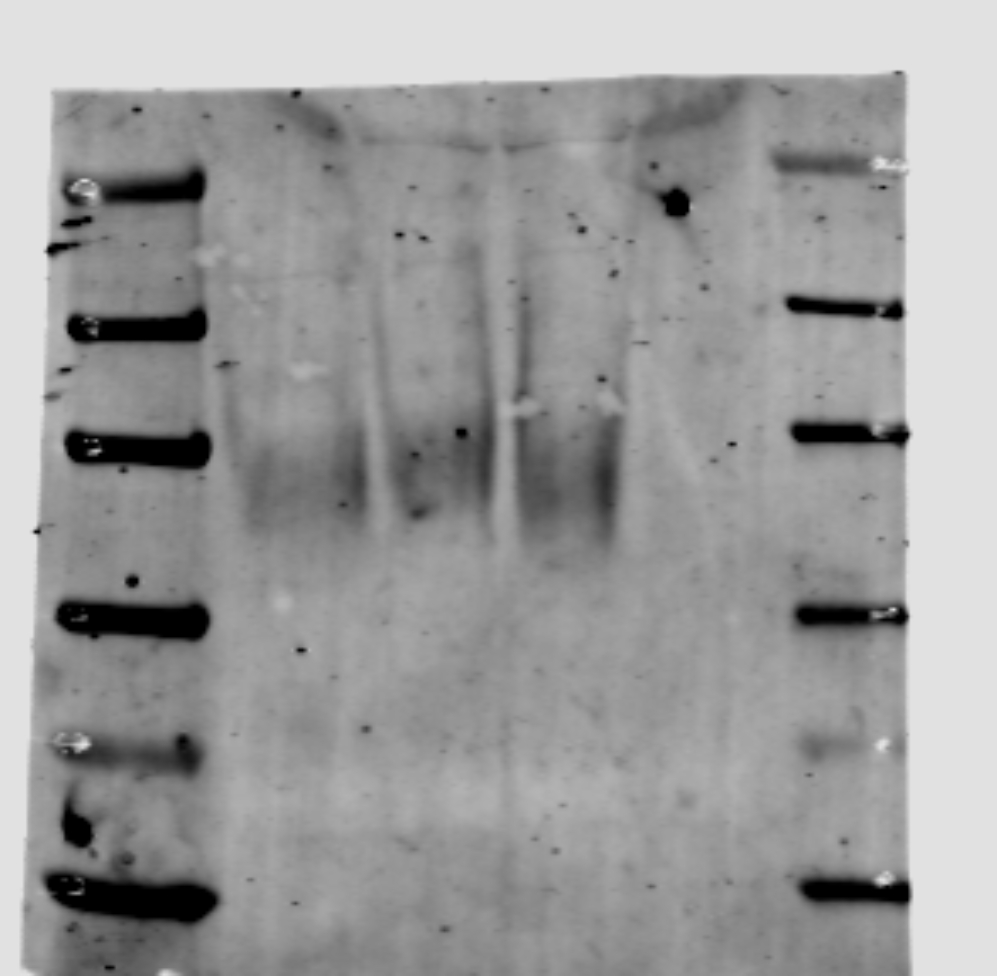

Supplement: Supplementary file 3 — Source Data for Figure 1 [file EMMM-13-e13787-s001.zip › EMM-2020-13787-V2_SourceDataForFigure1C/P1 P2 a-DG BW 3-15-2021.jpg]

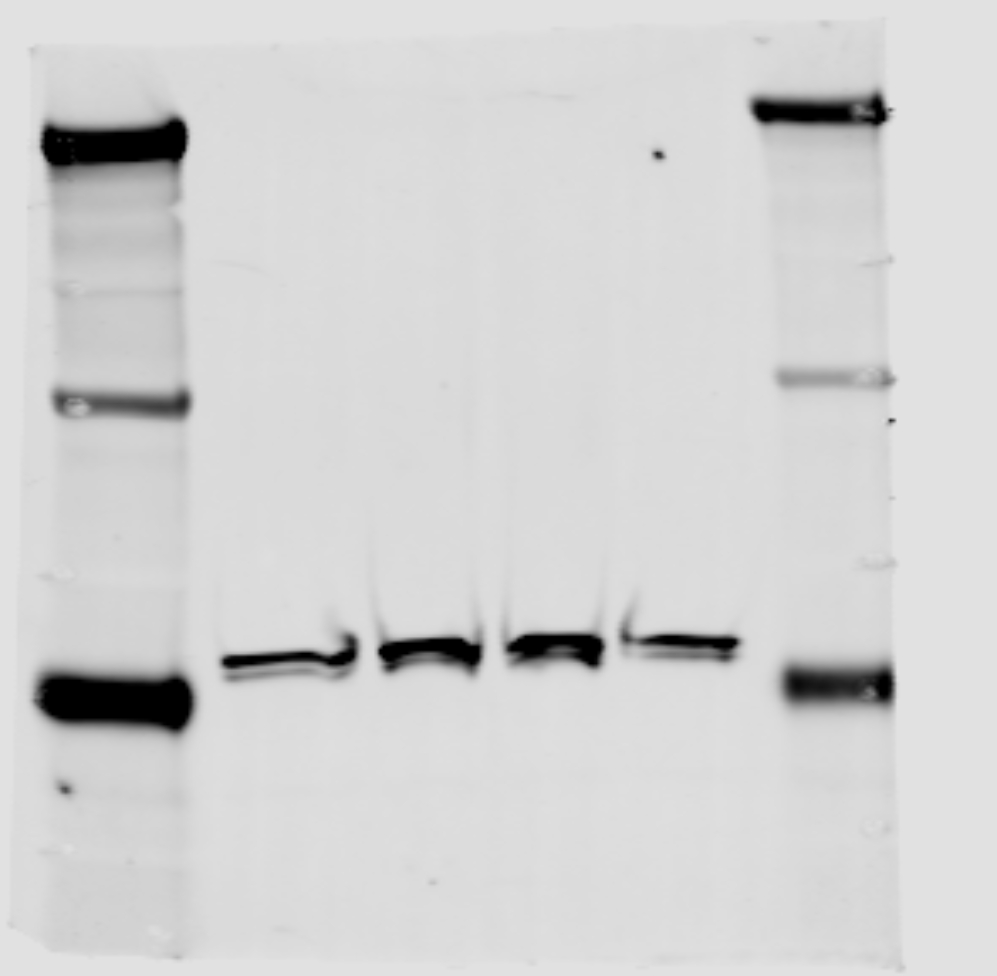

Supplement: Supplementary file 3 — Source Data for Figure 1 [file EMMM-13-e13787-s001.zip › EMM-2020-13787-V2_SourceDataForFigure1C/P1 P2 HSP70 BW 3-15-2021.jpg]

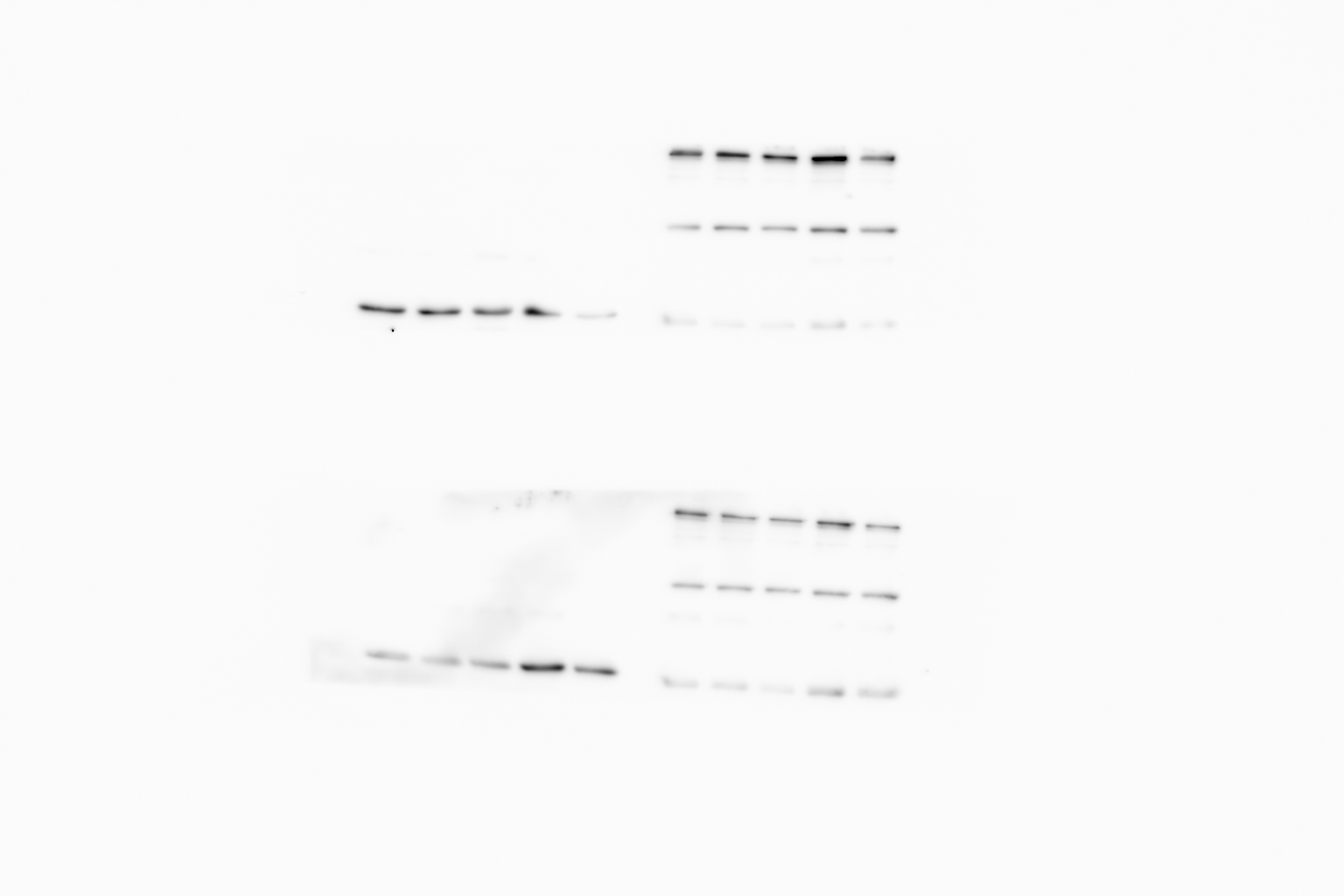

Supplement: Supplementary file 4 — Source Data for Figure 3 [file EMMM-13-e13787-s006.zip › EMM-2020-13787-V2_SourceDataForFigure3A/20200625_ERGIC53_1_2_100s.tif]

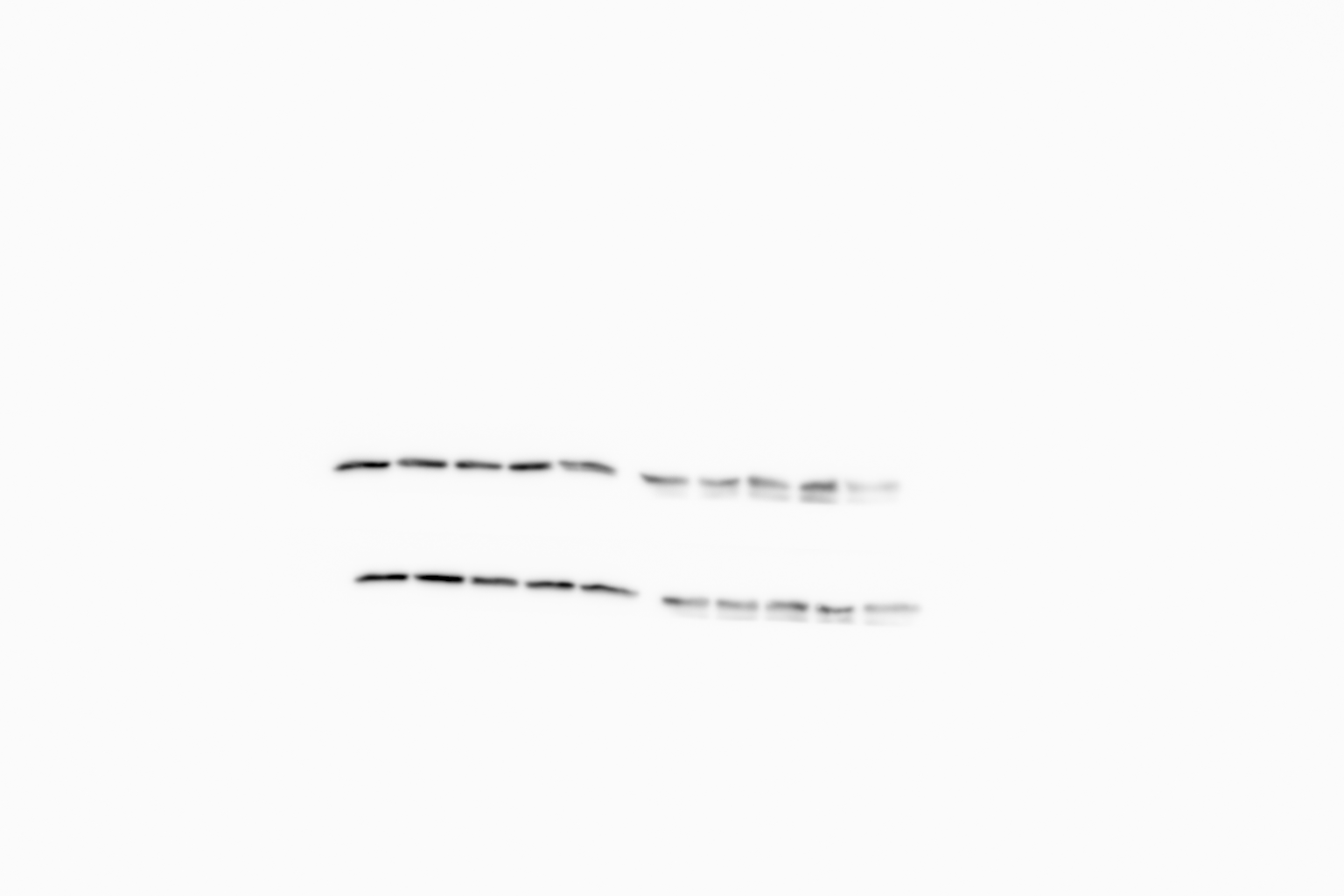

Supplement: Supplementary file 4 — Source Data for Figure 3 [file EMMM-13-e13787-s006.zip › EMM-2020-13787-V2_SourceDataForFigure3A/20200625_Sec22b_1_2_50s.tif]

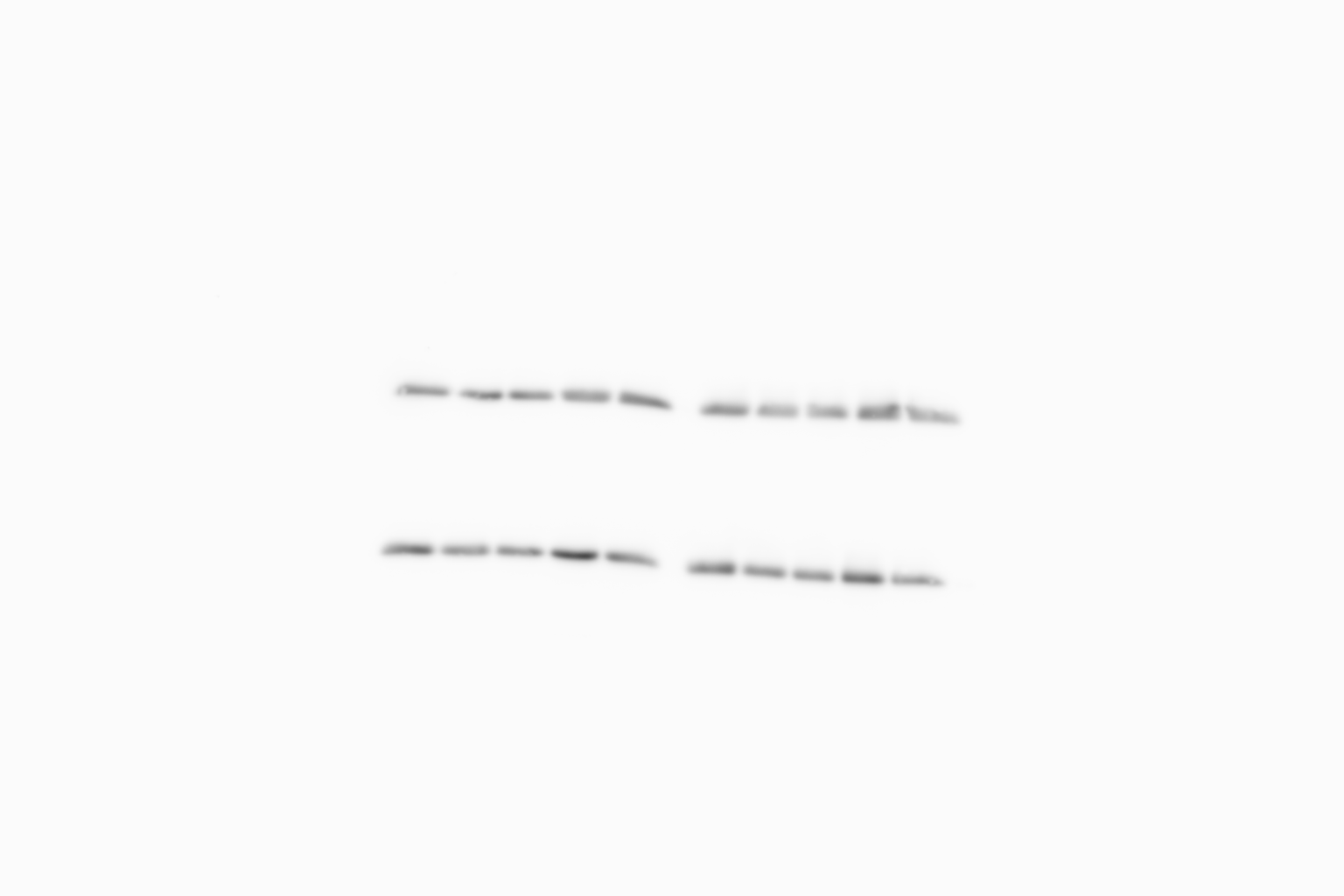

Supplement: Supplementary file 4 — Source Data for Figure 3 [file EMMM-13-e13787-s006.zip › EMM-2020-13787-V2_SourceDataForFigure3A/20200625_GOSR2_1_2_10s.tif]

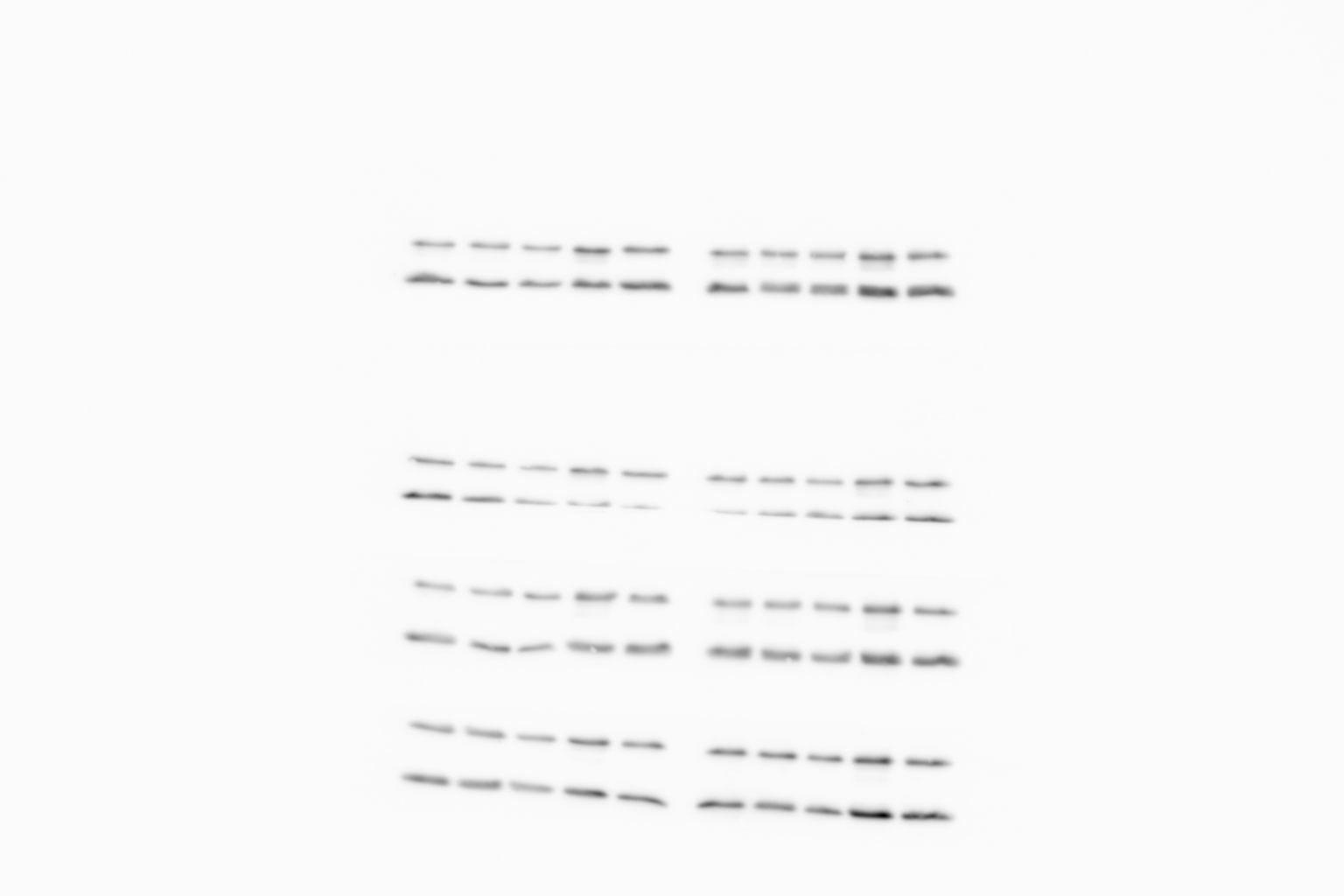

Supplement: Supplementary file 4 — Source Data for Figure 3 [file EMMM-13-e13787-s006.zip › EMM-2020-13787-V2_SourceDataForFigure3A/20200626_STX5_1_2_3_4_80s.tif]

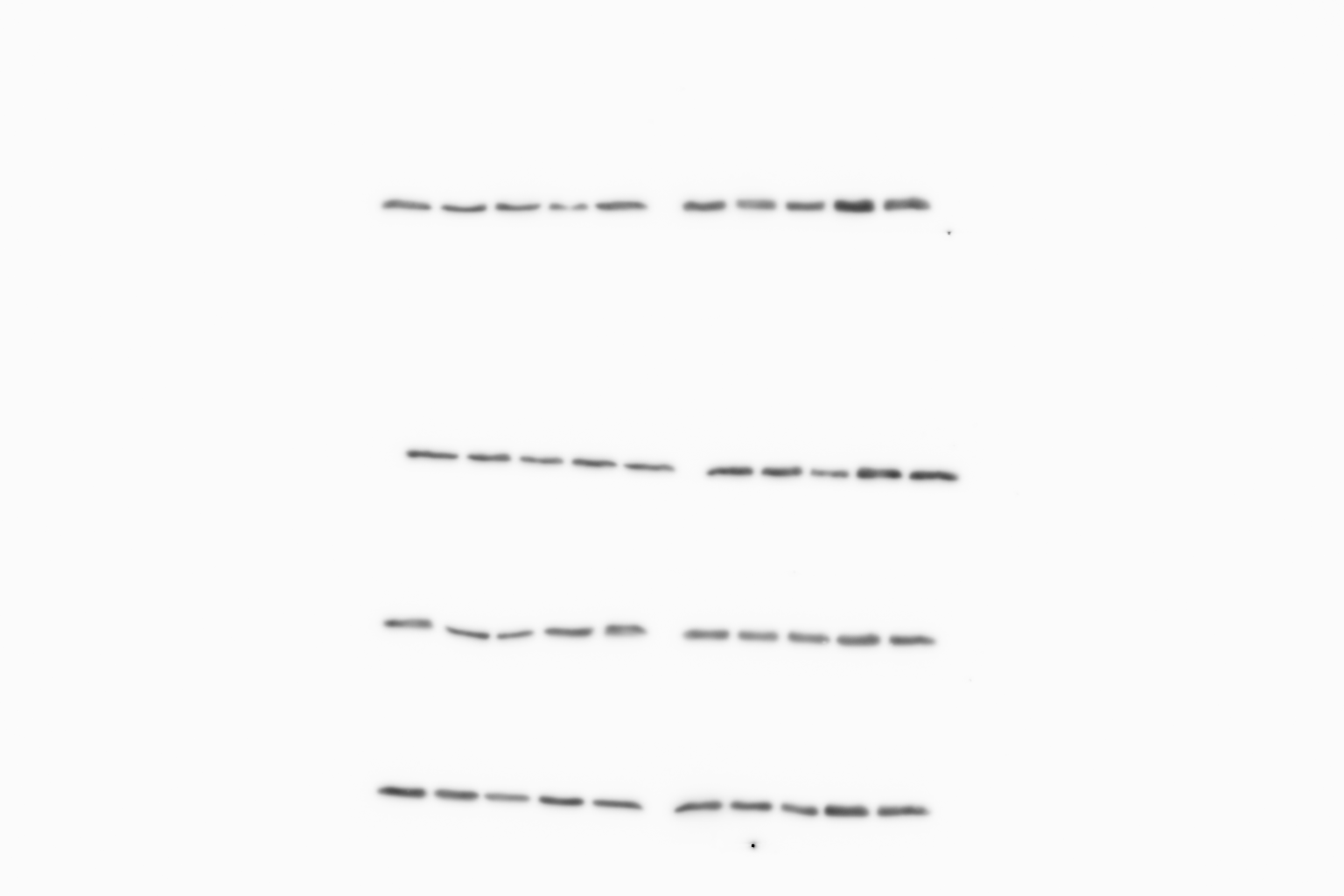

Supplement: Supplementary file 4 — Source Data for Figure 3 [file EMMM-13-e13787-s006.zip › EMM-2020-13787-V2_SourceDataForFigure3A/20200625_GAPDH_1_2_3_4_20s.tif]

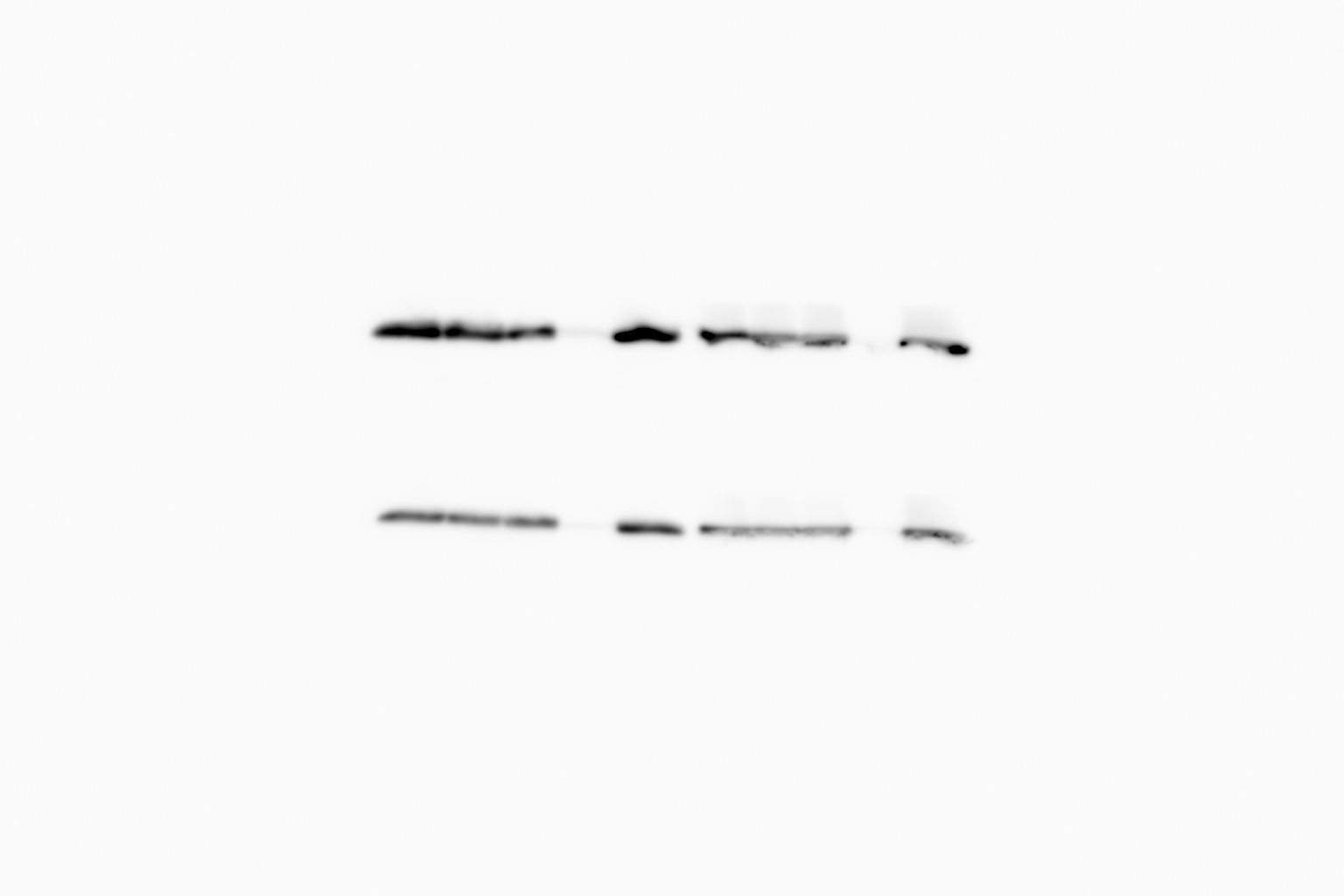

Supplement: Supplementary file 4 — Source Data for Figure 3 [file EMMM-13-e13787-s006.zip › EMM-2020-13787-V2_SourceDataForFigure3A/20200625_Bet1_1_2_110s.tif]

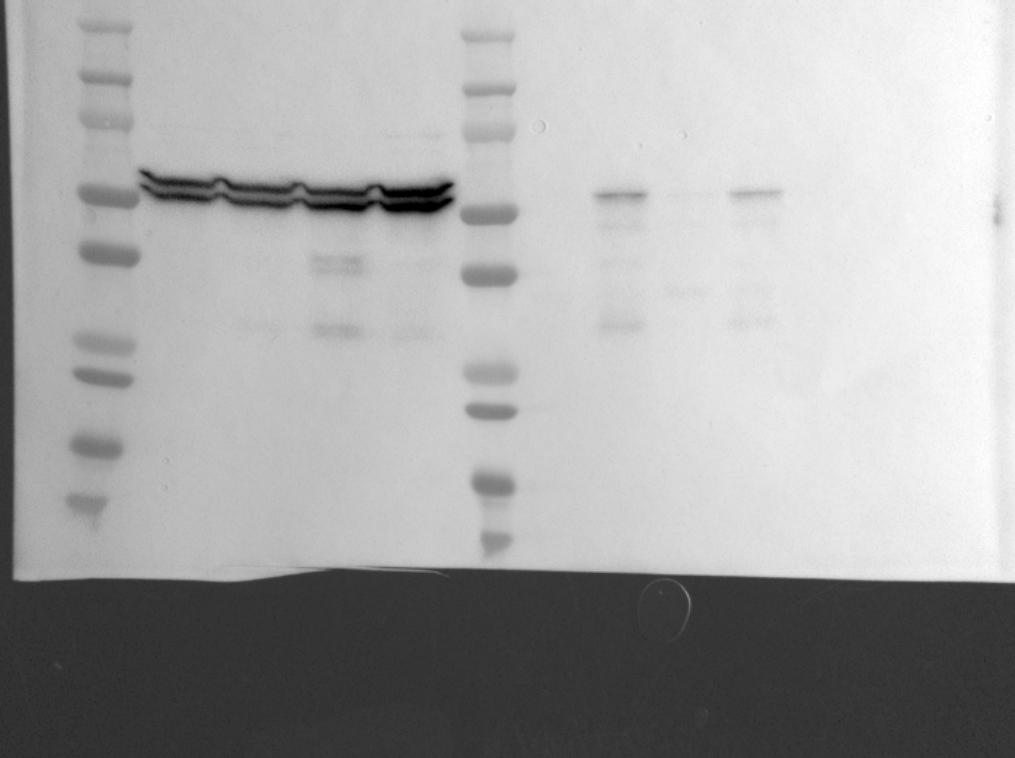

Supplement: Supplementary file 5 — Source Data for Figure 5 [file EMMM-13-e13787-s005.zip › EMM-2020-13787-V2_SourceDataForFigure5B/ERGIC53 2020-04-24 13hr 25min+2020-04-24 13hr 26min_Exposure_10.0sec.tif]

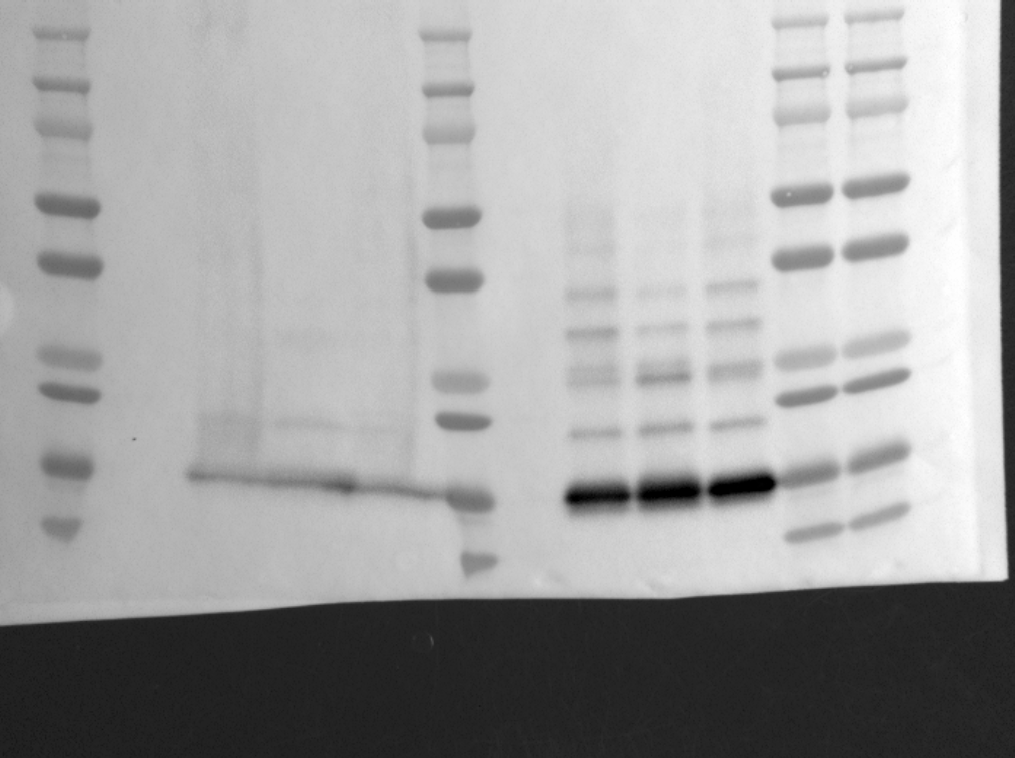

Supplement: Supplementary file 5 — Source Data for Figure 5 [file EMMM-13-e13787-s005.zip › EMM-2020-13787-V2_SourceDataForFigure5B/HA 2020-04-24 14hr 12min+2020-04-24 14hr 12min-1_Exposure_3.0sec.tif]

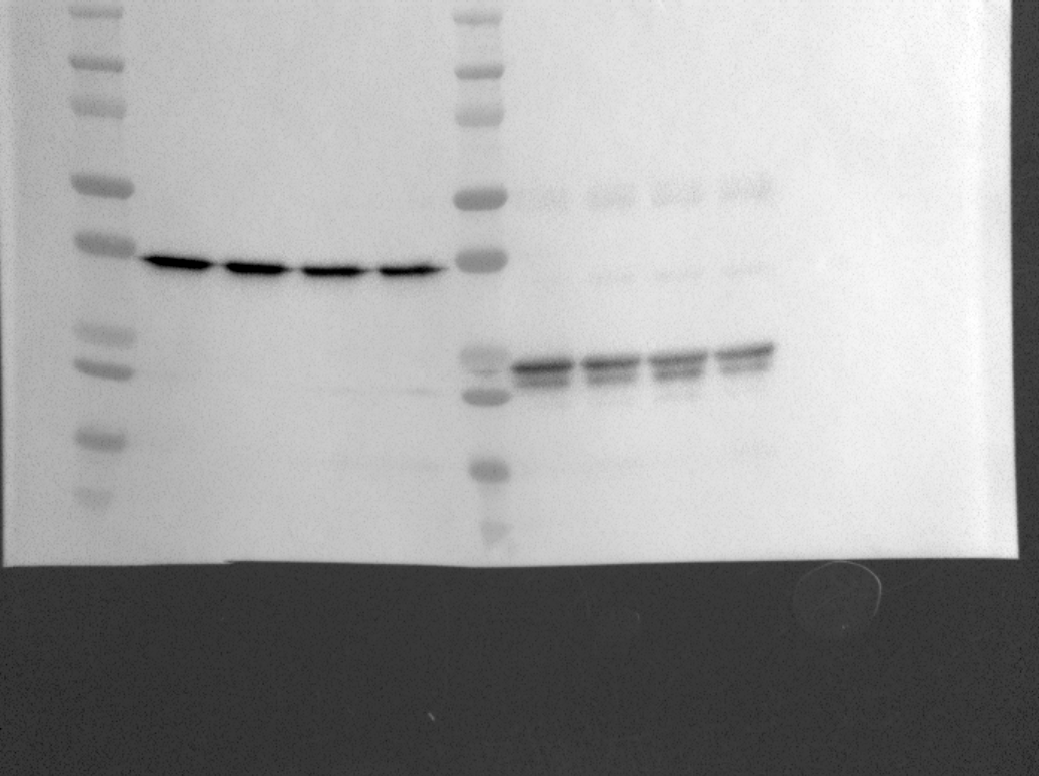

Supplement: Supplementary file 5 — Source Data for Figure 5 [file EMMM-13-e13787-s005.zip › EMM-2020-13787-V2_SourceDataForFigure5B/GAPDH 2020-04-29 15hr 51min_Exposure_20.0sec+2020-04-29 15hr 52min.tif]

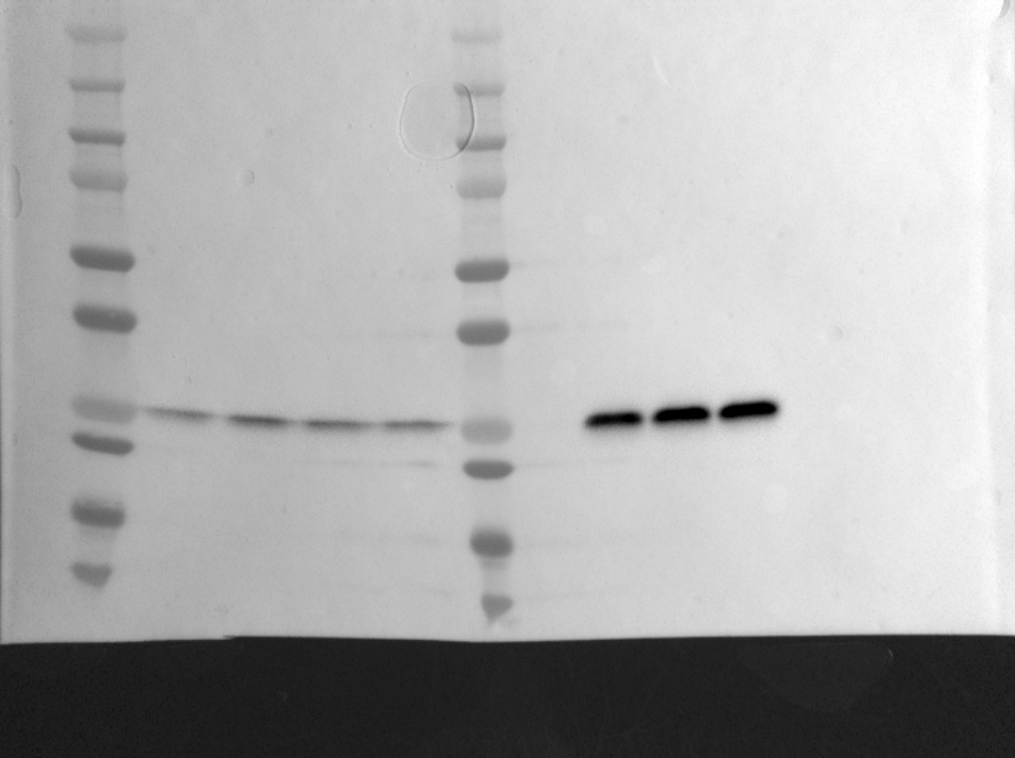

Supplement: Supplementary file 5 — Source Data for Figure 5 [file EMMM-13-e13787-s005.zip › EMM-2020-13787-V2_SourceDataForFigure5B/GOSR2 2020-04-24 14hr 01min+2020-04-24 14hr 02min_Exposure_6.0sec.tif]

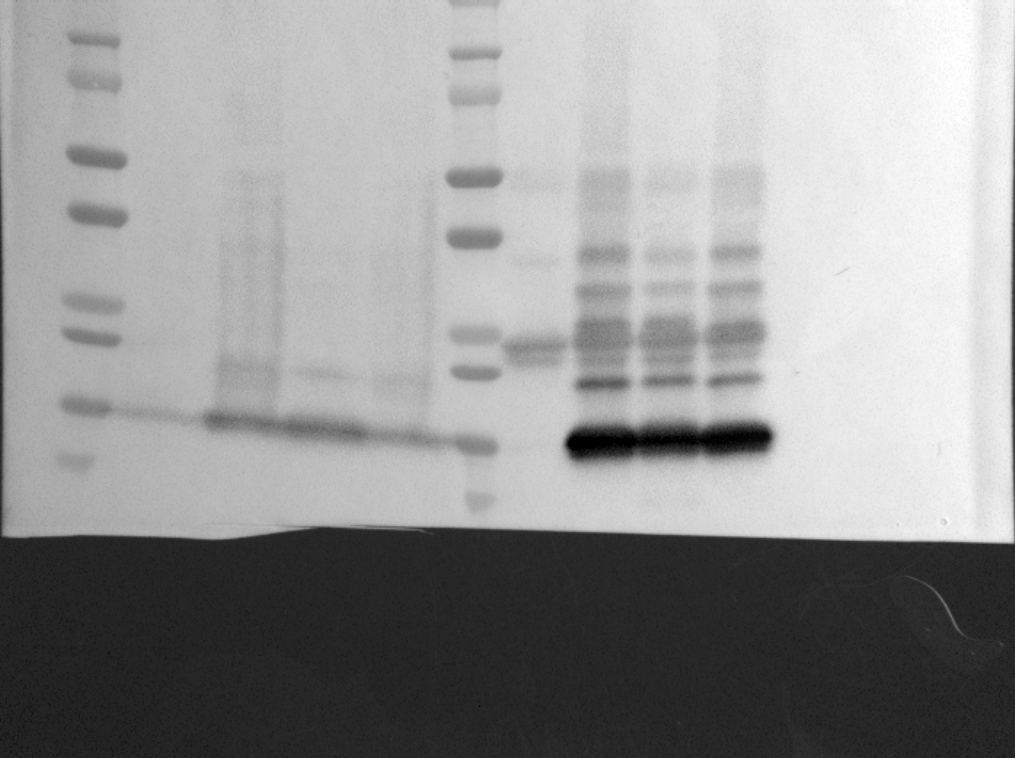

Supplement: Supplementary file 5 — Source Data for Figure 5 [file EMMM-13-e13787-s005.zip › EMM-2020-13787-V2_SourceDataForFigure5B/Bet1 2020-04-28 18hr 57min+2020-04-28 18hr 56min_Exposure_2.0sec.tif]

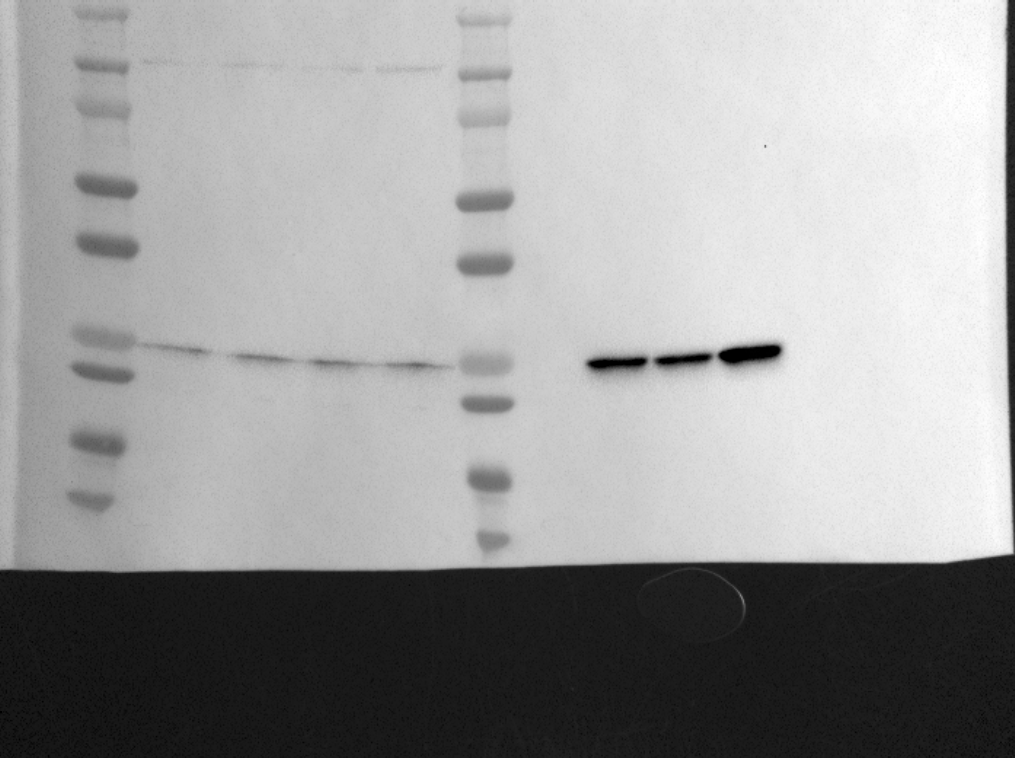

Supplement: Supplementary file 5 — Source Data for Figure 5 [file EMMM-13-e13787-s005.zip › EMM-2020-13787-V2_SourceDataForFigure5B/sec 22b 2020-04-24 13hr 48min+2020-04-24 13hr 46min_Exposure_20.0sec.tif]

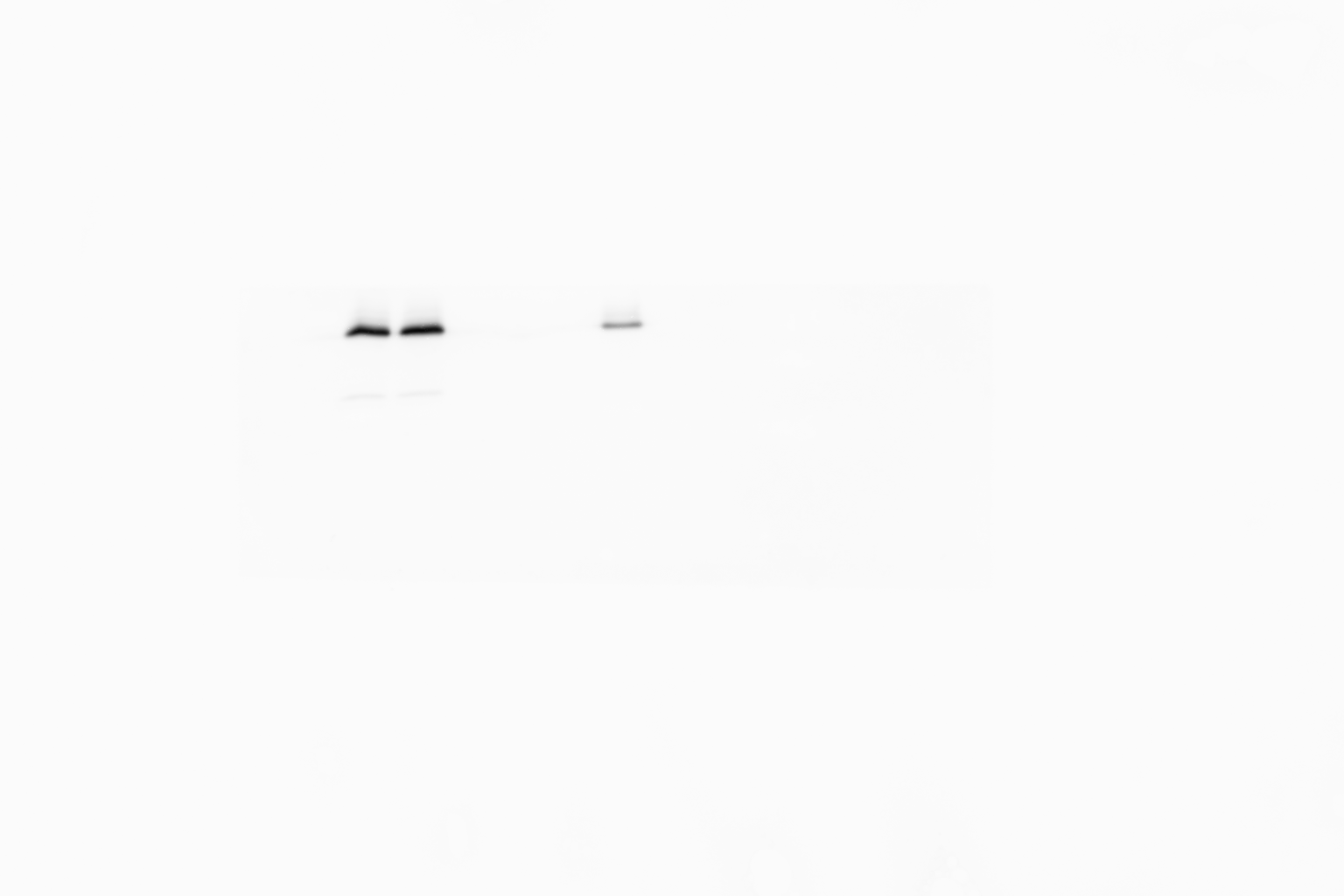

Supplement: Supplementary file 5 — Source Data for Figure 5 [file EMMM-13-e13787-s005.zip › EMM-2020-13787-V2_SourceDataForFigure5D/04032021_GOSR2.tif]

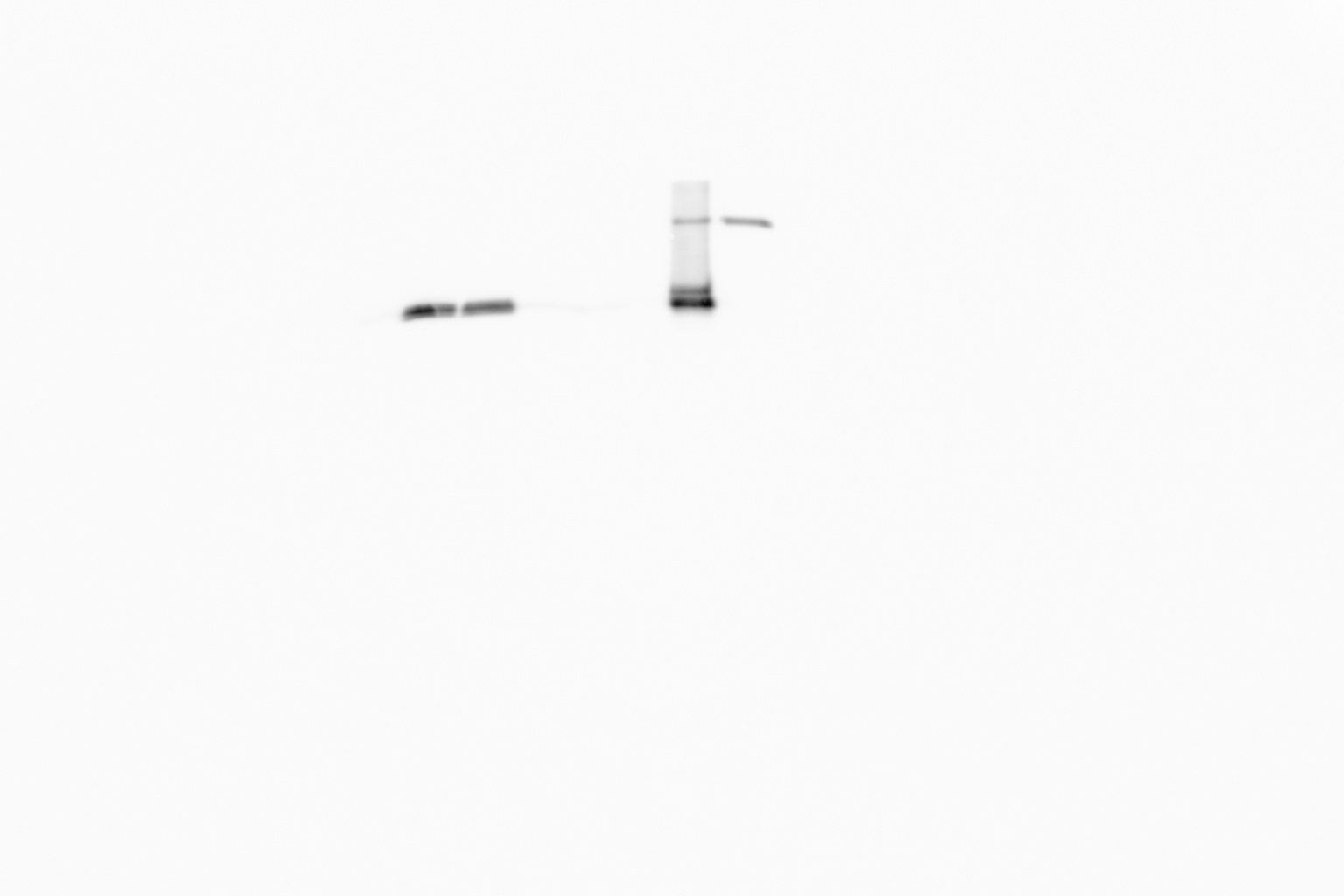

Supplement: Supplementary file 5 — Source Data for Figure 5 [file EMMM-13-e13787-s005.zip › EMM-2020-13787-V2_SourceDataForFigure5D/03032021_Bet1.tif]

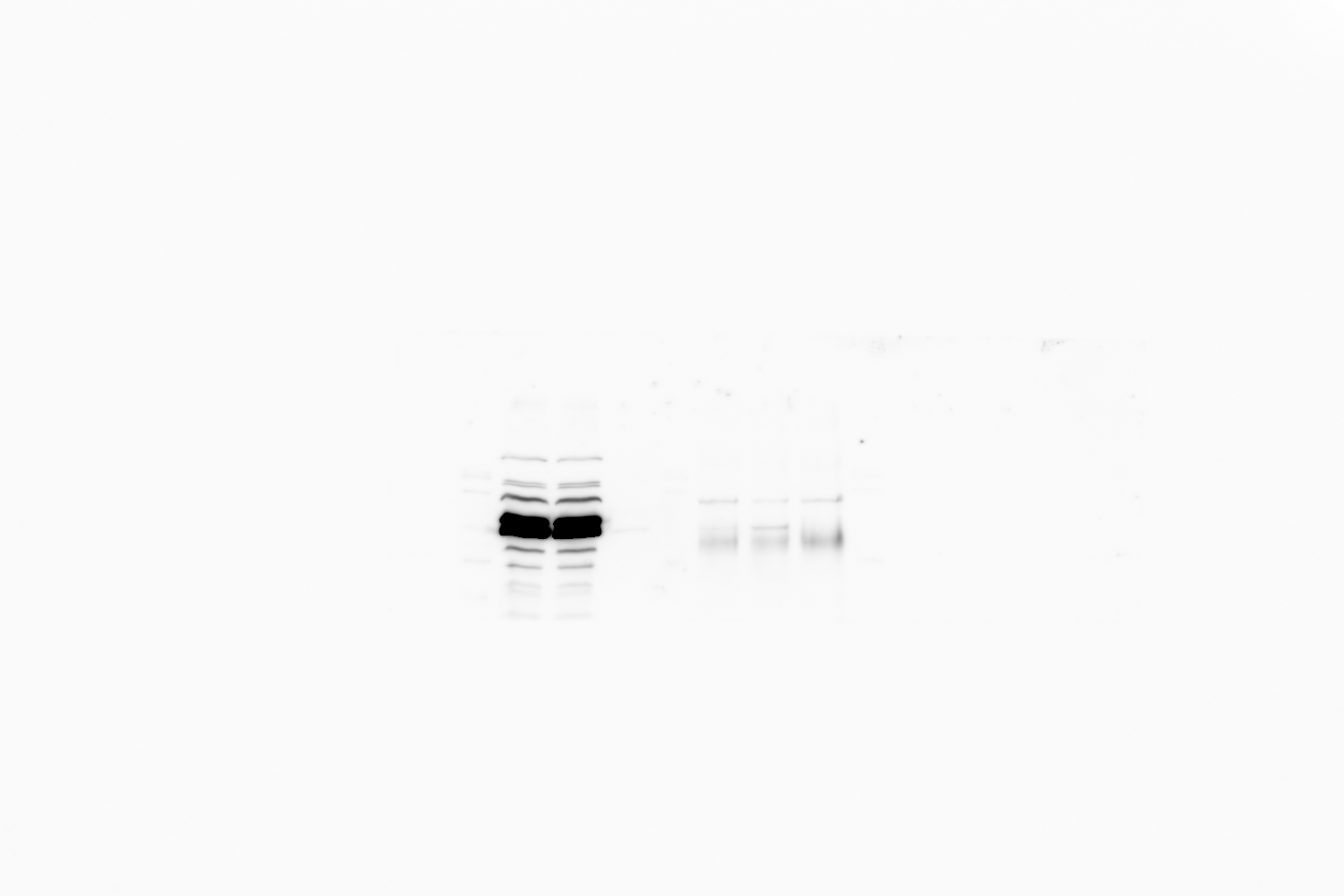

Supplement: Supplementary file 5 — Source Data for Figure 5 [file EMMM-13-e13787-s005.zip › EMM-2020-13787-V2_SourceDataForFigure5D/20210303_ERGIC53.tif]
