# Supplementary material for: BET1 variants establish impaired vesicular transport as a cause for muscular dystrophy with epilepsy
Source: EMBO Mol Med. 2021 Nov 15;13(12):e13787. doi: 10.15252/emmm.202013787 (PMC8649873; doi:10.15252/emmm.202013787)
Supplement: Supplementary file 6 [file EMMM-13-e13787-s004.pdf]

EMBO Molecular Medicine

BET1 variants establish impaired vesicular transport as a cause for muscular dystrophy with epilepsy

Sandra Donkervoort, Niklas Krause, Mykola Dergai, Pomi Yun, Judith Koliwer, Svetlana Gorokhova, Janelle Geist Hauserman, Beryl Cummings, Ying Hu, Rosemarie Smith, Prech Uapinyoying, Partha Ghosh, Vijay Ganesh, Kristin Monaghan, Seby Edassery, Pia Ferle, Sarah Silverstein, Katherine Chao, Molly Snyder, Sara Ellingwood, Diana Bharucha-Goebel, Susan Iannaccone, Matteo Dal Perraro, A. Reghan Foley, Jeffrey Savas, Véronique Bolduc, Dirk Fasshauer, Carsten Bönnemann, and Michael Schwake
DOI: 10.15252/emmm.202013787

Corresponding author(s): Michael Schwake (michael.schwake@uni-bielefeld.de), Carsten Bönnemann (carsten.bonnemann@nih.gov)

Review Timeline:

Submission Date:	4th Dec 20
Editorial Decision:	20th Jan 21
Revision Received:	11th Aug 21
Editorial Decision:	9th Sep 21
Revision Received:	15th Oct 21
Accepted:	19th Oct 21

Editor: Jingyi Hou

Transaction Report:

(Note: With the exception of the correction of typographical or spelling errors that could be a source of ambiguity, letters and reports are not edited. Depending on transfer agreements, referee reports obtained elsewhere may or may not be included in this compilation. Referee reports are anonymous unless the Referee chooses to sign their reports.)

1st Editorial Decision **20th Jan 2021**

Thank you for the submission of your manuscript to EMBO Molecular Medicine. We have now received feedback from two of the three referees whom we asked to evaluate your manuscript. Unfortunately, we did not manage to obtain a report from Referee #2. In the interest of time, I prefer to make a decision now rather than further delaying the process. As you will see from the reports below, the referees acknowledge the potential interest of the study. However, they also raise substantial concerns about your work, which should be convincingly addressed in a major revision of the present manuscript.

I think that the referees' recommendations are rather clear and there is no need to reiterate their comment. In particular, the referees have shared overlapping concerns with regard to the adequacy of the model system (an overexpression approach in a cell type that is not directly relevant to the disease). Referee #1 also mentioned that the major weakness of the study is that there is insufficient evidence to support the causality of the proposed link between the BET1 variants and the pathogenesis of the disease. We think that addressing these concerns is important and would indeed significantly strengthen the manuscript.

All other issues raised by the referees need to be satisfactorily addressed as well. I understand that some of the issues raised by the reviewers require extensive additional analyses and we would be happy to extend the revision deadline, should you need additional time beyond the 90 days. Please note that EMBO Molecular Medicine strongly supports a single round of revision and that, as acceptance or rejection of the manuscript will depend on another round of review, your responses should be as complete as possible.

EMBO Molecular Medicine has a "scooping protection" policy, whereby similar findings that are published by others during review or revision are not a criterion for rejection. Should you decide to submit a revised version, I do ask that you get in touch after three months if you have not completed it, to update us on the status.

We are aware that many laboratories cannot function at full efficiency during the current COVID-19/SARS-CoV-2 pandemic and have therefore extended our "scooping protection policy" to cover the period required for a full revision to address the experimental issues. Please let me know should you need additional time, and also if you see a paper with related content published elsewhere.

***** Reviewer's comments *****

Referee #1 (Comments on Novelty/Model System for Author):

The authors employ the following models:
Yeast complementation: The genes are reasonably conserved and this is a valid approach.
Co-immunoprecipitation and mass spectrometry: This has identified altered protein - protein interactions however the relevance of these in the context of disease is only speculative. The interactions are occurring in a non-muscle and non-neuronal cell type under the conditions of over expression. While there are good technical reasons to do these experiments this way it is an artificial biological system. These data were not validated by co-IP of the endogenous genes in a disease relevant cell type. The authors have patient fibroblasts therefore it is feasible to convert these to myoblasts and immature myotubes which would strengthen these results.
The use of the American College of Medical Genetics and Genomic (ACMG) standards and guidelines for interpretation of sequence variants category PS3 "Well established in vitro or in vivo functional studies are supportive of a damaging effect on the gene or gene product" in Table EV1 is questionable in that the functional data as collected do not directly explain the phenotype. For example, demonstrating that these mutants directly contribute to loss of a-dystroglycan (a known cause of muscular dystrophy) would be valid use of PS3.

Referee #1 (Remarks for Author):

Donkervoort, Krause, et al. have presented a clinical and genetic report of two families where recessive variants in BET1 are implicated in causing a novel, severe congenital muscular dystrophy and epilepsy syndrome. One individual carried two different compound heterozygous variants confirmed to be inherited from each parent by segregation analyses using Sanger sequencing. Two of three identified BET1 variants are present at very low frequencies in the genome aggregation database (gnomAD) while the remaining variant NM_005868.6:c.202G>C has not been previously seen. The authors analyse the transcriptome of the individual with the compound heterozygous variants and show that collectively these alleles lead to a significant loss of transcript and protein expression. In the second family, with a homozygous missense p.Ile51Ser variant that does not affect protein expression, the authors show that this variant affects interaction between ERGIC-53 and BET1 while the core interactions of the SNARE complex of GOSR2, SEC22B, STX5 and BET1 appear to be not disrupted by Co-IP. Using colocalisation analyses of GM130 and ERGIC-53 patient-derived cell lines detected by indirect immunofluorescent staining the authors suggest that mislocalisation of ERGIC-53 could be a general feature of the disorder. The paper is neatly presented and well written by experts in this area. The main weakness of this paper is that there is not much support for BET1 to be intolerant to loss of function thus while the functional data presented show these variants affect gene expression and/or protein-protein interaction it is hard to be certain these variants are truly disease causing.

Major Questions (in addition to those stated above):
1. The RNA-seq data of P1 requires a clearer explanation. It is logical that the majority of restored transcript following CHX treatment is the transcript containing the NM_005868.6:c.134delC; p.(A45Vfs*2) variant however this is not the case as described, with only 33% of reads mapping the wild type allele at position c.202. This is at odds with the Sanger sequencing data shown in EV1C where it looks to be closer to 50% (though this may also be due to the primers not capturing all transcripts seen by RNA-seq). It would be good to genotype these RNA-seq data using a more rigorous method than IGV, which only gives the pileup from a sample of reads (default = 100). Should it be that the majority of RNA-seq reads do contain the mutant

c.202G>C allele, how do the authors explain the large restoration of usage of the splice site between exons 3 and 4 of NM_005868 following CHX treatment. The majority of transcripts using this site terminate in exon 4 and should not be subject to NMD?

2. The results of the loss of interaction between BET1 p.Ile51Ser and ERGIC-53 (LMAN1) are interesting but it is not clear why this one interaction was selected for further follow up. The list of significant interactions identified from AP-MS data appear not to be shown except for a limited excerpt in Figure 5. How many top candidates were found in these analyses? What criteria were used to identify the top candidates? It is difficult to agree with the authors claims of the importance of this interaction in the pathogenesis of the disease when the full story is not shown and the results are derived from a non-muscle cell type.

Minor Questions:

3. Variants given as cDNA coordinates need a version for reference transcript e.g. NM_005868.6
4. Page 8 ...variant was 33/32 in WES and 16/18 in genome sequencing (WS) for... check abbreviations "WES" and "WS" already defined (twice) on page 7 as "ES" and "GS".
5. Page 12: ...localization of I51S mutant BET1 is... Suggest Ile51Ser rather than I51S to maintain consistency.
6. Page 16: ...aggregation and alignment (Bwa-Mem). Should be "BWA-MEM" because it is an acronym for Burrows-Wheeler Aligner Minimal Exact Matches.
7. Page 17: SDS Pages and Immunoblotting. Check grammar: "SDS-PAGE"
8. Page 18: ...was performed with the coloc test of the software ImagJ. Check spelling of "ImageJ"
9. Figure 3 and 5: Microscope images do not have scale bars.
10. Figure 5C: This gel is of poor quality the authors should consider repeating this to present their data in the best way.
11. The primers and methods for RT-PCR and Sanger sequencing shown in the expanded view do not appear to have been included in the manuscript. This should be added.
12. There appears to be no method provided for the molecular dynamic simulations. This should be added.
13. Data availability statement only covers the variant information submitted to ClinVar however the authors have also performed RNA-seq and AP-MS analyses both of which may be of further interest and value to some researchers. There can be very good reasons why these data should have controlled access however the authors should provide some guidance as to how a potential collaborator should approach them.

Referee #3 (Comments on Novelty/Model System for Author):

As outlined in the review below, the use of transfected HEK cells is questionable as it can lead to artefacts. More data should be obtained in the muscle biopsies or in fibroblast-derived muscle cells, at least to confirm the findings in the other systems.

Referee #3 (Remarks for Author):

The manuscript "BET1 variants establish impaired vesicular transport as a cause for muscular dystrophy with epilepsy" by Donkervoort et al report three individuals of two independent families with biallelic variants in the SNARE protein BET1. Mutations cause concomitant congenital muscular dystrophy (CMD) in these individuals and progressive epilepsy in one of the patients.

BET1 is part of a SNARE complex that also contains the SNARE proteins GOSR2, syntaxin-5 and Sec22b. This complex mediates ER to Golgi trafficking. While biallelic variants in GOSR2 have been shown to cause myoclonus epilepsy and ataxia and CMD in one individual, the identification of BET1 as a player in CMD is novel. In addition, having now two genes in the same cellular trafficking pathway linked to CMD may point to an emerging role in this trafficking pathway for CMD. This is important as 50% of patients with CMD remain currently without a confirmed diagnosis.

However, the manuscript falls overall short on several aspects. In particular, it appears that many experiments were done only once, lacking quantifications and thus making many claims unsubstantiated. Also, the title does not reflect the findings of the paper as "impaired vesicular transport" has not been shown.

In addition, the following points should be addressed:

Figure 1A: It is unclear what is shown here. Please label the panels and highlight the findings with arrows to point out the described dysfunction. Also, it would be useful to compare the findings to a WT control, to better highlight the dysfunction in the muscle tissue.

Figure 1B: Please clarify why there is a focus on alpha-dystroglycan, and are there any changes in the glycosylation pattern given the described trafficking deficits and hypoglycosylation for a CMD subset (a better blot should be provided)? Please add quantifications, normalized to GAPDH levels.

Figure 1C: Similar to panel A, a WT control would be helpful to highlight the described differences. If that is not possible, please highlight the described findings with arrows.

Table EV1: I suggest to extend Table EV1 and include a summary of also the clinical findings for the three affected individuals to better highlight similarities and differences in their symptoms. From the current Table EV1 it is also not clear which patient carries which mutation. Please also define AF and CADD in the table legend.

It may be useful to briefly mention that the missense mutations are all found in the SNARE domain which interacts with the SNARE domains of GOSR2, syntaxin-5 and Sec22b. Please also explain TM in the figure legend to Fig. 2A.

Figure 2B: It is unclear what is shown here. Please clarify.

Figure 3A: How many times was this done? Please add quantifications, including levels for the other SNARE proteins syntaxin-5 and Sec22b.

Figure 3B/C: Please quantify the amount of co-localization also for P1. Adding the other SNARE proteins syntaxin-5 and Sec22B in addition to markers for ER and Golgi would be useful to better demonstrate the altered subcellular localization of BET1.

Figure 5A: It is unclear why the mass spec analysis was not done for D68H, and why the interactor screen was performed in transfected HEK293T cells and not patient fibroblasts with endogenous protein levels. Expression levels differ for the mutants in fibroblasts versus HEK cells due to the overexpression approach, and overexpression itself may give rise to false positives.

Figure 5B: It is unclear what is input and what is IP'd sample (also, the labels are off). How many times was this done? Can these data be quantified? Last, it is unclear what the control is since no IP procedure is described in the methods section. A control including antibody and beads with mock-transfected cells would be ideal since SNARE proteins are very sticky and prone to bind non-specifically to beads. If possible, please also add syntaxin-5 blots. Last, it is unclear why more Sec22b seemed to have been pulled down, given that Figure 4 predicts a reduction in binding of D68H for Sec22b. Could Bet1-HA just be pulling down ERGIC because it is overexpressed and therefore localizing incorrectly?

Figure 5C: How many times was this done? Please add quantifications. It would be useful to test for changes in protein levels and SNARE complex levels also in muscle biopsy samples that are available to the authors to link their findings to impairments in muscle.

Figure 5D-F: It is unclear why the overlap of P1 with GM130 and PD1 is not shown. The shape of the Golgi looks slightly different in P2 than control - can this be quantified? Given these are the key SNAREs in protein trafficking, it is important to perform a trafficking assay to demonstrate if this increase in GM130 and ERGIC co-localization has functional changes, in particular since the title of this manuscript mentions impaired vesicle transport.

It is possible to induce muscle from fibroblasts. Given that the symptoms come from muscle, which differentiates it from the GOSR2 phenotype, it would be interesting to see if there is any difference between fibroblasts and muscle, at least for a few readouts.

EV2: Please add D68H here as well.

Minor points:

Are the errors shown SD or SEM?

It would be useful to refer to everything by mutation and not switch between P1 and P2 all the time.

Referee #1 (Comments on Novelty/Model System for Author):

The authors employ the following models:
Yeast complementation: The genes are reasonably conserved and this is a valid approach.
Co-immunoprecipitation and mass spectrometry: This has identified altered protein - protein interactions however the relevance of these in the context of disease is only speculative. The interactions are occurring in a non-muscle and non-neuronal cell type under the conditions of over expression. While there are good technical reasons to do these experiments this way it is an artificial biological system. These data were not validated by co-IP of the endogenous genes in a disease relevant cell type. The authors have patient fibroblasts therefore it is feasible to convert these to myoblasts and immature myotubes which would strengthen these results.

The use of the American College of Medical Genetics and Genomic (ACMG) standards and guidelines for interpretation of sequence variants category PS3 "Well established in vitro or in vivo functional studies are supportive of a damaging effect on the gene or gene product" in Table EV1 is questionable in that the functional data as collected do not directly explain the phenotype. For example, demonstrating that these mutants directly contribute to loss of a-dystroglycan (a known cause of muscular dystrophy) would be valid use of PS3.

Referee #1 (Remarks for Author):
Donkervoort, Krause, et al. have presented a clinical and genetic report of two families where recessive variants in BET1 are implicated in causing a novel, severe congenital muscular dystrophy and epilepsy syndrome. One individual carried two different compound heterozygous variants confirmed to be inherited from each parent by segregation analyses using Sanger sequencing. Two of three identified BET1 variants are present at very low frequencies in the genome aggregation database (gnomAD) while the remaining variant NM_005868.6:c.202G>C has not been previously seen. The authors analyse the transcriptome of the individual with the compound heterozygous variants and show that collectively these alleles lead to a significant loss of transcript and protein expression. In the second family, with a homozygous missense p.Ile51Ser variant that does not affect protein expression, the authors show that this variant affects interaction between ERGIC-53 and BET1 while the core interactions of the SNARE complex of GOSR2, SEC22B, STX5 and BET1 appear to be not disrupted by Co-IP. Using colocalisation analyses of GM130 and ERGIC-53 patient-derived cell lines detected by indirect immunofluorescent staining the authors suggest that mislocalisation of ERGIC-53 could be a general feature of the disorder. The paper is neatly presented and well written by experts in this area. The main weakness of this paper is that there is not much support for BET1 to be intolerant to loss of function thus while the functional data presented show these variants affect gene expression and/or protein-protein interaction it is hard to be certain these variants are truly disease causing.

> We thank reviewer 1 for the constructive feedback. In this work we provide detailed clinical data on three patients from two independent families in whom rare biallelic *BET1* variants were identified. Given some of the clinical similarities with patients carrying pathogenic variants in other SNARE complex partner genes, we decided to pursue further validation work as disease causation for *BET1* appeared reasonable to postulate. The effects of these variants were evaluated in patient muscle through RNA sequencing, patient fibroblasts and accessible model systems. We now also show that knockdown of *BET1* results in impaired ER-to-Golgi trafficking in cells and thus provide some initial mechanistic insights in the role of BET1 in normal and impaired vesicular transport, and establish its role in early-onset neuromuscular disease.

In re-review of the ACMG standards we noted that the ACMG classification can only be applied for genes with confirmed pathology-gene association. At this time there is no known disease associated for *BET1* variants, as this report will be the first. All *BET1* variants therefore remain classified as a variant of unknown significance until disease pathogenicity has been established through publication of this report. To avoid confusion, we have removed the ACMG classification from the table.

We agree that additional analyses in muscle tissue would be informative to further understand the underlying muscle pathogenesis but unfortunately there was no additional muscle tissue available from our patients for such further testing. We hope that this will be an option once additional patients with BET1-related disease have been identified.

Instead, we then tried to explore the use of Human Skeletal Muscle Myoblasts (HSMMs; Lonza) as a model system for further testing of *BET1* knockdown. Unfortunately, however, we were unsuccessful in three replicate experiments to achieve meaningful knockdown of *BET1* using shRNA delivered by lentivirus at various titers. Part of the problem was the decidedly mixed nature of the commercially available line: The HSMMs were characterized by the vendor to be >90% desmin positive as an indication of skeletal muscle lineage, however, passages tested in our lab revealed overall low levels of desmin expression. These low levels of desmin expression led us to believe that the HSMMs are a mixture of myoblasts and fibroblasts so that given the high expression of BET1 in fibroblasts compared to myoblasts, we were unable to determine whether a BET1 signal was specific to myoblasts in these cells.

For future work we are interested in establishing iPSC induced myoblasts from the patients and controls and in creating an appropriate animal model.

Major Questions (in addition to those stated above):
1a. The RNA-seq data of P1 requires a clearer explanation

> We agree with the reviewer that the results and conclusions from our RNA data needed more clarification. To address this issue, we have completely re-analyzed the RNAseq data focusing on the patient derived muscle and complemented this with additional RT-PCR experiments. Moreover, given the significant quantitative differences in *BET1* expression between muscle tissue and cultured fibroblasts (apparent in our results, as well as GTEx data), we believe that for the transcript analysis the main conclusions should be drawn as much as possible from the muscle RNAseq data only, while using the fibroblast data as supporting evidence.

In the original manuscript we had assumed that the paternal allele is degraded by NMD since the c.202G>C variant was barely detectable by the initial RNAseq analysis, hence we based everything on the fibroblasts analysis, including the CHX treatment experiments. However, from the new analysis of the patient muscle RNA seq data an allele balance between the maternal and paternal alleles was less obvious when all aberrantly spliced reads from the paternal allele are fully taken into account. In the original RT-PCR experiments, the reverse oligo was placed in exon 4, which left the transcript that skips exon 4 undetectable. We pursued additional RT-PCR experiments, this time using primers placed in *BET1* exon 2 extending to 3’UTR

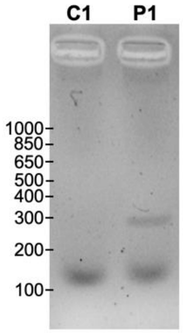

RT-PCR using a primer in *BET1* exon 2 and exon 5 of non-coding transcript NR_133909.2. In P1 we were able to show presence of exon 5, while this was absent in control.

encoding exon 5 of *BET1* transcript NR_133909.2.
(Forward Primer 5'- CTC CTG GCA ACT ATG GGA AC - 3'
Reverse Primer 5' - TGC AGC TGT GAT AGA TGG CA - 3')

The results obtained were consistent with the findings that were evident on RNA sequencing, with shows the presence of exon 5 in P1's fibroblasts. As expected, expression of exon 5 was not observed in the control fibroblast. In light of these new findings, our CHX-treatment was not needed and is no longer included in this new version of the manuscript. We are not sure what other indirect effects to CHX might have influenced transcript levels and patterns in our initial experiment in the fibroblasts.

We have now completely re-written the relevant Result section describing this point and made significant changes to the Figure 2. We believe that these modifications provide a better explanation for the RNAseq data and should now fully answer the reviewer's concerns.

*1b. It is logical that the majority of restored transcript following CHX treatment is the transcript containing the NM_005868.6:c.134delC; p.(A45Vfs*2) variant however this is not the case as described.*

> As requested by the reviewer, we have re-analyzed the RNAseq data and completely re-wrote the corresponding section of the manuscript. As described above, the CHX treatment data is no longer relevant to our manuscript since the new results from patient muscle RNAseq suggest that there is no imbalance in the allele expression from *BET1* locus in muscle tissue from P1. Because of the significantly higher expression of BET1 in fibroblasts and unknown impact of CHX on transcript levels and splicing patterns, we opted to focus on muscle RNA sequencing, specifically as it is a disease relevant tissue source.

1c. This is at odds with the Sanger sequencing data shown in EV1C where it looks to be closer to 50% (though this may also be due to the primers not capturing all transcripts seen by RNA-seq).

> Indeed, the initial RT-PCR experiments did not capture the transcript with skipped exon 4, since the reverse oligo was located in the exon 4. In the revised manuscript, we used an additional primer located in the downstream exon to further explore the *BET1* transcripts by RT-PCR (see previous point).

1d. It would be good to genotype these RNA-seq data using a more rigorous method than IGV, which only gives the pileup from a sample of reads (default = 100). Should it be that the majority of RNA-seq reads do contain the mutant c.202G>C allele, how do the authors explain the large restoration of usage of the splice site between exons 3 and 4 of NM_005868 following CHX treatment.

> As outlined earlier, we believe that the main conclusions about the effects of *BET1* variants on the mRNA expression at this locus is best based on the more physiologically relevant muscle RNA analysis rather than the fibroblasts RNA analysis we relied on previously. Indeed, the expression of BET1 is much higher in fibroblasts, as is evident from our results as well as from GTEx data, and RNA processing and stability may also be different. The CHX treatment data we generated on fibroblasts are thus no longer relevant to our manuscript since the re-analysis of the muscle RNAseq data showed no imbalance in the allele expression from *BET1* locus. The number of reads covering the *BET1* variants in the muscle RNA seq data is

lower than 100, so the analysis did not utilize downsampling through IGV. These conclusions are now also confirmed by additional RT-PCR experiments.

For the scope of our study, the recognition of the pleotropic effect of this missense variant on splicing is fundamental, as it provides insights in our subsequent strategies to elucidate pathogenicity, as one could postulate both a loss of protein, in addition to, mutant BET1 as a driver of disease mechanism.

2. The results of the loss of interaction between BET1 p.Ile51Ser and ERGIC-53 (LMAN1) are interesting but it is not clear why this one interaction was selected for further follow up. The list of significant interactions identified from AP-MS data appear not to be shown except for a limited excerpt in Figure 5. How many top candidates were found in these analyses? What criteria were used to identify the top candidates? It is difficult to agree with the authors claims of the importance of this interaction in the pathogenesis of the disease when the full story is not shown and the results are derived from a non-muscle cell type.

> To address this concern, we have data sets from three independent experiments. We have identified peptides from a total of roughly 11.000 proteins. To analyze these data, we used spectral counting as a label free quantitative approach. We excluded all spectral counts present in the mock transfected AP-MS samples, since these represent proteins unspecifically bound to the matrix used for precipitation. As a cut off, we used the spectral counting data for BET1 found in mock controls (max. two spectral counts). By applying these criteria, we identified known BET1 interactors such as GOSR2, YKT6 and GOSR1 and several other SNAREs, which were also found in the BET1 I51S samples, strongly suggesting that this particular variant, under these conditions, is not interfering with SNARE interaction. However, we also identified sixteen proteins only in the BET1 wt but not in the I51S variant samples, including our top candidate ERGIC-53, followed by other proteins such as the translation initiation factor EIF4ENIF1, ubiquilin-1, etc. Since ERGIC-53 and BET1 reside in the same cellular compartment, we validated the AP-MS data by co-ip studies (see points 9 and 10 raised by reviewer 3). The list of BET1 interaction partners is now shown as a supplementary table and presented as a volcano blot in Figure 5A and EV2E.

Minor Questions:
3. Variants given as cDNA coordinates need a version for reference transcript e.g. NM_005868.6

> We apologize for the oversight and have added the complete reference transcript to the result section on page 8.

4. Page 8 ...variant was 33/32 in WES and 16/18 in genome sequencing (WS) for... check abbreviations "WES" and "WS" already defined (twice) on page 7 as "ES" and "GS".

> Thank you for bringing this to our attention. We had updated the manuscript accordingly.

5. Page 12: ...localization of I51S mutant BET1 is... Suggest Ile51Ser rather than I51S to maintain consistency.

> Thank you for this suggestion - We have updated the manuscript accordingly.

6. Page 16: ...aggregation and alignment (Bwa-Mem). Should be "BWA-MEM" because it is an acronym for Burrows-Wheeler Aligner Minimal Exact Matches.

> Thank you - We had updated page 20 of the manuscript accordingly.

7. Page 17: SDS Pages and Immunoblotting. Check grammar: "SDS-PAGE"

> Thank you again - we have updated page 22 of the manuscript accordingly.

8. Page 18: ...was performed with the coloc test of the software ImagJ. Check spelling of "ImageJ"

> Thank you - corrected.

9. Figure 3 and 5: Microscope images do not have scale bars.

> We apologize for the oversight and have now added scale bars to all microscope images.

10. Figure 5C: This gel is of poor quality the authors should consider repeating this to present their data in the best way.

> Thank you for the suggestion, we have repeated the experiment and included the updated gel in Figure 3A.

11. The primers and methods for RT-PCR and Sanger sequencing shown in the expanded view do not appear to have been included in the manuscript. This should be added.

> We apologize for this oversight; this has now been added to the methods.

12. There appears to be no method provided for the molecular dynamic simulations. This should be added.

> We apologize for not providing a description of the molecular dynamic simulations in the initial submission of our manuscript. This can now be found in the revised version of the manuscript in the experimental procedures section on page 25.

13. Data availability statement only covers the variant information submitted to ClinVar however the authors have also performed RNA-seq and AP-MS analyses both of which may be of further interest and value to some researchers. There can be very good reasons why these data should have controlled access however the authors should provide some guidance as to how a potential collaborator should approach them.

> Thank you for bringing this to our attention. We have updated the data availability statement on page 30 to now include:

All data generated in this study are available upon request from the authors. All requests will be reviewed by the respective institution to verify if the request is subject to any intellectual property or confidentiality obligations. Raw sequence data for P1 is uploaded to AnVIL (https://anvilproject.org/data/). Data access applications are through dbGaP (http://www.ncbi.nlm.nih.gov/gap) Study Accession phs001272.v1.p1. Data in this manuscript can be found in project AnVIL_CMG_Broad_Muscle_Bonnemann_WES. Phenotype data and candidate gene/variant information are shared through *Seqr* with the Matchmaker Exchange system (https://www.matchmakerexchange.org/). All rare variants are

also uploaded to Geno2MP (http://geno2mp.gs.washington.edu), which is a web-based query tool that searches a database of rare variants from exome sequencing data linked to phenotypic information from a wide variety of Mendelian gene discovery projects like the CMGs. Variants reported here are available in ClinVar (https://www.ncbi.nlm.nih.gov/clinvar/ identifier 619194 (c.202G>C), 619186 (c.134delC) and 977650 (c.152T>G)).

Referee #3
(Comments on Novelty/Model System for Author):
As outlined in the review below, the use of transfected HEK cells is questionable as it can lead to artefacts. More data should be obtained in the muscle biopsies or in fibroblast-derived muscle cells, at least to confirm the findings in the other systems.

Referee #3 (Remarks for Author):
The manuscript "BET1 variants establish impaired vesicular transport as a cause for muscular dystrophy with epilepsy" by Donkervoort et al report three individuals of two independent families with biallelic variants in the SNARE protein BET1. Mutations cause concomitant congenital muscular dystrophy (CMD) in these individuals and progressive epilepsy in one of the patients.

BET1 is part of a SNARE complex that also contains the SNARE proteins GOSR2, syntaxin-5 and Sec22b. This complex mediates ER to Golgi trafficking. While biallelic variants in GOSR2 have been shown to cause myoclonus epilepsy and ataxia and CMD in one individual, the identification of BET1 as a player in CMD is novel. In addition, having now two genes in the same cellular trafficking pathway linked to CMD may point to an emerging role in this trafficking pathway for CMD. This is important as 50% of patients with CMD remain currently without a confirmed diagnosis.

However, the manuscript falls overall short on several aspects. In particular, it appears that many experiments were done only once, lacking quantifications and thus making many claims unsubstantiated. Also, the title does not reflect the findings of the paper as "impaired vesicular transport" has not been shown.

> We thank this reviewer for the constructive criticism, which we fully accept. In the revised manuscript we repeated all key experiments at least three times and added quantifications. We also provide strong evidence that down-regulation of *BET1* by siRNA interferes with ER-to-Golgi transport. We feel that the message of the paper is now strengthened.

In addition, the following points should be addressed:

1. Figure 1A: It is unclear what is shown here. Please label the panels and highlight the findings with arrows to point out the described dysfunction. Also, it would be useful to compare the findings to a WT control, to better highlight the dysfunction in the muscle tissue.

> We would like to thank the reviewer for the suggestion. We have updated Figure 1A to now include an unaffected control (1A iv-vi) to better highlight the muscle involvement in P1. In addition, we added arrows and updated the legend accordingly to include:

‘Left quadriceps muscle biopsy performed at age 10 months (i-iii) demonstrating a dystrophic process with variation in fiber size (black arrow), increased connective tissue (blue arrow) and rare internalized nuclei (white arrow) on Hematoxylin and eosin (H&E) staining (i) and

Gömöri trichrome staining (ii) and type 1 fiber atrophy on nicotinamide adenine dinucleotide (NADH) staining (iii). Muscle biopsy in an unaffected (control) individual (iv-vi).'

2. Figure 1B: Please clarify why there is a focus on alpha-dystroglycan, and are there any changes in the glycosylation pattern given the described trafficking deficits and hypoglycosylation for a CMD subset (a better blot should be provided)? Please add quantifications, normalized to GAPDH levels.

> Based on the clinical presentation of early onset progressive weakness, with respiratory involvement, cataracts, seizures, and elevated creatine kinase a form of alpha-dystroglycanopathy was prominently part of the patient's differential diagnosis. A recent study linked hypoglycosylation of the α-dystroglycan matriglycan in patients with impaired vesicular transport due to pathogenic variants in *TRAPPC11* and *GOSR2 (Larson et al, 2018).* In this study we were able to show this now for BET1 in our patient as well, importantly in muscle, but less so in fibroblasts, in that we observed a reduction in the alpha-dystroglycan matriglycan glycoepitope (updated Figure 1C). While this reduction is not to the extend as patients with a severe primary alpha-dystroglycanopathy it suggests that impaired glycosylation of α-dystroglycan may be part of disease mechanisms at play in BET1-related disease that are tied to the impaired vesicle transport due to BET1 dysfunction.

We have now added quantification data for the WB in Figure 1B. The 'smear' evident on the blot is an expected finding and reflects the variably sized glycoepitope of alpha-dystroglycan as recognized by this matriglycan glycoepitope specific antibody. We were also able to analyze glycosylated alpha-dystroglycan levels in fibroblasts from P1 and P2. Glycosylated alpha-dystroglycan levels in P1 and P2 fibroblasts did not show a significant reduction, as observed in fibroblasts from a patient with a confirmed alpha-dystroglycanopathy due to biallelic pathogenic variants in *LARGE* that we included as a disease control. We updated the methods (page 22) and results (page 4 & 5) accordingly.

3. Figure 1C: Similar to panel A, a WT control would be helpful to highlight the descried differences. If that is not possible, please highlight the described findings with arrows.

> We would like to thank the reviewer for the suggestion. We have updated Figure 1C to now include an unaffected control (bottom) and arrow to better illustrate the brain findings in P1. We have updated the legend accordingly.

'Brain MRI findings in P1 at 28 months of age (top) revealing normal appearing cortex, cerebellum and pons on T1-weighted images and T2-weighted image. There is evidence of mild thinning of the corpus callosum (arrow). Brain MRI in an unaffected (control) individual at 30 months of age (bottom) demonstrating normal sized corpus callosum.'

4. Table EV1: I suggest to extend Table EV1 and include a summary of also the clinical findings for the three affected individuals to better highlight similarities and differences in their symptoms. From the current Table EV1 it is also not clear which patient carries which mutation. Please also define AF and CADD in the table legend.

> We would like to thank the reviewer for the suggestions. We have updated the table and legend accordingly.

5. It may be useful to briefly mention that the missense mutations are all found in the SNARE domain which interacts with the SNARE domains of GOSR2, syntaxin-5 and Sec22b. Please also explain TM in the figure legend to Fig. 2A.

> Thank you for the suggestion, which we have incorporated in page 8 of the manuscript. The identified *BET1* missense variants impact the conserved SNARE domain. Interestingly, based on muscle RNA sequencing we were able to show that the c.202G>C; p.Asp68His acts as a complex hypomorph splice variant resulting in three different splice variants (Figure 2B and C). Two of the splice variants are predicted to result in an unstable product, while the third splice variant generates a full-length Asp68His product. Indeed, the molecular dynamic (MD) simulations shows that Asp68 is involved in a highly stable hydrogen bond with Lys169 of SEC22b. Modeling for the second variant, the BET1 Ile51 residue, indicated that the side chain of this residue is localized at the outer surface of the SNARE complex, in close proximity to a highly conserved hydrophobic residue (Ile148) in SEC22. We replaced the abbreviation TM by TMD which stands for transmembrane domain. We have updated the legend in Figure 2A accordingly.

6. Figure 2B: It is unclear what is shown here. Please clarify.

> Figure 2B is a quantitative visualization of the splice junction read alignments alongside the genomic coordinates and gene annotation. The Sashimi plot compares muscle RNA sequencing data from P1 (red top) with three independent controls at the *BET1* locus. We have updated the legend to clarify this figure.

7. Figure 3A: How many times was this done? Please add quantifications, including levels for the other SNARE proteins syntaxin-5 and Sec22b.

> Thank you for bringing this to our attention. We repeated this experiment at least three times. We also probed for Syntaxin-5 and SEC22b and quantified the respective immunoblots. A summary of this quantifications in now shown in the revised EV Figure 3A.

8. Figure 3B/C: Please quantify the amount of co-localization also for P1. Adding the other SNARE proteins syntaxin-5 and Sec22B in addition to markers for ER and Golgi would be useful to better demonstrate the altered subcellular localization of BET1.

> We thank the reviewers for the suggestions. We have completed co-localization and PCC for BET1 with SEC22B and Syntaxin-5 and have updated the manuscript on page 10 and Figure EV1C. In P2 fibroblasts, the colocalization of BET1 with GOSR2 and Syntaxin-5 was reduced compared to the control fibroblasts while colocalization of BET1 with SEC22B was normal. In the overlay BET1 colocalization with GOSR2 and Syntaxin-5 is reduced in P2 compared to controls while BET1 colocalization with SEC22b is normal: (GOSR2 P2: n=56; C: n =86, p= 0.0025), (Syntaxin-5 P2: n=27; C: n =32 p = 0.0006) (SEC22b P2: n=27; C: n =30, p = 0.2093).

Figure EV1C and the corresponding legend have been updated accordingly. We were unable to determine colocalization in fibroblasts from P1 due to the faint BET1 signal caused by the hypomorphic nature of the disease alleles in P1.

9. Figure 5A: It is unclear why the mass spec analysis was not done for D68H, and why the interactor screen was performed in transfected HEK293T cells and not patient fibroblasts

with endogenous protein levels. Expression levels differ for the mutants in fibroblasts versus HEK cells due to the overexpression approach, and overexpression itself may give rise to false positives.

> Based on the RNAseq data, we estimated for the mutated Asp68His BET1 protein to be present at approximately 9% of normal protein level as this allele mostly leads to aberrant splicing and only few transcripts lead to incorporation of the missense variant. This strong reduction in expression was validated by immunoblotting and immunofluorescence, suggesting that this variant is primarily hypomorphic. Therefore, we did not include this variant in the AP-MS analysis. We routinely perform AP-MS analysis using HA-magnetic or GFP-trap beads, which both show low unspecific binding properties. To be able to use these beads, we have to overexpress bait proteins as fusion proteins carrying the desired tags in this case the HA epitope. We agree with the reviewer that this overexpression approach tends to give rise to false positives. However, since we performed comparative experiments with wild type and the I51S variant and both proteins showed comparable expression levels, we are confident that we have minimized technical artefacts (see also next point).

Furthermore, we were able to co-immunoprecipitate BET1 and ERGIC-53 from HEK lysates, making us confident that this particular interaction is not a false positive.

10. Figure 5B: It is unclear what is input and what is IP'd sample (also, the labels are off). How many times was this done? Can these data be quantified? Last, it is unclear what the control is since no IP procedure is described in the methods section. A control including antibody and beads with mock-transfected cells would be ideal since SNARE proteins are very sticky and prone to bind non-specifically to beads. If possible, please also add syntaxin-5 blots. Last, it is unclear why more Sec22b seemed to have been pulled down, given that Figure 4 predicts a reduction in binding of D68H for Sec22b. Could Bet1-HA just be pulling down ERGIC because it is overexpressed and therefore localizing incorrectly?

> To address these points, we relabeled the figure and clearly indicate the input and the IP/Co-IP samples. We repeated these experiments three times, quantified, and combined the quantifications in a diagram now shown in figure 5B and C, including syntaxin 5 data.
We also provide now a detailed description of the IP procedure in the method section on page 29 of the revised manuscript. As suggested by the reviewer, we used mock-transfected cells to control for unspecific binding to beads.

The Co-IP quantifications suggest a non-significant reduction in binding of the D68H mutant protein to SEC22b. However, since our RNA-seq and immunoblotting data strongly suggest that this particular variant acts as a hypomorphic we refrained from performing additional Co-IP studies.

11. Figure 5C: How many times was this done? Please add quantifications. It would be useful to test for changes in protein levels and SNARE complex levels also in muscle biopsy samples that are available to the authors to link their findings to impairments in muscle.

> We thank the reviewer for the suggestion and agree that additional studies in muscle from patients with BET1-related disease would be important. Unfortunately, we do not have additional muscle tissue available at this time for further studies. We hope that this will be an option once additional patients have been identified. We have updated figure 5C to add the quantifications, experiments were done in three independent replicates. We were able to confirm that levels of the SNARE-complex partners were normal in patient’s cells compared

to controls which is now included in Figure EV3A.

12. Figure 5D-F: It is unclear why the overlap of P1 with GM130 and PD1 is not shown. The shape of the Golgi looks slightly different in P2 than control - can this be quantified? Given these are the key SNAREs in protein trafficking, it is important to perform a trafficking assay to demonstrate if this increase in GM130 and ERGIC co-localization has functional changes, in particular since the title of this manuscript mentions impaired vesicle transport.

> Thank you for the suggestions. The quantification of the Golgi area was inconclusive as inconsistencies between P1 and P2 were observed. At this time, we do not think that it provides further insight in disease pathogenesis. We therefore opted not to include this data in the revision.

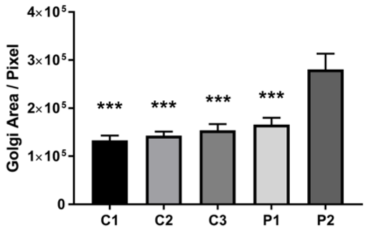

We have now included a siRNA *BET1* knockdown approach in HeLA cells and through a pC4-secretion assay show mislocalization at the cis-Golgi in BET1 deficient cells consistent with impaired vesicular transport. We have updated the manuscript accordingly and included results in Figure 3E. In addition, based on the reviewer's suggestion we have included a BrefeldinA Golgi reconstitution assay which shows slowed reconstitution of the Golgi in patients-derived fibroblasts compared to controls (Figure 5G).

13. It is possible to induce muscle from fibroblasts. Given that the symptoms come from muscle, which differentiates it from the GOSR2 phenotype, it would be interesting to see if there is any difference between fibroblasts and muscle, at least for a few readouts.

> We now include muscle RNA sequencing data which provides a more accurate and disease specific source for analyzing the effect of the missense variant. Unfortunately, we do not have access to additional muscle tissue, but are hoping to obtain further insights in muscle specific involvement in future when additional patients have been identified.

Reviewer #1 had a similar suggestion to analyze muscle specifically and in our response, we pointed out our attempts to do this (see above and repeated here):

"We agree that additional analyses in muscle tissue would be informative to further understand the underlying muscle pathogenesis but unfortunately there was no additional muscle tissue available from our patients for such further testing. We hope that this will be an option once additional patients with BET1-related disease have been identified.

Instead, we then tried to explore the use of Human Skeletal Muscle Myoblasts (HSMMs; Lonza) as a model system for further testing of *BET1* knockdown. Unfortunately, However, we were unsuccessful in three replicate experiments to achieve meaningful knockdown of BET1 using shRNA delivered by lentivirus at various titers. Part of the problem was the decidedly mixed nature of the commercially available line: The HSMMs were characterized by the vendor to be >90% desmin positive as an indication of skeletal muscle lineage, however, passages tested in our lab revealed overall low levels of desmin expression. These low levels of desmin expression led us to believe the HSMMs are a mixture of myoblasts and fibroblasts so that given the high expression of BET1 in fibroblasts compared to myoblasts, we were unable to determine whether a BET1 signal was specific to myoblasts in these cells.

For future work we are interested in establishing iPSC induced myoblasts from the patients and controls and in creating an appropriate animal model.

14. EV2: Please add D68H here as well.

> Since the affected aspartate is not conserved at corresponding position in yeast, we refrain from performing these experiments. This is now stated in the revised version of the manuscript on page 15.

Minor points:
15. Are the errors shown SD or SEM?
> The errors are SD throughout the manuscript.

16. It would be useful to refer to everything by mutation and not switch between P1 and P2 all the time.
> Thank you for the suggestions, we have updated the manuscript to refer to the specific mutation when discussing the validation work.

Thank you for the submission of your revised manuscript to EMBO Molecular Medicine. We have now received the enclosed report from the one referee who agreed to re-assess it. As you will see the referee is now overall supportive and I am pleased to inform you that we will be able to accept your manuscript pending the following amendments:

***** Reviewer's comments *****

Referee #1 (Comments on Novelty/Model System for Author):

Comments to the authors cover the reasons for the categories selected.

Referee #1 (Remarks for Author):

The authors have provided a substantially revised manuscript from their original submission "BET1 variants establish impaired vesicular transport as a cause for muscular dystrophy with epilepsy". New data include an assay to assess ER to Golgi vesicular transport. RNA-seq on patient muscle and a Golgi reconstitution assay. Additionally, the robust quantification of molecular findings that have now been included considerably improve the quality and clarity of the data presented. The study nicely demonstrates the power of patient cell lines to functionally assess pathogenicity of variants, especially when statistical genetics approaches cannot be applied.

I have only a few comments and I have been asked by the editorial team to provide an assessment on the responses to reviewer #3 in their absence.

Major Comments:
1. I'm curious why the attempt to use HSMMs rather than transdifferentiation of the patient fibroblasts to myoblasts which was recommended by both reviewers? Direct transdifferentiation of patient fibroblasts using MYOD is a very well established technique that is faster than iPSC to myoblast transformation. These myoblasts lack the ability to form mature myotubes but they will form immature sarcomere and express genes from the dystrophin associated glycoprotein complex.

2. Page 14: "At this stage the latter results are too preliminary and need further experimentation." What's a reviewer to do with a statement of this nature? I appreciate the honesty, though I'd advise to reword or remove this sentence. Looking at the results provided it seems that the quantification of the interaction between BET1 and SEC22B were highly variable. I agree the result is not significant as stated in the preceding sentence in the manuscript to the one quoted above. The authors should either leave it at that accepting that their experimental design was adequately powered, or carry out the additional experiments to confirm or deny the observation. It is additionally interesting in figure EV1 C that SEC22B appears to colocalise with BET1 in the patient #2 cell line. Does this mean that SEC22B is also mislocalised in patient cells relative to GOSR2 and STX5? I'd note that I'm not insisting this be done but just pointing out the risk the authors may be taking in publishing as is without looking at this further.

3. I believe the major and minor points raised by reviewer #3 have been addressed (other than the remaining question regarding fibroblast transdifferentiation above). There remains a possibility that the over-expression of BET-1 mutants in HEK293T and the AP-MS analysis has pulled out some non-specific interactions however the authors use of control over expression of wild type BET1 and only using the subtraction between wild type and mutant proteins is an acceptable compromise given muscle is now unavailable and a mouse model is beyond the scope of this work. The additional quantification of data and the demonstration of an ER to Golgi trafficking defect using one assay likely satisfy other concerns raised by reviewer #3.

Minor comments:
4. Figure 1C: The abbreviation adg-pt is not defined nor IIH6 (though it is in the methods) alpha Dystroglycan.

5. In Figure 3D, are the immunofluorescence images taken at 10 minutes post induction of secretion? This isn't clear from the figure legend but the data suggests it. Are the numbers of cells quoted "0min: ctrl n=63, #1 n=56, #2 n=64; 10 min: ctrl n=104, #1n=126, #2 n=110." Referring to data in 3E?

6. The authors should consider removing references to individuals being Caucasian unless all of these families indeed came from the Caucasus region of Eastern Europe. Caucasian in reference to race does not have scientific value and though I am certain the authors do not intend this, it is an offensive term to many, e.g. Kaplan JB, Bennett T. Use of Race and Ethnicity in Biomedical Publication. JAMA. 2003 May 28; 289(20):2709-16.

7. The new sections of the manuscript contain some minor grammatical errors. Here are some examples but there may be others I have missed.
Page 13-14: "Among proteins which did not co-precipitated with the Ile51Ser mutant, ..."
Page 16 "...and causes the Golgi to reversible break down into vesiculated Golgi membranes..."
Page 19: "The detrimental effect on vesicular trafficking was subsequently confirmed through down regulation of BET1 by siRNAs resulting in impaired ER-to-Golgi transport (Figure 2B)." Likely this refers to Figure 3D?
Page 23: "...cOmplet mini protease inhibitor cocktail"
Page 40; Figure 3 legend: "Colocalization is significant reduced by BET1 knockdown"

The authors have made all requested editorial changes.

Accepted **19th Oct 2021**

We are pleased to inform you that your manuscript is accepted for publication and is now being sent to our publisher to be included in the next available issue of EMBO Molecular Medicine.

EMBO PRESS

YOU MUST COMPLETE ALL CELLS WITH A PINK BACKGROUND ↓

PLEASE NOTE THAT THIS CHECKLIST WILL BE PUBLISHED ALONGSIDE YOUR PAPER

Corresponding Author Name: Carsten Bonnemann & Michael Schwake

Journal Submitted to: EMBO Molecular Medicine

Manuscript Number: EMM-2020-13787-V2

Reporting Checklist For Life Sciences Articles (Rev. June 2017)

This checklist is used to ensure good reporting standards and to improve the reproducibility of published results. These guidelines are consistent with the Principles and Guidelines for Reporting Preclinical Research issued by the NIH in 2014. Please follow the journal's authorship guidelines in preparing your manuscript.

A- Figures
1. Data
The data shown in figures should satisfy the following conditions:

- the data were obtained and processed according to the field's best practice and are presented to reflect the results of the experiments in an accurate and unbiased manner.
- figure panels include only data points, measurements or observations that can be compared to each other in a scientifically meaningful way.
- graphs include clearly labeled error bars for independent experiments and sample sizes. Unless justified, error bars should not be shown for technical replicates.
- if n< 5, the individual data points from each experiment should be plotted and any statistical test employed should be justified
- Source Data should be included to report the data underlying graphs. Please follow the guidelines set out in the author ship guidelines on Data Presentation.

2. Captions

Each figure caption should contain the following information, for each panel where they are relevant:

- a specification of the experimental system investigated (eg cell line, species name).
- the assay(s) and method(s) used to carry out the reported observations and measurements
- an explicit mention of the biological and chemical entity(ies) that are being measured.
- an explicit mention of the biological and chemical entity(ies) that are altered/varied/perturbed in a controlled manner.

- the exact sample size (n) for each experimental group/condition, given as a number, not a range;
- a description of the sample collection allowing the reader to understand whether the samples represent technical or biological replicates (including how many animals, litters, cultures, etc.).
- a statement of how many times the experiment shown was independently replicated in the laboratory.
- definitions of statistical methods and measures:
  - common tests, such as t-test (please specify whether paired vs. unpaired), simple χ2 tests, Wilcoxon and Mann-Whitney tests, can be unambiguously identified by name only, but more complex techniques should be described in the methods section;
  - are tests one-sided or two-sided?
  - are there adjustments for multiple comparisons?
  - exact statistical test results, e.g., P values = x but not P values < x;
  - definition of 'center values' as median or average;
  - definition of error bars as s.d. or s.e.m.

Any descriptions too long for the figure legend should be included in the methods section and/or with the source data.

In the pink boxes below, please ensure that the answers to the following questions are reported in the manuscript itself. Every question should be answered. If the question is not relevant to your research, please write NA (non applicable). We encourage you to include a specific subsection in the methods section for statistics, reagents, animal models and human subjects.

USEFUL LINKS FOR COMPLETING THIS FORM

http://www.antibodypedia.com
http://1degreebio.org
http://www.equator-network.org/reporting-guidelines/improving-bioscience-research-repor
http://grants.nih.gov/grants/olaw/olaw.htm
http://www.mrc.ac.uk/Ourresearch/Ethicsresearchguidance/Useofanimals/index.htm
http://ClinicalTrials.gov
http://www.consort-statement.org
http://www.consort-statement.org/checklists/view/32-consort/66-title
http://www.equator-network.org/reporting-guidelines/reporting-recommendations-for-tumo
http://datadryad.org
http://figshare.com
http://www.ncbi.nlm.nih.gov/gap
http://www.ebi.ac.uk/ega
http://biomodels.net/
http://biomodels.net/miriam/
http://jjj.biochem.sun.ac.za
https://osp.od.nih.gov/biosafety-and-emerging-biotechnology/
http://www.selectagents.gov/

B- Statistics and general methods

Please fill out these boxes ↓ (Do not worry if you cannot see all your text once you press return)

1.a. How was the sample size chosen to ensure adequate power to detect a pre-specified effect size?	No sample size calculations were performed as we identified and collected as many samples as available (for the clinical section of the study) or feasible (for the experimental section). All experiments were completed at least in triplicates.
1.b. For animal studies, include a statement about sample size estimate even if no statistical methods were used.	NA
2. Describe inclusion/exclusion criteria if samples or animals were excluded from the analysis. Were the criteria pre-established?	NA
3. Were any steps taken to minimize the effects of subjective bias when allocating animals/samples to treatment (e.g. randomization procedure)? If yes, please describe.	For the Golgi reconstitution assay images and samples were randomized to avoid bias
For animal studies, include a statement about randomization even if no randomization was used.	NA
4.a. Were any steps taken to minimize the effects of subjective bias during group allocation or/and when assessing results (e.g. blinding of the investigator)? If yes please describe.	For the Golgi reconstitution assay investigators who performed the analysis were blinded to avoid bias
4.b. For animal studies, include a statement about blinding even if no blinding was done	NA
5. For every figure, are statistical tests justified as appropriate?	Yes
Do the data meet the assumptions of the tests (e.g., normal distribution)? Describe any methods used to assess it.	Yes, D`Agostino and Pearson, Shapiro-Wilk and KS normality tests were performed to check for gaussian distribution of data sets n>5, done with GraphPad 7
Is there an estimate of variation within each group of data?	Yes, checked by reviewing column statistics with GraphPad 7

Is the variance similar between the groups that are being statistically compared?	Yes, checked by reviewing column statistics with GraphPad 7

C- Reagents

6. To show that antibodies were profiled for use in the system under study (assay and species), provide a citation, catalog number and/or clone number, supplementary information or reference to an antibody validation profile. e.g., Antibodypedia (see link list at top right), 1DegreeBio (see link list at top right).	: anti-BET1 (SC-136390, Santa Cruz), anti-ERGIC-53 (13364-1-AP, Proteintech) or (EPR6979, Abcam), anti-GOSR2 (170003, Synaptic Systems), anti-HA (12CA5, Roche), anti-GAPDH (CB1001, Millipore), SEC22b (A304-601A, Bethyl Laboratories), anti-Syntaxin-5 (SC-365124, Santa Cruz), anti-GM130 (Cell Signaling; mouse), α-GM130 (Abcam), anti-α-Dystroglycan (IIH6C4, Millipore 05-593) and α-PDI (Sigma Aldrich), α- mouse and α-rabbit Cy2 and Cy3 from Dianonva).
7. Identify the source of cell lines and report if they were recently authenticated (e.g., by STR profiling) and tested for mycoplasma contamination.	Banked cell lines from patients with BET1-related disease and controls. All cell lines are routinely tested for mycoplasma contamination by PCR.

* for all hyperlinks, please see the table at the top right of the document

D- Animal Models

8. Report species, strain, gender, age of animals and genetic modification status where applicable. Please detail housing and husbandry conditions and the source of animals.	NA
9. For experiments involving live vertebrates, include a statement of compliance with ethical regulations and identify the committee(s) approving the experiments.	NA
10. We recommend consulting the ARRIVE guidelines (see link list at top right) (PLoS Biol. 8(6), e1000412, 2010) to ensure that other relevant aspects of animal studies are adequately reported. See author guidelines, under 'Reporting Guidelines'. See also: NIH (see link list at top right) and MRC (see link list at top right) recommendations. Please confirm compliance.	NA

E- Human Subjects

11. Identify the committee(s) approving the study protocol.	National Institute of Neurological Disorders and Stroke, National Institutes of Health
12. Include a statement confirming that informed consent was obtained from all subjects and that the experiments conformed to the principles set out in the WMA Declaration of Helsinki and the Department of Health and Human Services Belmont Report.	Informed consent for study procedures and photographs were obtained by a qualified investigator (protocol 12-N-0095 approved by the National Institute of Neurological Disorders and Stroke, National Institutes of Health). Experiments conformed to the principles set out in the WMA Declaration of Helsinki and the Department of Health and Human Services Belmont Report.
13. For publication of patient photos, include a statement confirming that consent to publish was obtained.	NA
14. Report any restrictions on the availability (and/or on the use) of human data or samples.	Human data and samples can be shared with a Material Transfer Agreement in place
15. Report the clinical trial registration number (at ClinicalTrials.gov or equivalent), where applicable.	NCT01568658
16. For phase II and III randomized controlled trials, please refer to the CONSORT flow diagram (see link list at top right) and submit the CONSORT checklist (see link list at top right) with your submission. See author guidelines, under 'Reporting Guidelines'. Please confirm you have submitted this list.	NA
17. For tumor marker prognostic studies, we recommend that you follow the REMARK reporting guidelines (see link list at top right). See author guidelines, under 'Reporting Guidelines'. Please confirm you have followed these guidelines.	NA

F- Data Accessibility

18: Provide a "Data Availability" section at the end of the Materials & Methods, listing the accession codes for data generated in this study and deposited in a public database (e.g. RNA-Seq data: Gene Expression Omnibus GSE39462, Proteomics data: PRIDE PXD000208 etc.) Please refer to our author guidelines for 'Data Deposition'.<br><br>Data deposition in a public repository is mandatory for:<br>a. Protein, DNA and RNA sequences<br>b. Macromolecular structures<br>c. Crystallographic data for small molecules<br>d. Functional genomics data<br>e. Proteomics and molecular interactions	Phenotype data and candidate gene/variant information are shared through Seqr with the Matchmaker Exchange system (https://www.matchmakerexchange.org/).<br><br>RNA-Seq data: dbGaP phs001272.v1.p1 (https://www.ncbi.nlm.nih.gov/projects/gap/cgi-bin/study.cgi?study_id=phs001272.v1.p1)<br><br>WES data (P1): AnVIL, AnVIL_CMG_Broad_Muscle_Bonnemann_WES. 9(https://anvil.terra.bio/#workspaces/anvil-datastorage/AnVIL_CMG_Broad_Muscle_Bonnemann_WES)
19. Deposition is strongly recommended for any datasets that are central and integral to the study; please consider the journal's data policy. If no structured public repository exists for a given data type, we encourage the provision of datasets in the manuscript as a Supplementary Document (see author guidelines under 'Expanded View' or in unstructured repositories such as Dryad (see link list at top right) or Figshare (see link list at top right).	All data generated in this study are available upon request from the authors.
20. Access to human clinical and genomic datasets should be provided with as few restrictions as possible while respecting ethical obligations to the patients and relevant medical and legal issues. If practically possible and compatible with the individual consent agreement used in the study, such data should be deposited in one of the major public access-controlled repositories such as dbGAP (see link list at top right) or EGA (see link list at top right).	Data is deposited in dbGAP and is also available on request with appropriate Material Transfer Agreements in place.
21. Computational models that are central and integral to a study should be shared without restrictions and provided in a machine-readable form. The relevant accession numbers or links should be provided. When possible, standardized format (SBML, CellML) should be used instead of scripts (e.g. MATLAB). Authors are strongly encouraged to follow the MIRIAM guidelines (see link list at top right) and deposit their model in a public database such as Biomodels (see link list at top right) or JWS Online (see link list at top right). If computer source code is provided with the paper, it should be deposited in a public repository or included in supplementary information.	NA

G- Dual use research of concern

22. Could your study fall under dual use research restrictions? Please check biosecurity documents (see link list at top right) and list of select agents and toxins (APHIS/CDC) (see link list at top right). According to our biosecurity guidelines, provide a statement only if it could.	No
